# Supplementary material for: LKRSDH-dependent histone modifications of insulin-like peptide sites contribute to age-related circadian rhythm changes
Source: Nat Commun. 2024 Apr 18;15:3336. doi: 10.1038/s41467-024-47740-4 (PMC11026460; doi:10.1038/s41467-024-47740-4)
Supplement: Supplementary file 1 — Supplementary Information [file 41467_2024_47740_MOESM1_ESM.pdf]

Article Title: LKRSDH-dependent histone modifications of insulin-like peptide sites contribute to age-related circadian rhythm changes

Supplementary Material

Supplementary Figure 1

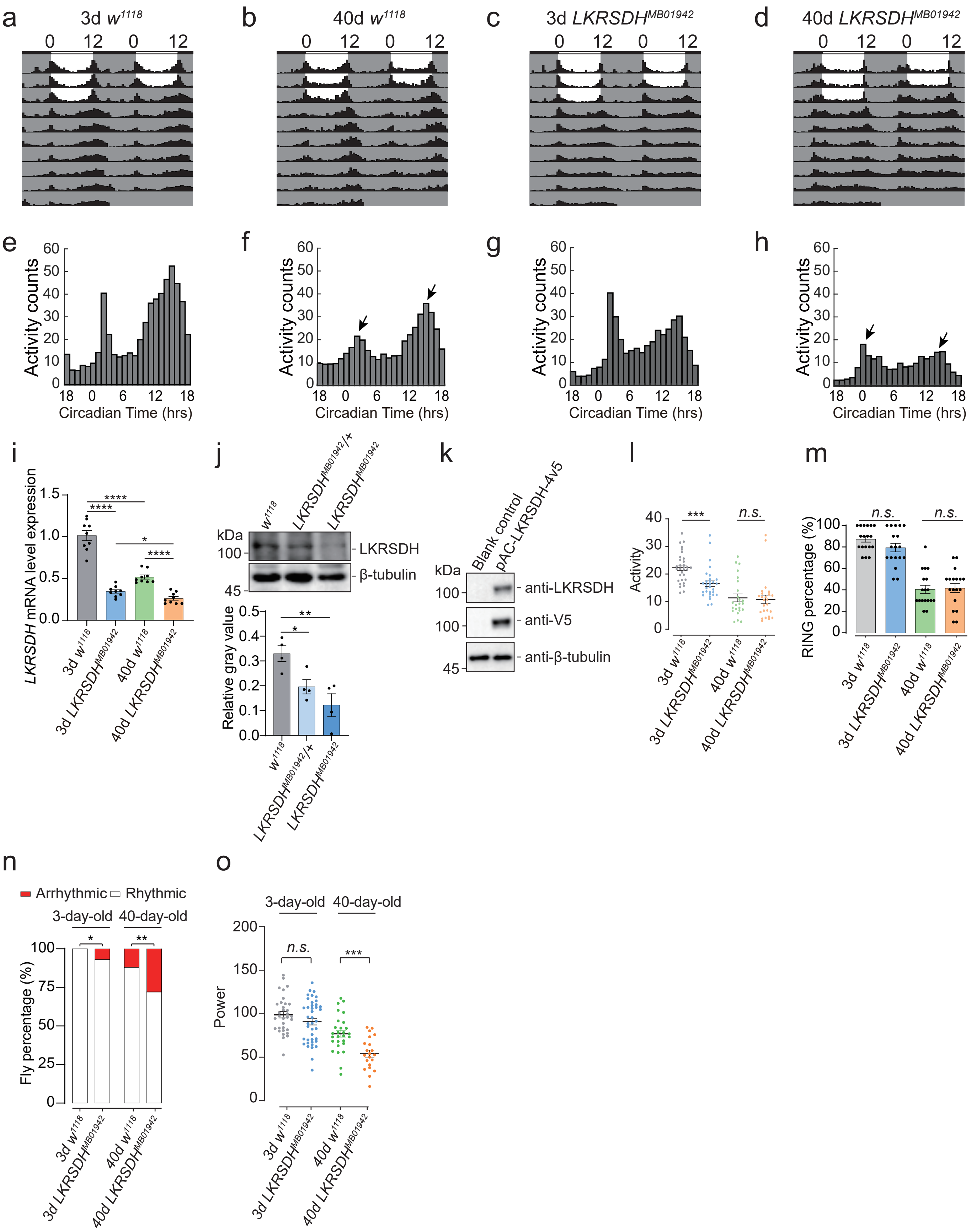

Supplementary Fig. 1. *LKRSDH* is involved in circadian rhythm regulation in aging adult *Drosophila*.

a-d The representative locomotor activity (**a-d;** n = 16, 16, 16, 16). Grey shading indicates constant darkness (DD), black columns represent locomotor activity of flies. **e-h** Average activity count data under DD, corresponding to (**a-d**). The arrow indicates the characteristic morning-evening activity peak pattern of flies. **i** Quantitative RT-PCR of *LKRSDH. n* = 3 biologically independent experiments. Data are presented as mean ± SEM. P-values from unpaired two-tailed Student's *t*-test are indicated, where *P < 0.05 and ****P < 0.0001. All the P-values are listed in Supplementary Data 3. **j** Western blot (WB) analysis (*n* = 4 biologically independent experiments). WB gray values were measured with Image J. P-values from unpaired two-tailed Student's *t*-test are indicated, where *P < 0.05 and **P < 0.01. Data are presented as mean ± SEM. **k** The specificity of the LKRSDH antibody was confirmed through WB. **l** Number of activities per 30 min. Data are presented as mean ± SEM. P-values from unpaired two-tailed Student's *t*-test are indicated, where *n.s.* indicates no significant difference and ***P < 0.001. All the P-values are listed in Supplementary Data 3. *n* = 3 biologically independent experiments. **m** RING assay. RING, Rapid Iterative Negative Geotaxis. Data are presented as mean ± SEM. P-values from unpaired two-tailed Student's *t*-test are indicated, where *n.s.* indicates no significant difference. All the P-values are listed in Supplementary Data 3. *n* = 3 biologically independent experiments. **n** Percentage of rhythmic and arrhythmic flies under LD conditions (**n;** n = 33, 44, 32, 29; *LKRSDH* homozygous mutant flies). P-values from two-sided Fisher's exact test are indicated, where *P < 0.05 and **P < 0.01. All the P-values are listed in Supplementary Data 3. **o** Circadian power value of flies with rhythm, corresponding to (**n**). Data are presented as mean ± SEM. P-values from unpaired two-tailed Student's *t*-test are indicated, where *n.s.* indicates no significant difference and ** indicates P < 0.01. All the P-values are listed in Supplementary Data 3. Source data are provided as a Source Data file.

Supplementary Figure 2

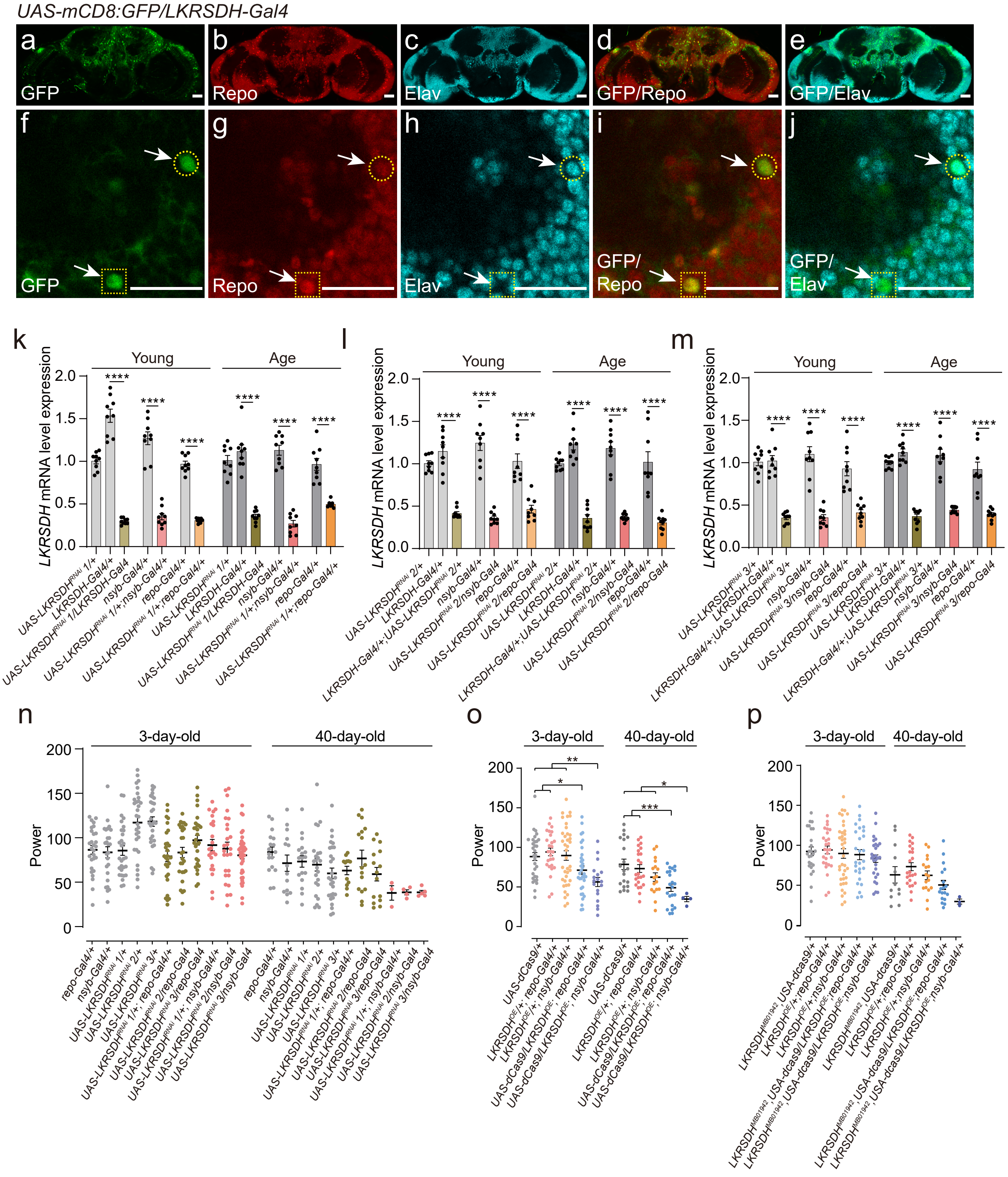

Supplementary Fig. 2. *LKRSDH* is expressed in both neurons and glial cells.

a-j LKRSDH represented by GFP in *LKRSDH-Gal4* was expressed in the same set of cells with neurons represented with Elav antibody staining or glial cells represented with Repo antibody staining. Heads of UAS-mCD8:GFP/LKRSDH-Gal4 were dissected at ZT8. Cells surrounded by rectangle and circle shapes represent the co-localization of LKRSDH with glial cells and neurons, respectively. Scale bar = 10 µm. The arrow highlights the co-localization of LKRSDH with neurons or glial cells. The images presented are from one of the three biological repeats. **k-m** The knockdown efficiency of *LKRSDH RNAi* was shown. Data are presented as mean ± SEM. P-values from unpaired two-tailed Student's *t*-test are indicated, where *P < 0.05 and ****P < 0.0001. All the P-values are listed in Supplementary Data 3. *n* = 3 biologically independent experiments. **n-p** Circadian power value of flies with rhythm, corresponding to **Fig. 2p-r**, respectively. Data are presented as mean ± SEM. P-values from unpaired two-tailed Student's *t*-test are indicated, *P < 0.05, **P < 0.01 and ***P < 0.001. If P > 0.05, it will not be presented in the figures. All the P-values are listed in Supplementary Data 3. Source data are provided as a Source Data file.

Supplementary Figure 3

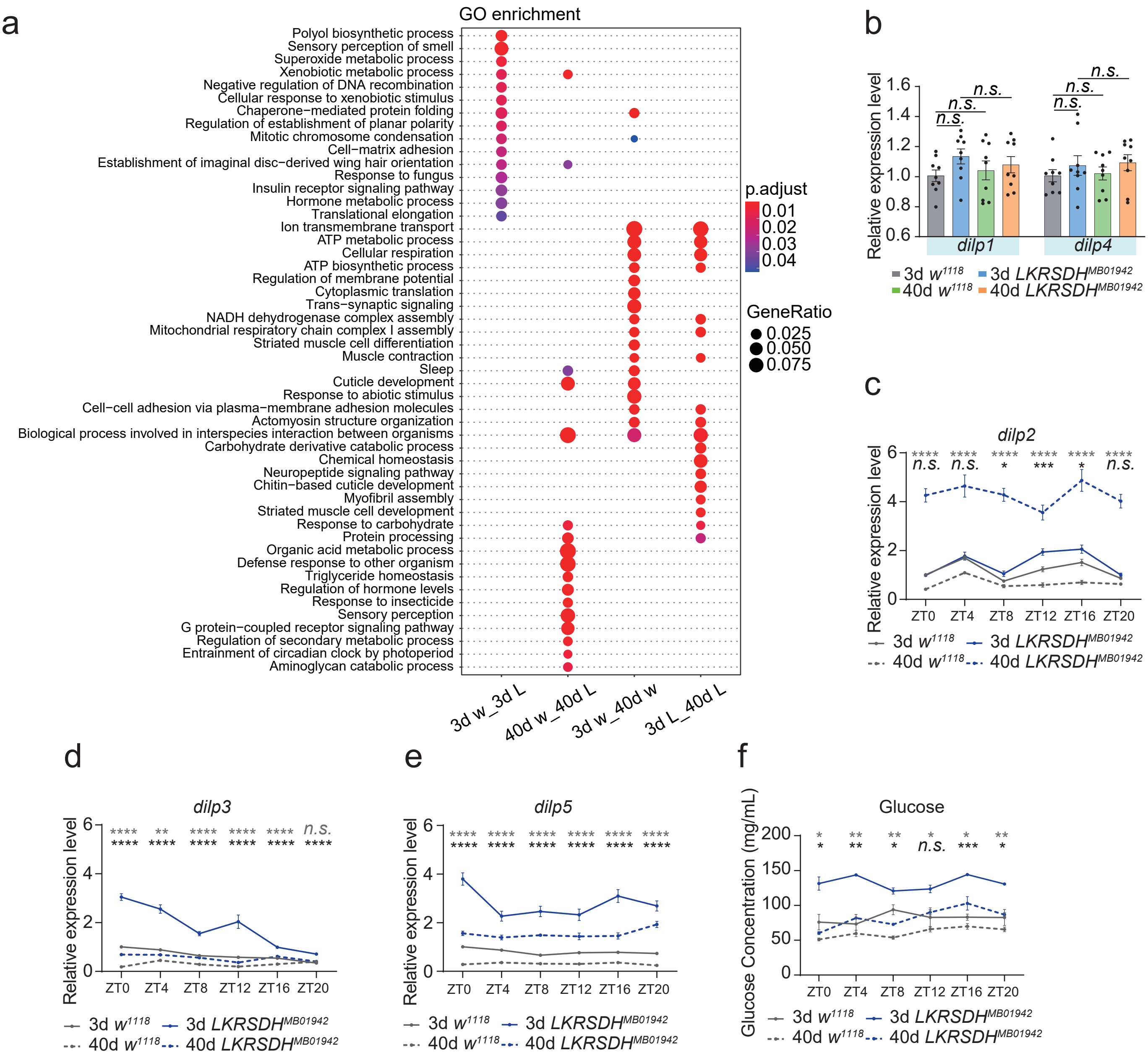

Supplementary Fig. 3. Analysis of downstream genes of *LKRSDH*.

a GO enrichment analysis was performed for genes regulated by *LKRSDH* or altered during aging, as identified by RNA-seq. GO enrichment in particular pathway was assessed using adjusted one-tailed *P* values and GeneRatio. **b** Quantitative RT-PCR of *dilp1* and *dilp4* in heads of 3-day-old w^{1118} control, 3-day-old $LKRSDH^{MB01942}$ mutant, 40-day-old w^{1118} control and 40-day-old $LKRSDH^{MB01942}$ mutant. Data are presented as mean ± SEM. P-values from unpaired two-tailed Student's *t*-test are indicated, where *n.s.* indicates no significant difference. All the P-values are listed in Supplementary Data 3. *n* = 3 biologically independent experiments. **c-e** Circadian dynamics of *dilp2* (**c**), *dilp3* (**d**) and *dilp5* (**e**) in fly heads. Data are presented as mean ± SEM. P-values from unpaired two-tailed Student's *t*-test are indicated, where *n.s.* indicates no significant difference, *P < 0.05, **P < 0.01, ***P < 0.001 and ****P < 0.0001. All the P-values are listed in Supplementary Data 3. *n* = 3 biologically independent experiments. **f** Circadian dynamics of glucose in whole flies. Data are presented as mean ± SEM. P-values from unpaired two-tailed Student's *t*-test are indicated, where *n.s.* indicates no significant difference, *P < 0.05, **P < 0.01, ***P < 0.001 and ****P < 0.0001. All the P-values are listed in Supplementary Data 3. *n* = 3 biologically independent experiments. Source data are provided as a Source Data file.

Supplementary Figure 4

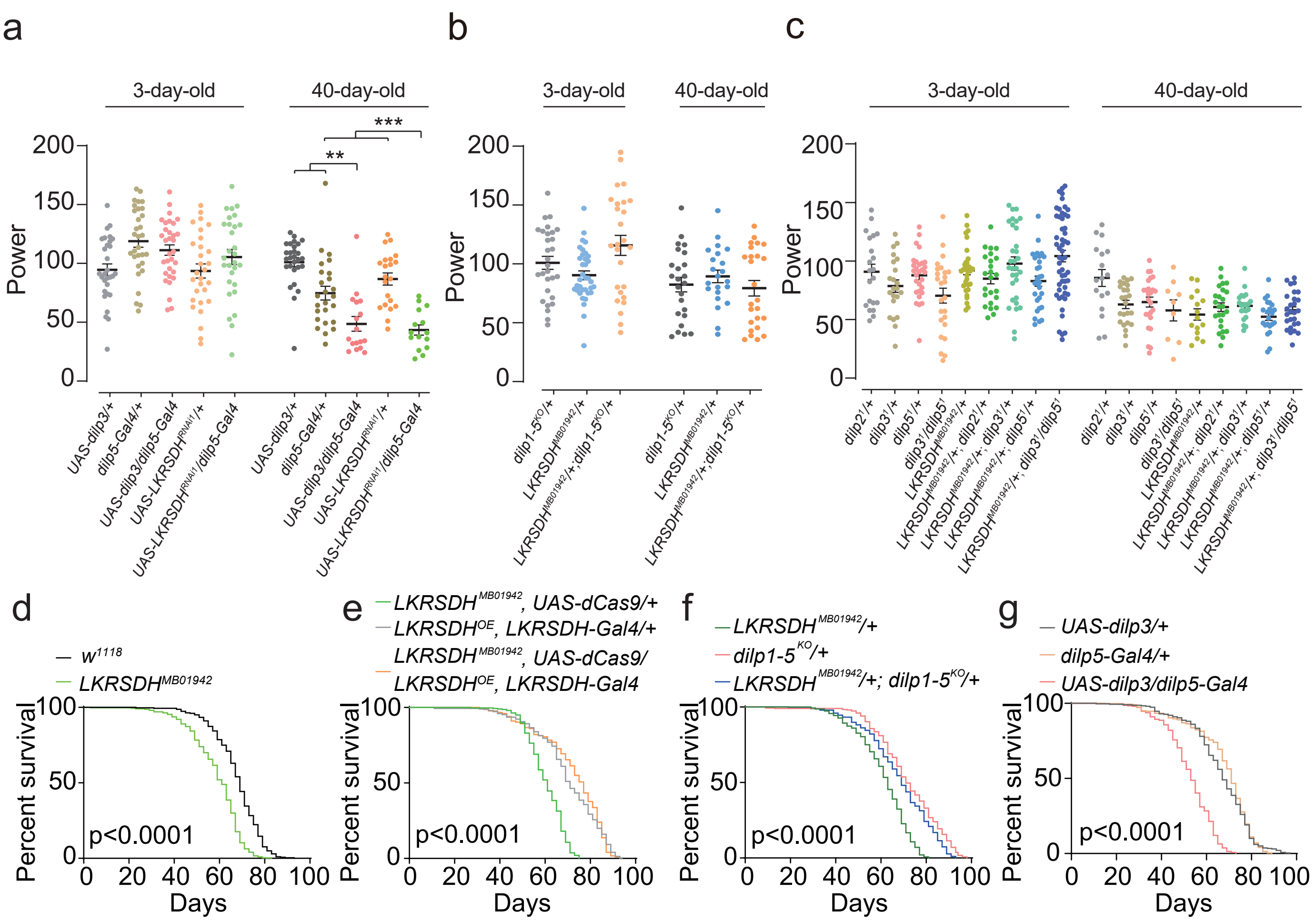

Supplementary Fig. 4. *LKRSDH* affected *Drosophila* lifespan through regulation of *dilps*.

a-c Circadian power value of flies with rhythm, corresponding to Fig. 4**b-d**. Data are presented as mean ± SEM. P-values from unpaired two-tailed Student's *t*-test are indicated, where **P < 0.01 and ***P < 0.001. If P > 0.05, it will not be presented in the figures. All the P-values are listed in Supplementary Data 3. **d** Survival curves of w^{1118} (black; n=300) and $LKRSDH^{MB01942}$ (*LKRSDH* homozygous mutant flies, green; n=300). *P* values from Log-rank (Mantel-ox) test are indicated in the plots. **e** Survival curves of $LKRSDH^{MB01942}$*, UAS-dCas9/+* (*LKRSDH* heterozygotes mutant flies, green; n=300), *LKRSDH-Gal4,* $LKRSDH^{OE}$*/+* (gray; n=300) and $LKRSDH^{MB01942}$*, UAS-dCas9/LKRSDH-Gal4,* $LKRSDH^{OE}$ (*LKRSDH* heterozygotes mutant flies, pale brown; n=300). *P* values from Log-rank (Mantel-ox) test are indicated in the plots. **f** Survival curves of $LKRSDH^{MB01942}$/+ (*LKRSDH* heterozygotes mutant flies, green; n=300), *dilp 1-*5^{KO}/+ (pink; n=300) and $LKRSDH^{MB01942}$/+; *dilp 1-*5^{KO}/+ (*LKRSDH* heterozygotes mutant flies, blue; n=300). *P* values from Log-rank (Mantel-ox) test are indicated in the plots. **g** Survival curves of *UAS-dilp3/+* (black; n=300), *dilp5-Gal4*/+ (orange; n=300) and *UAS-dilp3/dilp5-Gal4* (pink; n=300). *P* values from Log-rank (Mantel-ox) test are indicated in the plots. Source data are provided as a Source Data file.

Supplementary Figure 5

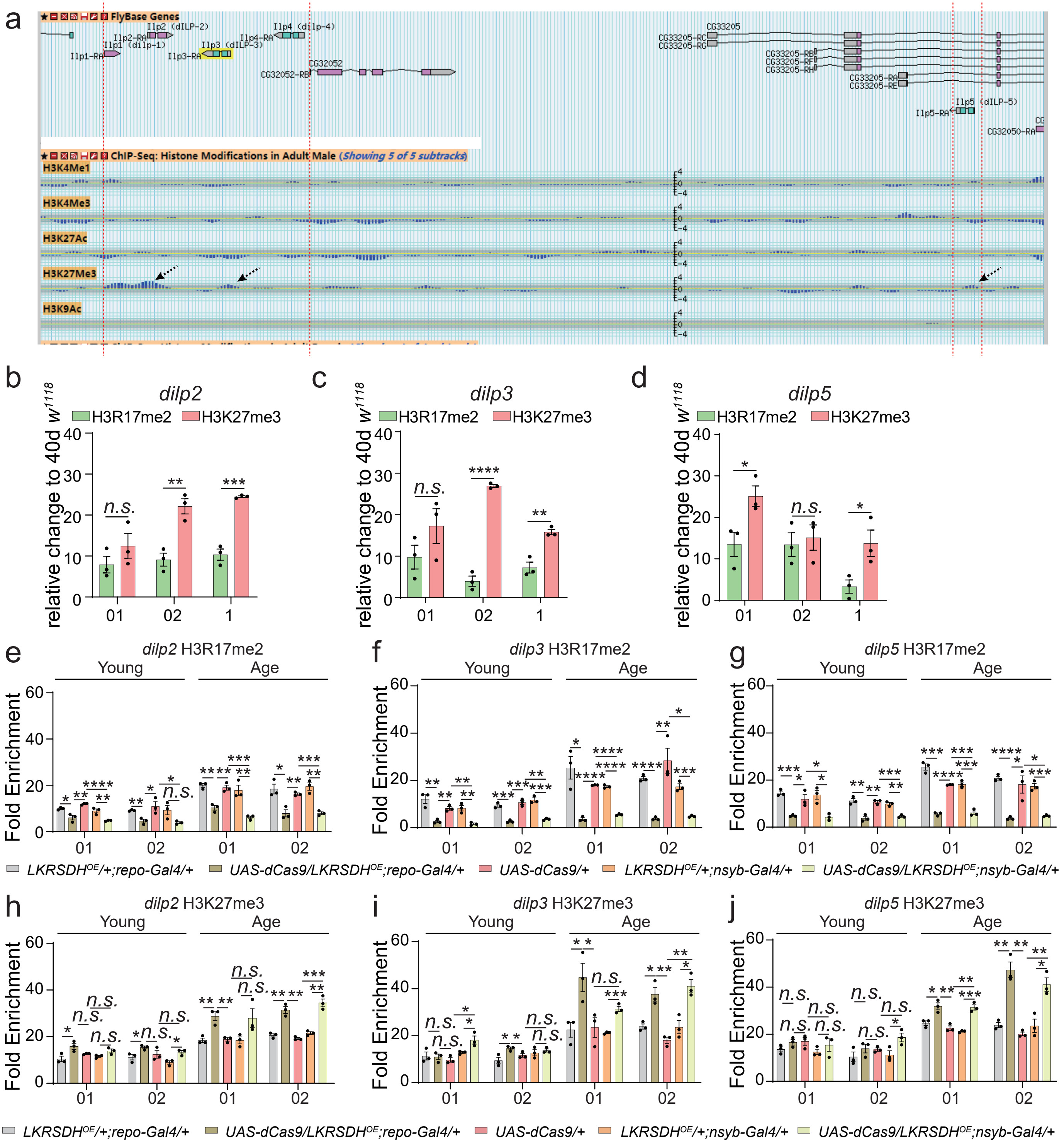

Supplementary Fig. 5. Histone modification features of *ilp* gene locus were examined, with H3R17me2 levels tested in the indicated genotypes.

a The histone modification profile of *dilps* locus in the ModENCODE database are presented. The arrow indicates the H3K27me3 modification on *dilps*. **b-d** Relative changes in H3K27me3 and H3R17me2 on the gene body and promoter regions of *dilp2* (**b**), *dilp3* (**c**) and *dilp5* (**d**) after $LKRSDH^{MB01942}$ mutation and aging were examined. Data are presented as mean ± SEM. P-values from unpaired two-tailed Student's *t*-test are indicated, where *n.s.* indicates no significant difference, *P < 0.05, **P < 0.01, ***P < 0.001 and ****P < 0.0001. All the P-values are listed in Supplementary Data 3. **e-j** The fold enrichments of two histone modifications H3R17me2 and H3K27me3 on promoter and gene body of *dilp2*, *dilp3* and *dilp5* were examined. Data are presented as mean ± SEM. P-values from unpaired two-tailed Student's *t*-test are indicated, where *n.s.* indicates no significant difference, *P < 0.05, **P < 0.01, ***P < 0.001 and ****P < 0.0001. All the P-values are listed in Supplementary Data 3. *n* = 3 biologically independent experiments. Source data are provided as a Source Data file.

Supplementary Figure 6

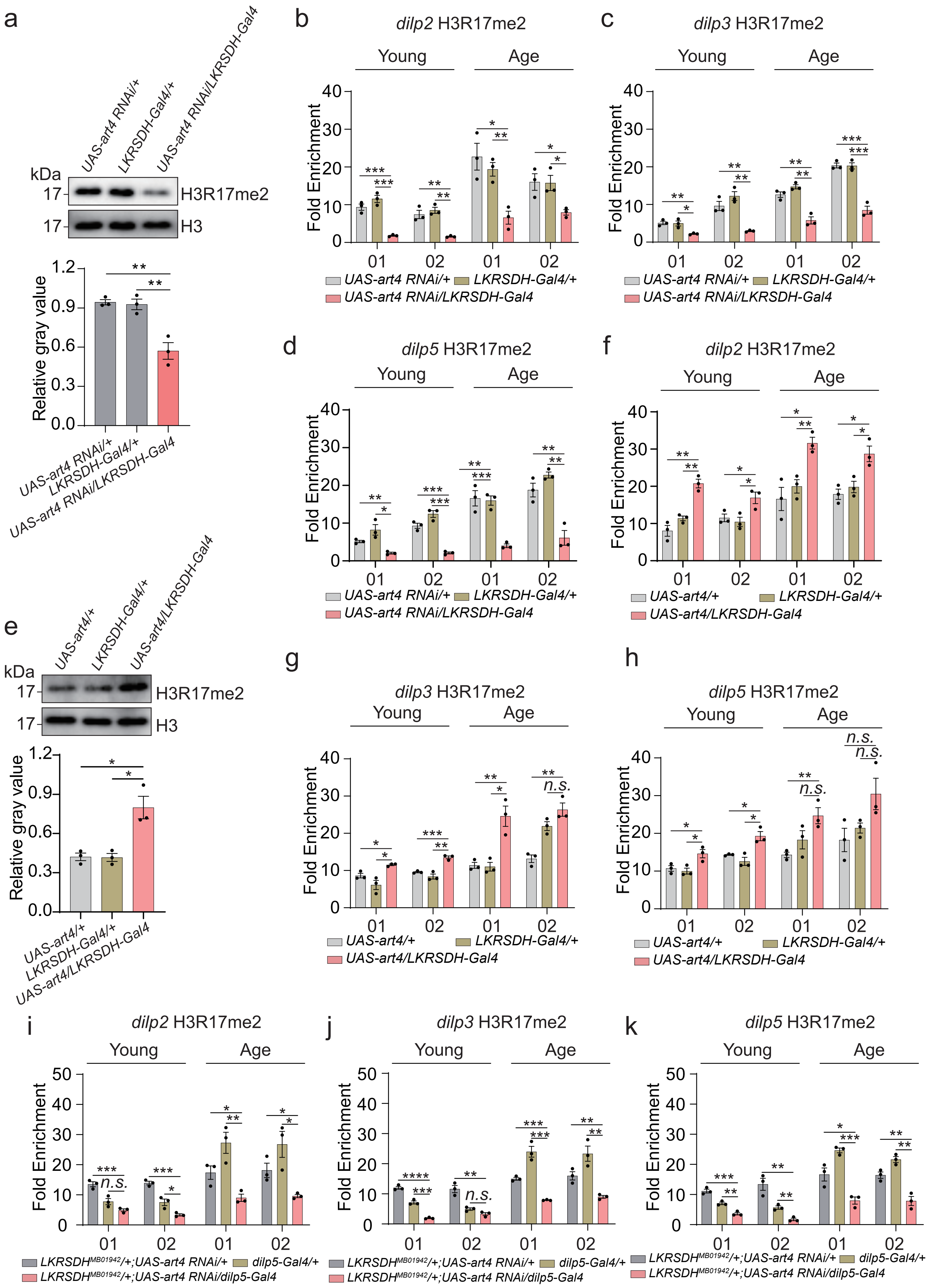

Supplementary Fig. 6. *art4* regulates H3R17me2.

a Western blot analysis of H3R17me2 was conducted, with the antibody against H3 served as the loading control. The relative gray value of Western blot was measured using Image J. Data are presented as mean ± SEM. Statistical differences were assessed using unpaired two-tailed Student's *t*-test. ** indicates $P < 0.01$. The images presented are from one of the three biological repeats. All the P-values are listed in Supplementary Data 3. **b-d** The fold enrichment of H3R17me2 at the promoter and gene body of *dilp2*, *dilp3* and *dilp5* locus was examined. Data are presented as mean ± SEM. P-values from unpaired two-tailed Student's *t*-test are indicated, where *n.s.* indicates no significant difference, *$P < 0.05$, **$P < 0.01$ and ***$P < 0.001$. All the P-values are listed in Supplementary Data 3. $n = 3$ biologically independent experiments. **e** Western blot analysis of H3R17me2 was conducted, with the antibody against H3 served as the loading control. The relative gray value of Western blot was measured using Image J. Data are presented as mean ± SEM. Statistical differences were assessed using unpaired two-tailed Student's *t*-test. * indicates $P < 0.05$. The images presented are from one of the three biological repeats. All the P-values are listed in Supplementary Data 3. **f-k** The fold enrichment of H3R17me2 at the promoter and gene body of *dilp2*, *dilp3* and *dilp5* locus was examined. Data are presented as mean ± SEM. P-values from unpaired two-tailed Student's *t*-test are indicated, where *n.s.* indicates no significant difference, *$P < 0.05$, **$P < 0.01$, ***$P < 0.001$ and ****$P < 0.0001$. All the P-values are listed in Supplementary Data 3. $n = 3$ biologically independent experiments. Source data are provided as a Source Data file.

Supplementary Figure 7

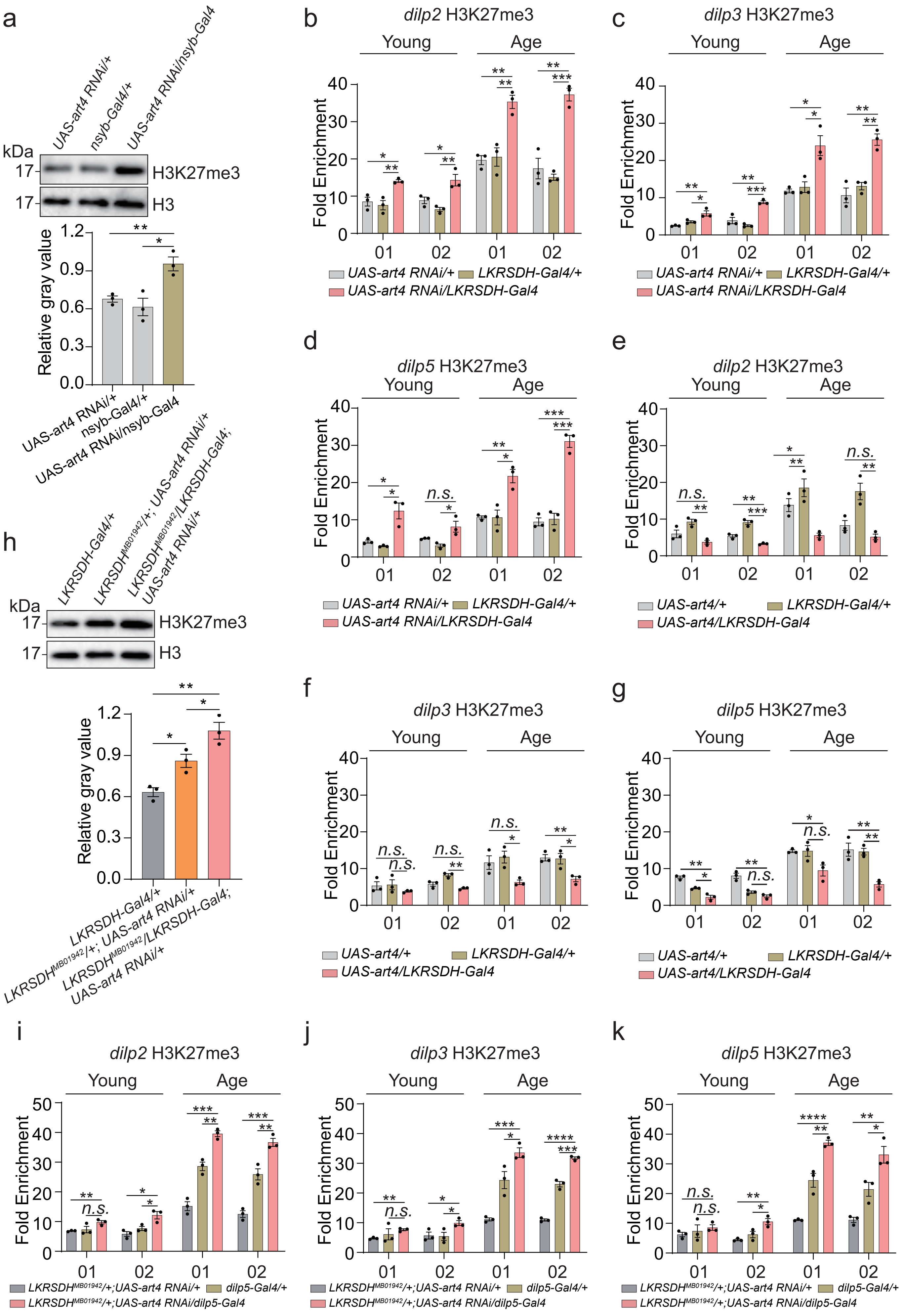

Supplementary Fig. 7. *art4* regulates H3K27me3.

a Western blot analysis of H3K27me3 was conducted, with the antibody against H3 served as the loading control. The relative gray value of Western blot was measured using Image J. Data are presented as mean ± SEM. Statistical differences were assessed using unpaired two-tailed Student's *t*-test. * indicates $P < 0.05$ and ** indicates $P < 0.01$. The images presented are from one of the three biological repeats. All the P-values are listed in Supplementary Data 3. **b-g** The fold enrichment of H3K27me3 at the promoter and gene body of *dilp2*, *dilp3* and *dilp5* locus was examined. Data are presented as mean ± SEM. P-values from unpaired two-tailed Student's *t*-test are indicated, where *n.s.* indicates no significant difference, *$P < 0.05$, **$P < 0.01$ and ***$P < 0.001$. All the P-values are listed in Supplementary Data 3. $n = 3$ biologically independent experiments. **h** Western blot analysis of H3K27me3 was conducted, with the antibody against H3 served as the loading control. The relative gray value of Western blot was measured using Image J. Data are presented as mean ± SEM. Statistical differences were assessed using unpaired two-tailed Student's *t*-test. * indicates $P < 0.05$ and ** indicates $P < 0.01$. The images presented are from one of the three biological repeats. All the P-values are listed in Supplementary Data 3. **i-k** The fold enrichment of H3K27me3 at the promoter and gene body of *dilp2*, *dilp3* and *dilp5* locus was examined. Data are presented as mean ± SEM. P-values from unpaired two-tailed Student's *t*-test are indicated, where *n.s.* indicates no significant difference, *$P < 0.05$, **$P < 0.01$, ***$P < 0.001$ and ****$P < 0.0001$. All the P-values are listed in Supplementary Data 3. $n = 3$ biologically independent experiments. Source data are provided as a Source Data file.

Supplementary Figure 8

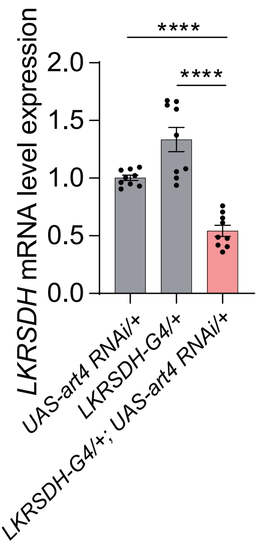

Supplementary Fig. 8. The downregulation of *art4* led to a decrease in *LKRSDH* levels.

Quantitative RT-PCR of *LKRSDH* in heads of UAS-*art4 RNAi*/+, *LKRSDH*-Gal4/+ and *LKRSDH*-Gal4/+; UAS-*art4 RNAi/+*. Data are presented as mean ± SEM. P-values from unpaired two-tailed Student's *t*-test are indicated, where *n.s.* indicates no significant difference. All the P-values are listed in Supplementary Data 3. *n* = 3 biologically independent experiments. Source data are provided as a Source Data file.

Supplementary Figure 9

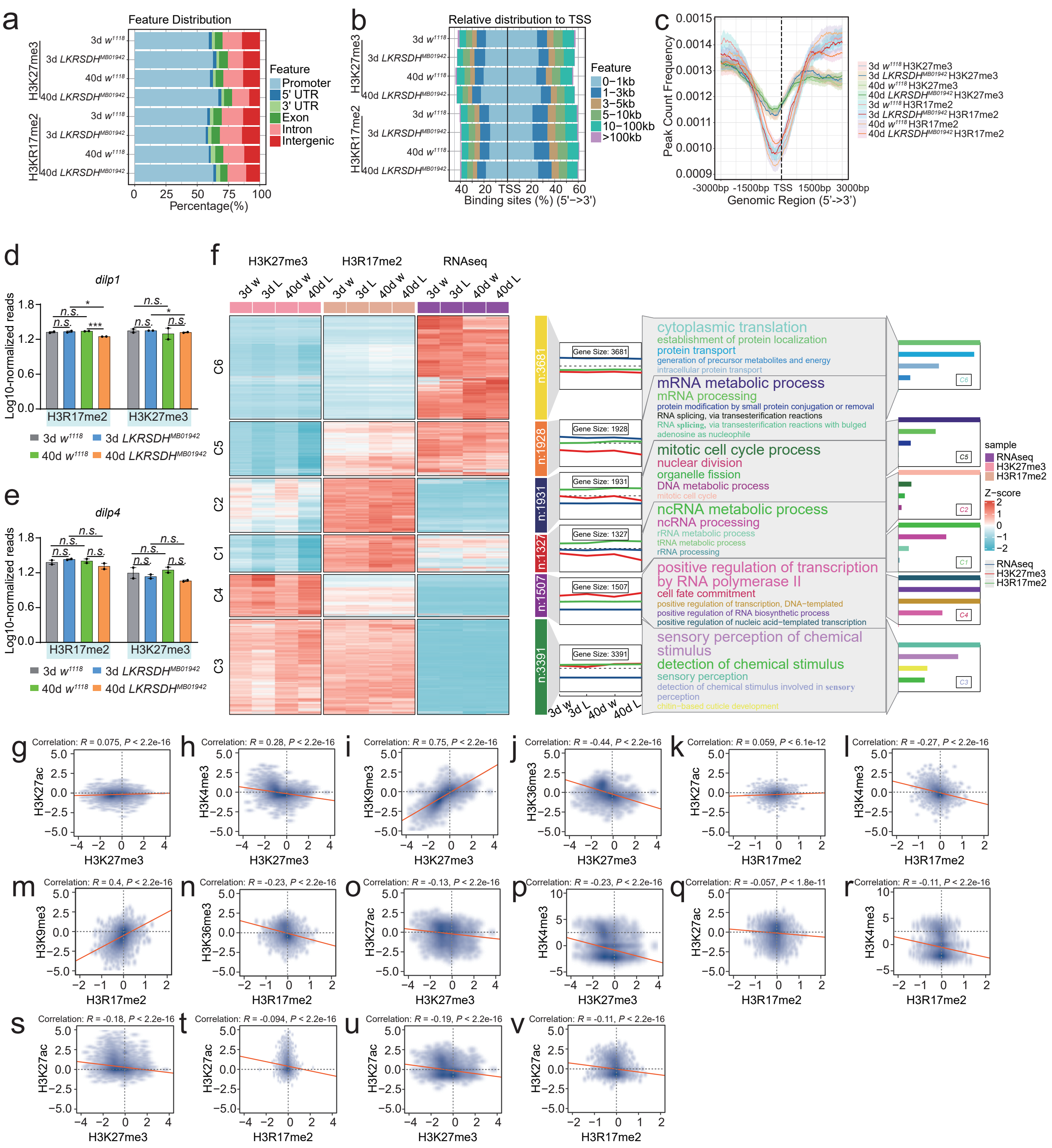

Supplementary Fig. 9. Global features of H3R17me2 and H3K27me3 landscape.

a Distribution of H3K27me3 and H3R17me2 peaks on annotated *Drosophila* genome. **b** The distribution of H3K27me3 and H3R17me2 peaks relative to TSS. **c** Average profile of IP/Input peaks around the TSS region (±3 kb). 95% confidence intervals were calculated for each gene cluster. **d**, **e** Normalized CHIP-seq read coverage of H3R17me2 and H3K27me3 over *dilp1* and *dilp4* locus. Data are presented as mean ± SEM. P-values from unpaired two-tailed Student's *t*-test are indicated, where *n.s.* indicates no significant difference, $^*P < 0.05$ and $^{***}P < 0.001$ indicated significant differences. All the P-values are listed in Supplementary Data 3. **f** Heatmaps showing the enrichment of H3R17me2 and H3K27me3, and the expression of the clustered gene groups (left panel). w represents w^{1118} and L represents $LKRSDH^{MB01942}$. Line showing overall alterations of H3R17me2 and H3K27me3 enrichment at each gene cluster, and the alterations in their expression (middle panel). The red line indicates the fraction of domains that is marked by H3K27me3, the green line represents the fraction of domains that is marked by H3R17me2, and the blue line indicates the fraction of domains by RNA-seq. The GO enrichment of different gene clusters is shown on the right panel. Columns represent different GO term sizes, and bars show the pvalue of genes enriched in the corresponding pathways. **g-v** Genome-wide correlation analysis of the data from this study with the published datasets. The correlation coefficient (*R*) is indicated. Source data are provided as a Source Data file.

Supplementary Figure 10

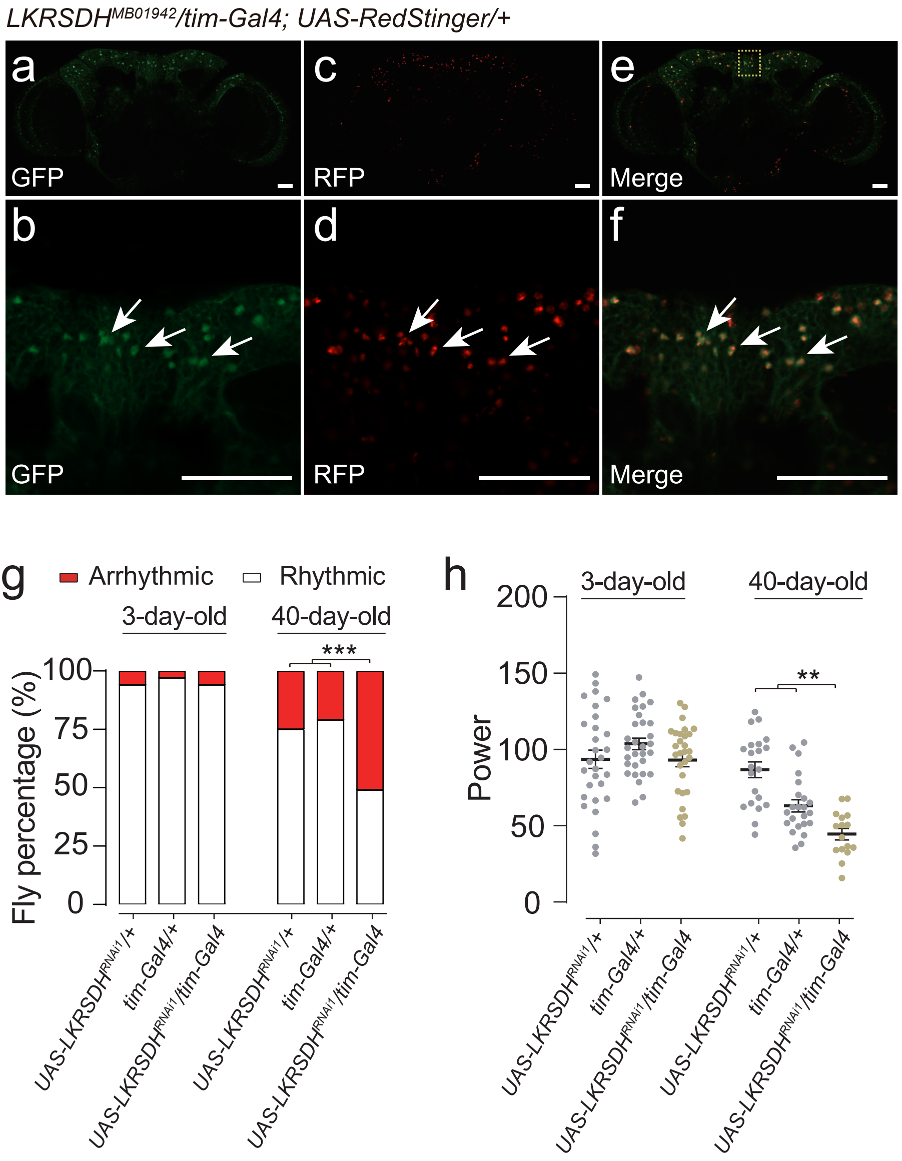

Supplementary Fig. 10. The expression of LKRSDH in clock neurons is necessary for maintaining the circadian rhythm in aged flies.

a-f LKRSDH represented by GFP in *LKRSDH*^{MB01942} was expressed in the same set of cells with *tim*-Gal4 driven *Redstinger*. The arrow indicates the co-localization of LKRSDH with TIM neurons. Scale bar = 10 μm. Heads of *LKRSDH*^{MB01942}*/tim*-Gal4*;UAS-RedStinger/+* were dissected at ZT8. The images presented are from one of the three biological repeats. **g** Percentage of rhythmic and arrhythmic flies (from left to right, bars represent n = 31, 31, 31, 28, 29 and 33 independent samples). P-values from two-sided Fisher's exact test are indicated, where ***P < 0.001. All the P-values are listed in Supplementary Data 3. **h** Circadian power value of flies with rhythm, corresponding to (**g**). Data are presented as mean ± SEM. P-values from unpaired two-tailed Student's *t*-test are indicated. If P > 0.05, it will not be presented in the figures. All the P-values are listed in Supplementary Data 3. Source data are provided as a Source Data file.

Supplementary table 1 Primers used in this study.

Primers	Sequence (5’-3’)
NotI-LKRSDH	AGCACAGTGGCGGCCGCATGTGGCGAGTGATTCAACTGC
HindIII-LKRSDH	GGCCCATAATAAGCTTATTTAGCCATCTGGAGGTTTCCGT
dilp1- forward	GGCTCCAGTCGCTGCTCAT
dilp1- reverse	GCCGCAGAGTTTGTGGTTT
dilp2- forward	AAGCCTTTGTCCTTCATCTC
dilp2- reverse	GATTGAGGGCGTCCAGAT
dilp3- forward	TTATGATCGGCGGTGTCCAG
dilp3- reverse	TTGGTCATTGCGTTGAAGCC
dilp4- forward	ATGAGCCTGATTAGACTGGG
dilp4- reverse	GCCTCGCCGCACATCTTT
dilp5- forward	ATGGACATGCTGAGGGTTGC
dilp5- reverse	ATCCAAATCCGCCAAGTGGT
LKRSDH- forward	GCAGAATGTCAAGGTCATAGTG
LKRSDH- reverse	ATCTATGGGCACCTGTTTCACT
art4- forward	AATACAAGGGAAGCTGCACG
art4- reverse	CACTAGATTGTCGGCGTCCA
rp49- forward	AGATCGTGAAGAAGCGCACCAAG
rp49- reverse	CACCAGGAACTTCTTGAATCCGG
dilp2 01 forward	CCACAATTTTCCAATAGCCA
dilp2 01 reverse	TCAAAAGTCAGGTTGTCAAA
dilp2 02 forward	ACATGTGTGTGGATCCAGAC
dilp2 02 reverse	CGAAAGCTAACTGATGATGTT
dilp2 1 forward	GCTGGCCAGCTCCACAGTGA
dilp2 1 reverse	TGCATCCTTGTAAGAACTTA
dilp3 01 forward	TAGTAGCTTGCCAAATGTGA
dilp3 01 reverse	TGCATCGAGTTCCCTTCGGC
dilp3 02 forward	ACAATCCAGATCCGTGCGCT
dilp3 02 reverse	GGTCTAGTTTCTCGTGAGGA
dilp3 1 forward	TGGCAGACACATACTACACA
dilp3 1 reverse	GTAGGCTAGGTAGCAGGATC
dilp5 01 forward	ACTATTTGCACGATAAGCC
dilp5 01 reverse	TTAATCGGATGCACCAAGGG
dilp5 02 forward	ATAAATTAATGATAGCACCC
dilp5 02 reverse	TTTCCGCCGCTGTCAGCCAT
dilp5 1 forward	CTGTTCCTGATCCCGCTCCTG
dilp5 1 reverse	GAATCCATTGGGACAGGCAA
