# Supplementary material for: LKRSDH-dependent histone modifications of insulin-like peptide sites contribute to age-related circadian rhythm changes
Source: Nat Commun. 2024 Apr 18;15:3336. doi: 10.1038/s41467-024-47740-4 (PMC11026460; doi:10.1038/s41467-024-47740-4)
Supplement: Supplementary file 3 — Description of Additional Supplementary Files [file 41467_2024_47740_MOESM3_ESM.pdf]

Description of Additional Supplementary Files:

Supplementary Data 1: Differentially expressed gene lists revealed by RNA-seq in samples of 3d w1118 v.s. 3d LKRSDHMB01942, 40d w1118 v.s. 40d LKRSDHMB01942, 3d w1118 v.s. 40d w1118 and 3d LKRSDHMB01942 v.s. 40d LKRSDHMB01942.

Differentially expressed genes with p.adjust < 0.05 are presented using adjusted P values. The analysis of differentially expressed genes was performed using a two-tailed hypothesis test.

Supplementary Data 2: GO and KEGG enrichment analysis of genes regulated by LKRSDH or altered during aging identified by RNA-seq.

GO and KEGG terms with p.adjust < 0.05 are shown. GO and KEGG enrichment were measured by adjusted one-tailed P values.

Supplementary Data 3: Statistic values indicated the significance of differences.
