# Supplementary material for: LKRSDH-dependent histone modifications of insulin-like peptide sites contribute to age-related circadian rhythm changes
Source: Nat Commun. 2024 Apr 18;15:3336. doi: 10.1038/s41467-024-47740-4 (PMC11026460; doi:10.1038/s41467-024-47740-4)
Supplement: Supplementary file 7 — Reporting Summary [file 41467_2024_47740_MOESM7_ESM.pdf]

nature portfolio

Corresponding author(s): Juan Du

Last updated by author(s): Mar 27, 2024

Reporting Summary

Nature Portfolio wishes to improve the reproducibility of the work that we publish. This form provides structure for consistency and transparency in reporting. For further information on Nature Portfolio policies, see our Editorial Policies and the Editorial Policy Checklist.

Statistics

For all statistical analyses, confirm that the following items are present in the figure legend, table legend, main text, or Methods section.

n/a	Confirmed	
☐	☒	The exact sample size (*n*) for each experimental group/condition, given as a discrete number and unit of measurement
☐	☒	A statement on whether measurements were taken from distinct samples or whether the same sample was measured repeatedly
☐	☒	The statistical test(s) used AND whether they are one- or two-sided<br>*Only common tests should be described solely by name; describe more complex techniques in the Methods section.*
☐	☒	A description of all covariates tested
☐	☒	A description of any assumptions or corrections, such as tests of normality and adjustment for multiple comparisons
☐	☒	A full description of the statistical parameters including central tendency (e.g. means) or other basic estimates (e.g. regression coefficient) AND variation (e.g. standard deviation) or associated estimates of uncertainty (e.g. confidence intervals)
☐	☒	For null hypothesis testing, the test statistic (e.g. *F*, *t*, *r*) with confidence intervals, effect sizes, degrees of freedom and *P* value noted<br>*Give P values as exact values whenever suitable.*
☒	☐	For Bayesian analysis, information on the choice of priors and Markov chain Monte Carlo settings
☒	☐	For hierarchical and complex designs, identification of the appropriate level for tests and full reporting of outcomes
☐	☒	Estimates of effect sizes (e.g. Cohen's *d*, Pearson's *r*), indicating how they were calculated

Our web collection on statistics for biologists contains articles on many of the points above.

Software and code

Policy information about availability of computer code

Data collection	Circadian rhythm of individual male flies was measured using the Drosophila Activity Monitoring (DAM) System (Trikinetics).<br>Immunofluorescence of brains were imaged using confocal microscopy (Leica SP8) with LAX software (Leica Application Suite X v3.3.0).<br>Quantification of genes expression were performed using Applied Biosystems (StepOne Software v2.3).<br>Western blot signals were detected using ECL (ABclonal, RM00021P) by Amersham ImageQuant 800 (GE Healthcare, Sweden).

Data analysis

For RNA-seq data analysis, Raw sequencing data (i.e. FASTQ files) of RNA-seq were processed and quality-controlled using Trim-galore v0.6.0 and FastQC v0.12.1. The remaining reads that passed all the filtering steps was counted as clean reads and all subsequent analyses were based on this. Hisat2 v2.2.1 was used to perform alignments to the Drosophila melanogaster (dm6) reference genome with default parameters. RNA-seq counts were counted using HTSeq-count v0.6.0 and differentially expressed genes (DEGs) were performed by R package DESeq2 v1.38.3 (FDR value < 0.05 and | log2 (fold changed) | > 0.5). Enrichment of Gene Ontology (GO) and Kyoto Encyclopedia of Genes and Genomes (KEGG) analyses were performed using the R package clusterProfiler and org.Dm.eg.db dictionary.
For CHIP-seq data analysis, Adaptor sequence trimming, mapping to the Drosophila melanogaster (dm6) reference genome using Bowtie2 v2.5.1 and PCR duplicate removal using Picard Tools (http://broadinstitute.github.io/picard/) were performed. Broad peaks were called using macs2 v2.2.7.1, annotated and analyzed by the R package CHIPseeker v1.34.1. Deeptools v 3.5.1 bamCoverage function, multiBigWigSummary function and R package pheatmap were used to generate ChIP-seq signal heatmaps and RNA-seq signal heatmaps represented the log2-ratio of fold change over gene body. Deeptools multiBigWigSummary function was used to compute average RPKM-normalized read count in peaks, and the Pearson correlation between different ChIP-seq targets were calculated by ggplot2 v3.4.1 in Rstudio. To identify H3K27me3/H3R17me2 co-modified genes, we first derived the H3K27me3 and H3R17me2 covered matrix. The H3K27me3/H3R17me2 co-modified genes were those genes with both H3K27me3 and H3R17me2 covered genes. We used k-means clustering to cluster different co-modified genes. Heatmap and cluster analysis were generated by R package ComplexHeatmap v2.14.0 and ClusterGVis v0.06, respectively.

For manuscripts utilizing custom algorithms or software that are central to the research but not yet described in published literature, software must be made available to editors and reviewers. We strongly encourage code deposition in a community repository (e.g. GitHub). See the Nature Portfolio guidelines for submitting code & software for further information.

Data

Policy information about availability of data

All manuscripts must include a data availability statement. This statement should provide the following information, where applicable:

- Accession codes, unique identifiers, or web links for publicly available datasets
- A description of any restrictions on data availability
- For clinical datasets or third party data, please ensure that the statement adheres to our policy

The RNA-seq data generated in this study have been deposited in the National Center for Biotechnology Information (NCBI) database under accession code PRJNA965887 (https://www.ncbi.nlm.nih.gov/sra/?term=PRJNA965887). The CHIP-seq data generated in this study have been deposited in the NCBI's Gene Expression Omnibus (GEO) database under accession code GSE235532 (https://www.ncbi.nlm.nih.gov/geo/query/acc.cgi?acc=GSE235532). Published gene expression datasets used in this study can be find from GEO database under accession code GSE153901 (https://www.ncbi.nlm.nih.gov/geo/query/acc.cgi?acc=GSE153901), GSE37032 (https://www.ncbi.nlm.nih.gov/geo/query/acc.cgi?acc=GSE37032), GSE59769 (https://www.ncbi.nlm.nih.gov/geo/query/acc.cgi?acc=GSE59769), GSE94922 (https://www.ncbi.nlm.nih.gov/geo/query/acc.cgi?acc=GSE94922). All other data needed to reproduce the results presented here are contained within the manuscript, figures, supplementary information and Zenodo database and are accessible to the public through the accession code: 10785489 (https://zenodo.org/records/10785489). Source data are provided with this paper.

Research involving human participants, their data, or biological material

Policy information about studies with human participants or human data. See also policy information about sex, gender (identity/presentation), and sexual orientation and race, ethnicity and racism.

Reporting on sex and gender	not applicable
Reporting on race, ethnicity, or other socially relevant groupings	not applicable
Population characteristics	not applicable
Recruitment	not applicable
Ethics oversight	not applicable

Note that full information on the approval of the study protocol must also be provided in the manuscript.

Field-specific reporting

Please select the one below that is the best fit for your research. If you are not sure, read the appropriate sections before making your selection.

☒ Life sciences ☐ Behavioural & social sciences ☐ Ecological, evolutionary & environmental sciences

For a reference copy of the document with all sections, see nature.com/documents/nr-reporting-summary-flat.pdf

Life sciences study design

All studies must disclose on these points even when the disclosure is negative.

Sample size

For circadian rhythm assays, sample size is over 32. For lifespan assays, sample size is 300. For other molecular biology assays, sample size is 20-30, which was indicated in methods descriptions.

Data exclusions	For circadian rhythm assays, data from the dead flies at the end of the assay were excluded.
Replication	Three biological repeats were applied in this study.
Randomization	Flies of the same genotype were randomly selected for each experiment.
Blinding	No blinding was done during this study. Normally, we put flies of the same genotype into one monitor. After the experiment, we analyze the data from the same monitor before we check the genotype.

Reporting for specific materials, systems and methods

We require information from authors about some types of materials, experimental systems and methods used in many studies. Here, indicate whether each material, system or method listed is relevant to your study. If you are not sure if a list item applies to your research, read the appropriate section before selecting a response.

Materials & experimental systems

n/a	Involved in the study
☐	☒ Antibodies
☐	☒ Eukaryotic cell lines
☒	☐ Palaeontology and archaeology
☐	☒ Animals and other organisms
☒	☐ Clinical data
☒	☐ Dual use research of concern
☒	☐ Plants

Methods

n/a	Involved in the study
☐	☒ ChIP-seq
☒	☐ Flow cytometry
☒	☐ MRI-based neuroimaging

Antibodies

Antibodies used	anti-Elav rat (DSHB, 7E8A10; 1:200)<br>anti-Repo mouse (DSHB, 8D12; 1:200)<br>Alexa FluorTM 568 (Thermo Fisher Scientific, A11004; 1:150)<br>Alexa FluorTM 647 (Thermo Fisher Scientific, A21247; 1:150)<br>anti-Histone H3 (asymmetric di methyl R17) (Abcam, ab8284; 1:500)<br>anti-Histone H3 (tri methyl K27) (Abcam, ab6002; 1:1000)<br>Histone H3 (EASYBIO, BE3015; 1:1000)<br>anti-AASS (Abconal, A24544; 1:50)<br>anti-V5 (Abconal, AE017; 1:1000)<br>anti-β-tubulin (Abconal, AC008; 1:1000)<br>HRP Goat Anti-Rabbit IgG (H+L) (ABconal, AS014; 1:1000)<br>HRP Goat Anti-Mouse IgG (H+L) (ABconal, AS003; 1:1000)
Validation	The validation of the primary antibodies was conducted previously, as these antibodies are widely used for immunofluorescence (IF), chromatin immunoprecipitation (ChIP), and Western blotting (WB), and their validation has been published before.

Eukaryotic cell lines

Policy information about cell lines and Sex and Gender in Research

Cell line source(s)	S2 cells were obtained from ATCC (ATCC, CRL-1963).
Authentication	cell line authentication was performed by the supplier.
Mycoplasma contamination	cell lines were negative for Mycoplasma contamination through observation of cell morphology.
Commonly misidentified lines<br>(See ICLAC register)	No commonly misidentified cell lines were used.

Animals and other research organisms

Policy information about studies involving animals; ARRIVE guidelines recommended for reporting animal research, and Sex and Gender in Research

Laboratory animals	3-day-old w1118, 3-day-old LKRSDHMB01942, 40-day-old w1118 and 40-day-old LKRSDHMB01942 fruit flies (Drosophila melanogaster) were used as the laboratory animals.
Wild animals	This study did not involve wild animals.

Reporting on sex	male
Field-collected samples	This study did not involve field-collected samples.
Ethics oversight	No ethical approval was required for the fly work.

Note that full information on the approval of the study protocol must also be provided in the manuscript.

Plants

Seed stocks	N/A
Novel plant genotypes	N/A
Authentication	N/A

ChIP-seq

Data deposition

☒ Confirm that both raw and final processed data have been deposited in a public database such as GEO.

☒ Confirm that you have deposited or provided access to graph files (e.g. BED files) for the called peaks.

Data access links<br>*May remain private before publication.*	https://www.ncbi.nlm.nih.gov/geo/query/acc.cgi?acc=GSE235532
Files in database submission	The database includes raw data (fastq files) and processed data (bigWig and BED files) for all NGS studies.
Genome browser session<br>(e.g. UCSC)	No longer applicable

Methodology

Replicates	Two biological repeats were applied in this study.
Sequencing depth	6G
Antibodies	anti-Histone H3 (asymmetric di methyl R17) (Abcam, ab8284)<br>anti-Histone H3 (tri methyl K27) (Abcam, ab6002)
Peak calling parameters	macs2 callpeak -c input.bam -t H3K27me3.bam -p 0.01 -f BAMPE -g dm -B --broad --min-length 500 -m 3 50 --outdir peak_nomodle -n H3K27me3 &<br>macs2 callpeak -c input.bam -t H3R17me2.bam -p 0.01 -f BAMPE -g dm -B --broad --min-length 500 -m 3 50 --outdir peak_nomodle -n H3R17me2
Data quality	Quality of raw data was evaluated by FastQC v0.12.1. Peaks were called in MACS2 (v2.2.7.1) using p-value of 0.01 for ChIP-seq. The peaks called were further validated by visualizing the peak postion files and bigWig files for all samples including the Input control in a genome browser. In addition, we also validated the reliability of CHIP-seq data through CHIP-qPCR.
Software	Trim-galore (v0.6.0) was used to trim CHIP-seq reads.<br>Bowtie2 (v2.5.1) was used to align reads to genome.<br>SAMtools (v1.19) was used for BAM file processing and merging of replicate BAM files.<br>PicardTools (v2.8) was used for PCR duplicates removal.<br>MACS2 (v2.2.7.1) was used for peaking calling.<br>Deeptools (v3.5.1) was used to generate bigWig track coverage files.<br>CHIPseeker (v1.36.0) was used to annotate and analyze Broad peaks.<br>Complex Heatmap (v 2.14.0) and ClusterGVis v0.1.0 was used to generate heatmap and cluster analysis.
