# Supplementary figures and images for: LKRSDH-dependent histone modifications of insulin-like peptide sites contribute to age-related circadian rhythm changes
Source: Nat Commun. 2024 Apr 18;15:3336. doi: 10.1038/s41467-024-47740-4 (PMC11026460; doi:10.1038/s41467-024-47740-4)

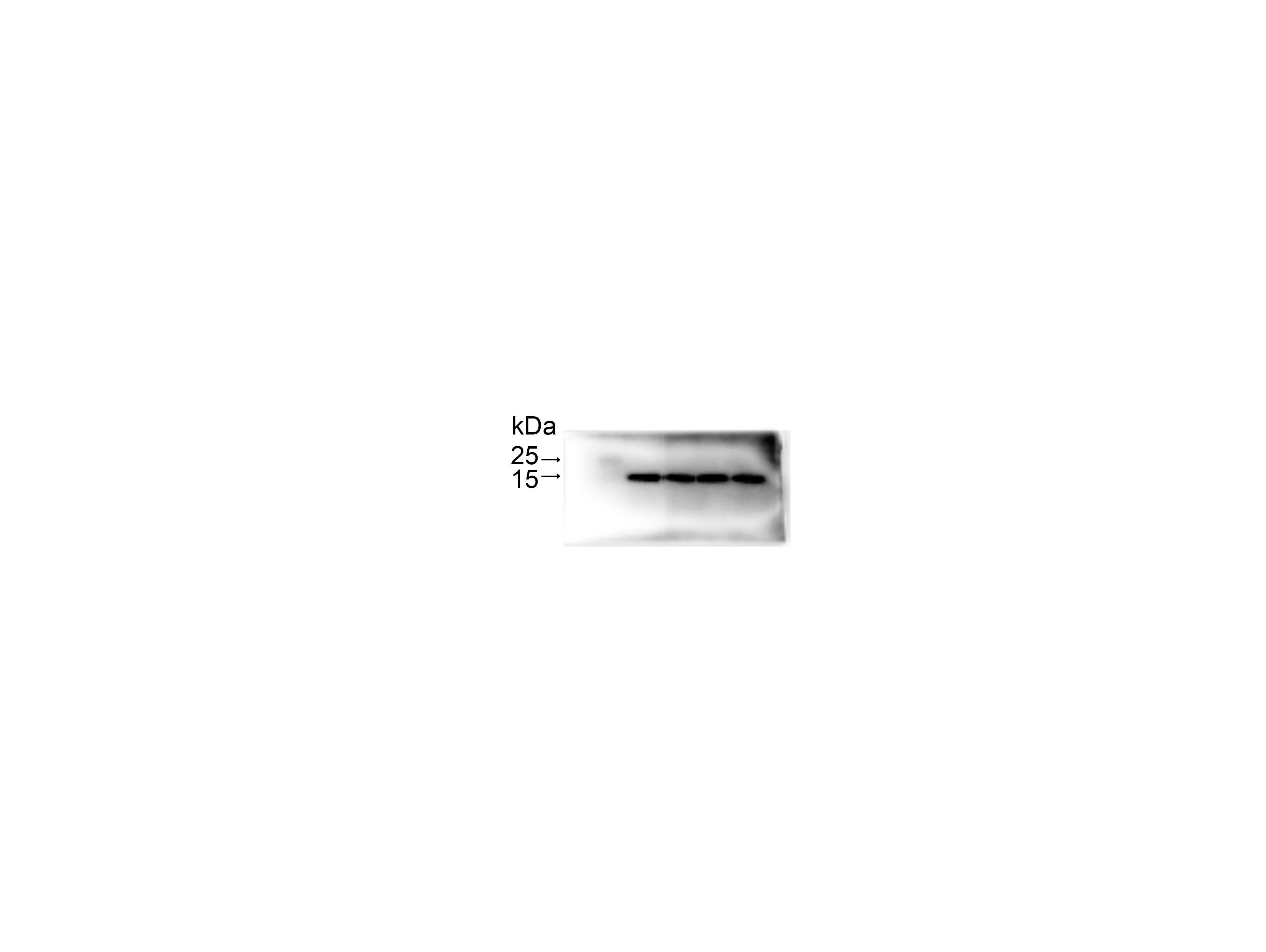

Supplement: Supplementary file 8 — Source data [file 41467_2024_47740_MOESM8_ESM.zip › Source Data/Uncropped blots for Fig.6a/Replicate 1 main text/anti-H3.tif]

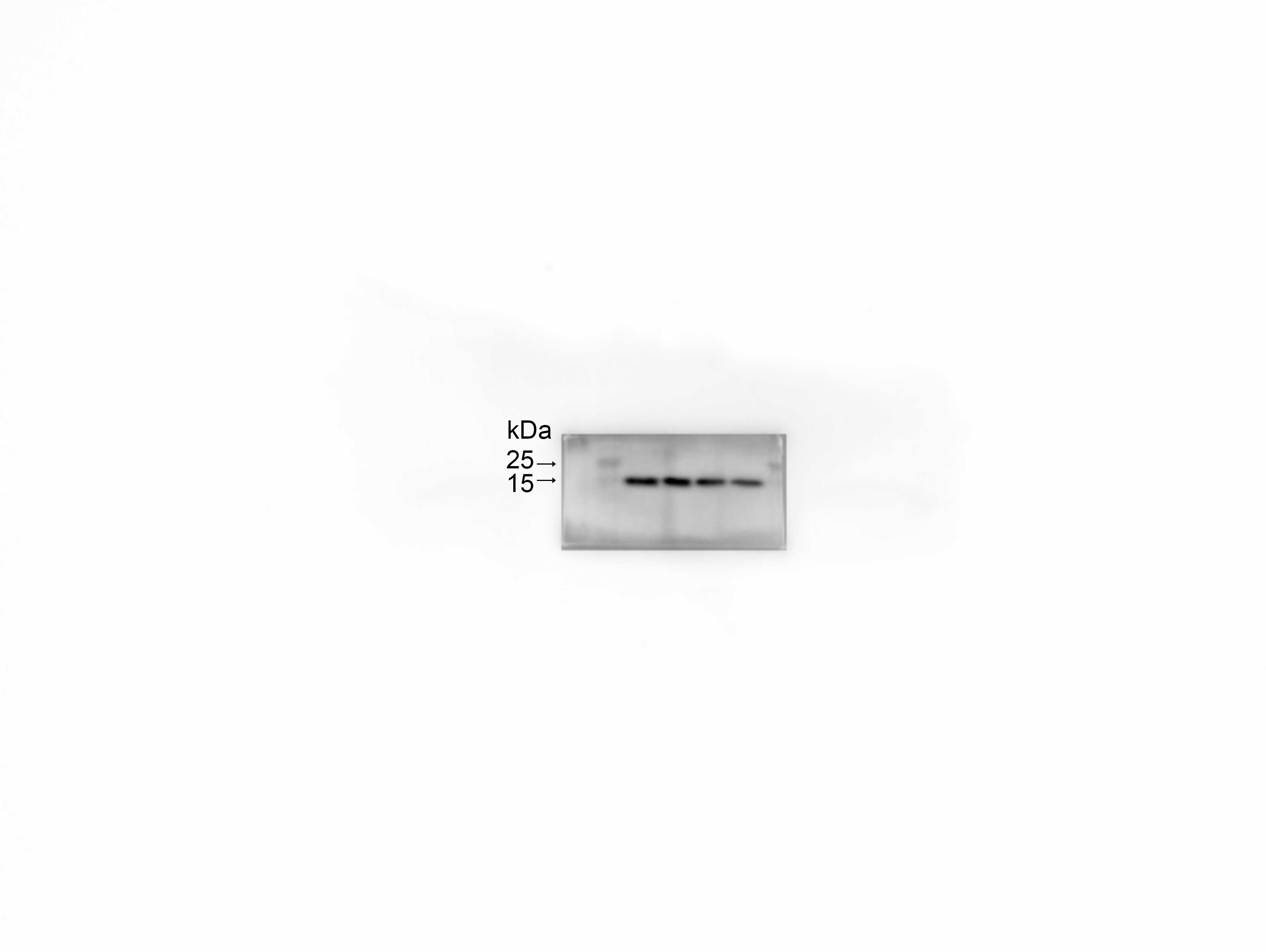

Supplement: Supplementary file 8 — Source data [file 41467_2024_47740_MOESM8_ESM.zip › Source Data/Uncropped blots for Fig.6a/Replicate 1 main text/anti-H3R17me2.tif]

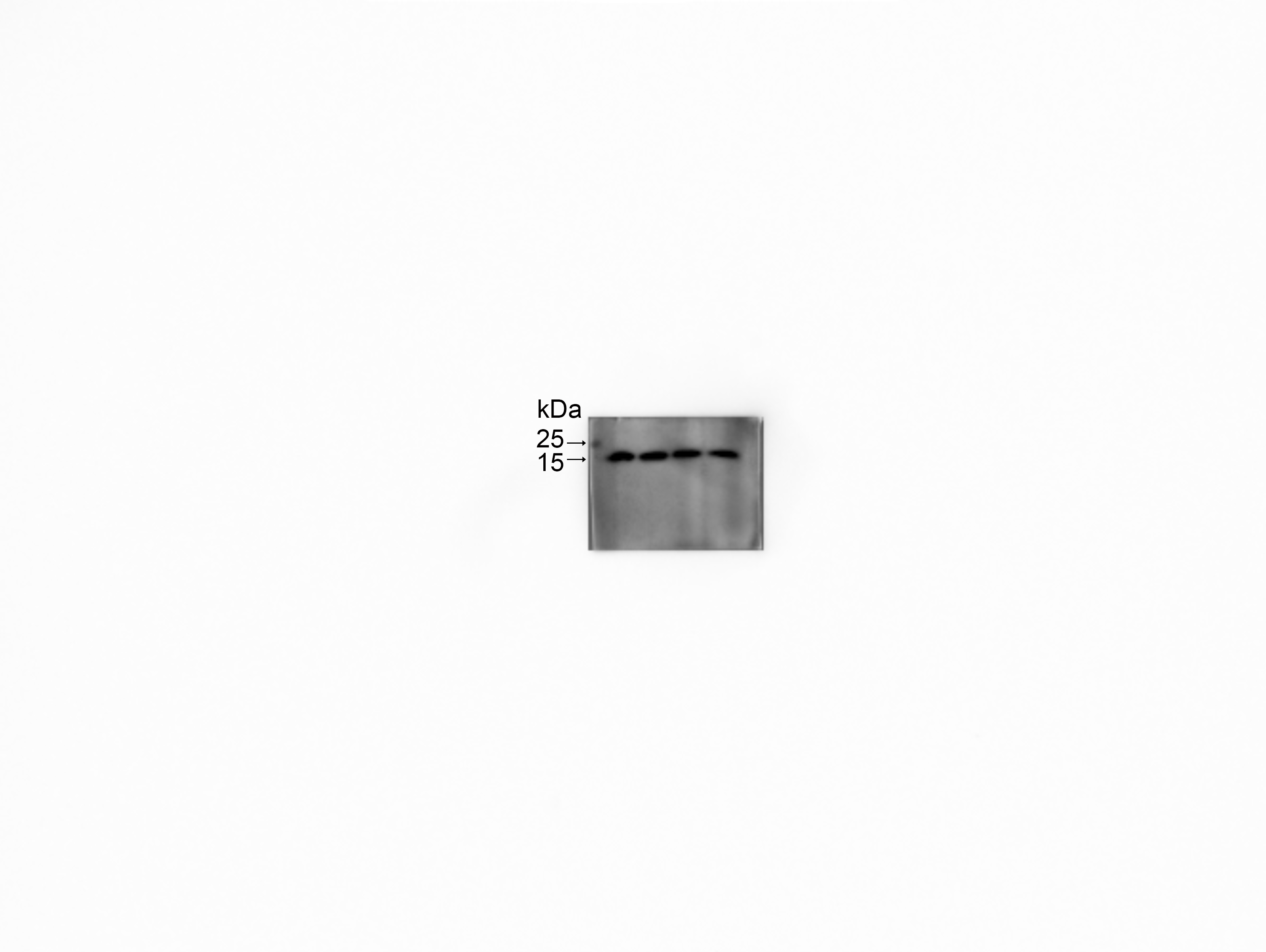

Supplement: Supplementary file 8 — Source data [file 41467_2024_47740_MOESM8_ESM.zip › Source Data/Uncropped blots for Fig.6a/Replicate 2/anti-H3.tif]

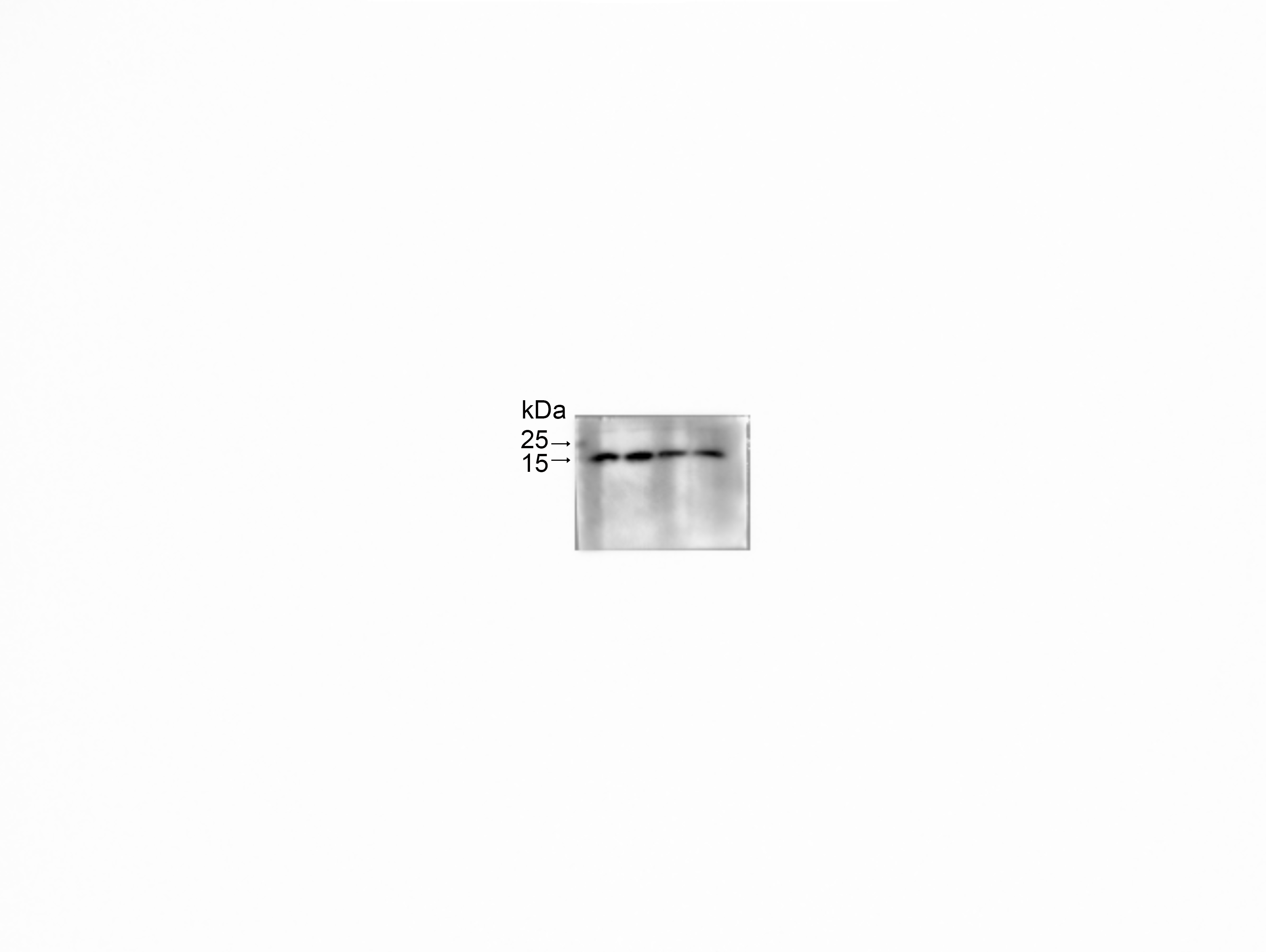

Supplement: Supplementary file 8 — Source data [file 41467_2024_47740_MOESM8_ESM.zip › Source Data/Uncropped blots for Fig.6a/Replicate 2/anti-H3R17me2.tif]

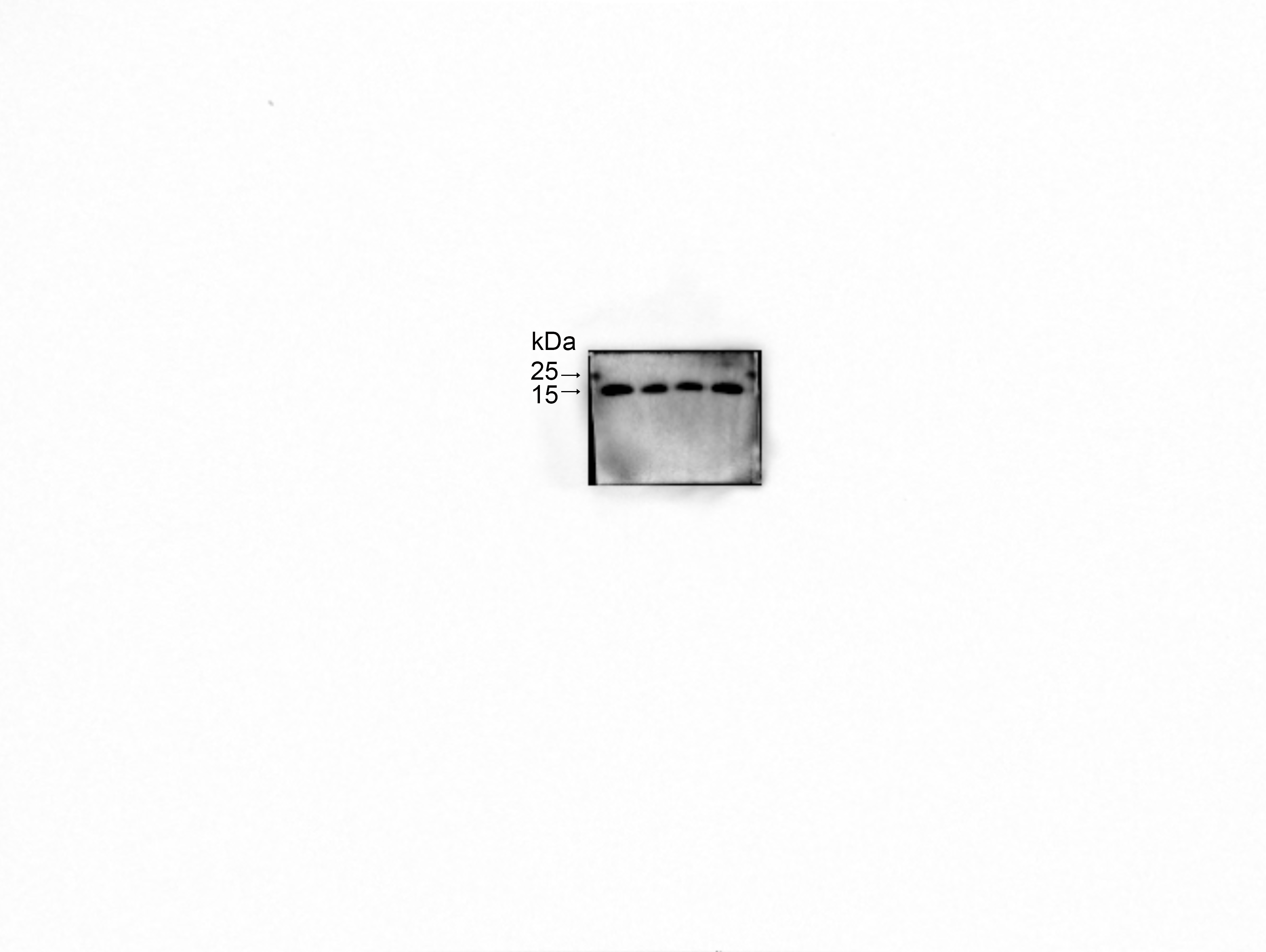

Supplement: Supplementary file 8 — Source data [file 41467_2024_47740_MOESM8_ESM.zip › Source Data/Uncropped blots for Fig.6a/Replicate 3/anti-H3.tif]

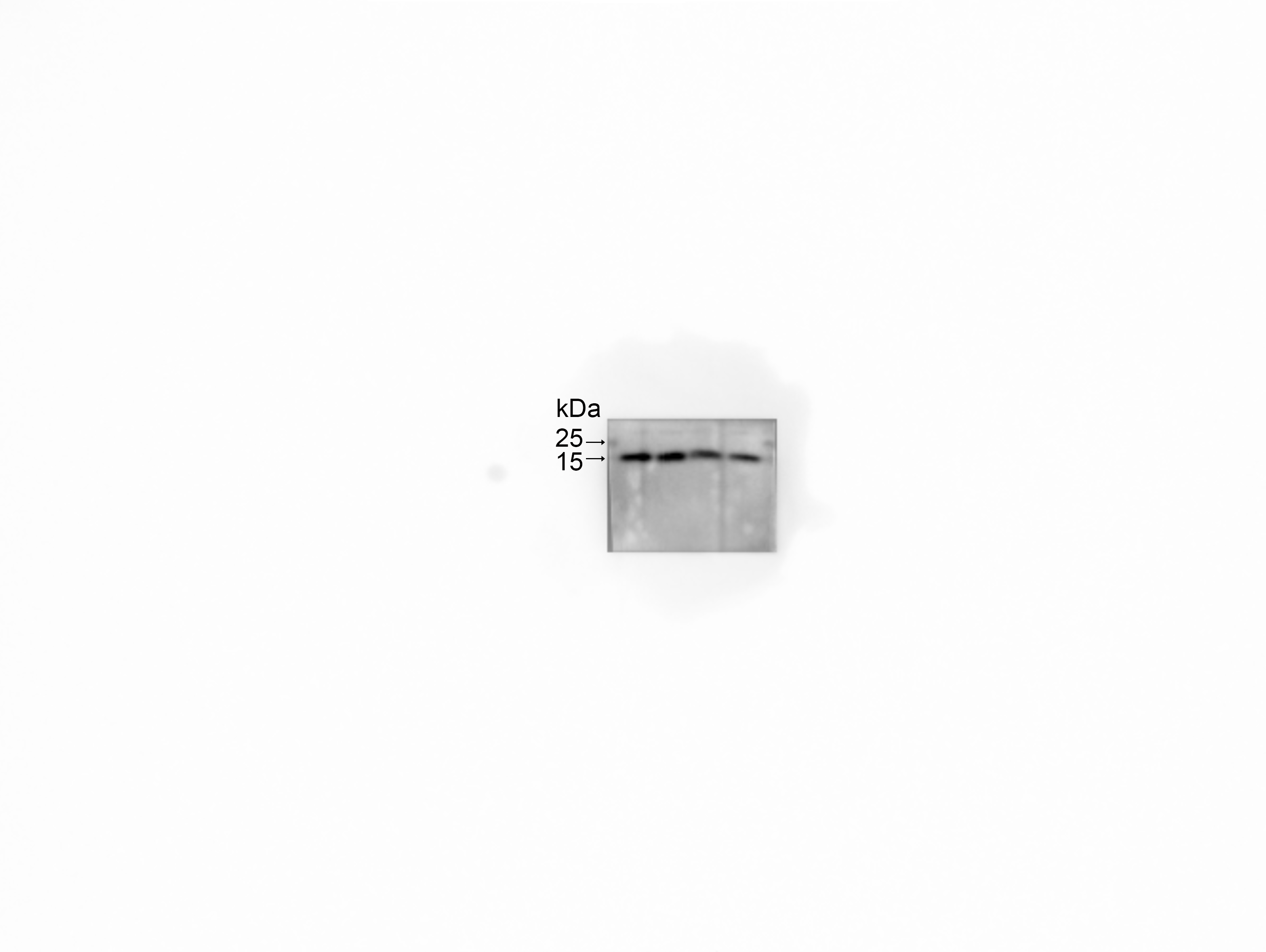

Supplement: Supplementary file 8 — Source data [file 41467_2024_47740_MOESM8_ESM.zip › Source Data/Uncropped blots for Fig.6a/Replicate 3/anti-H3R17me2.tif]

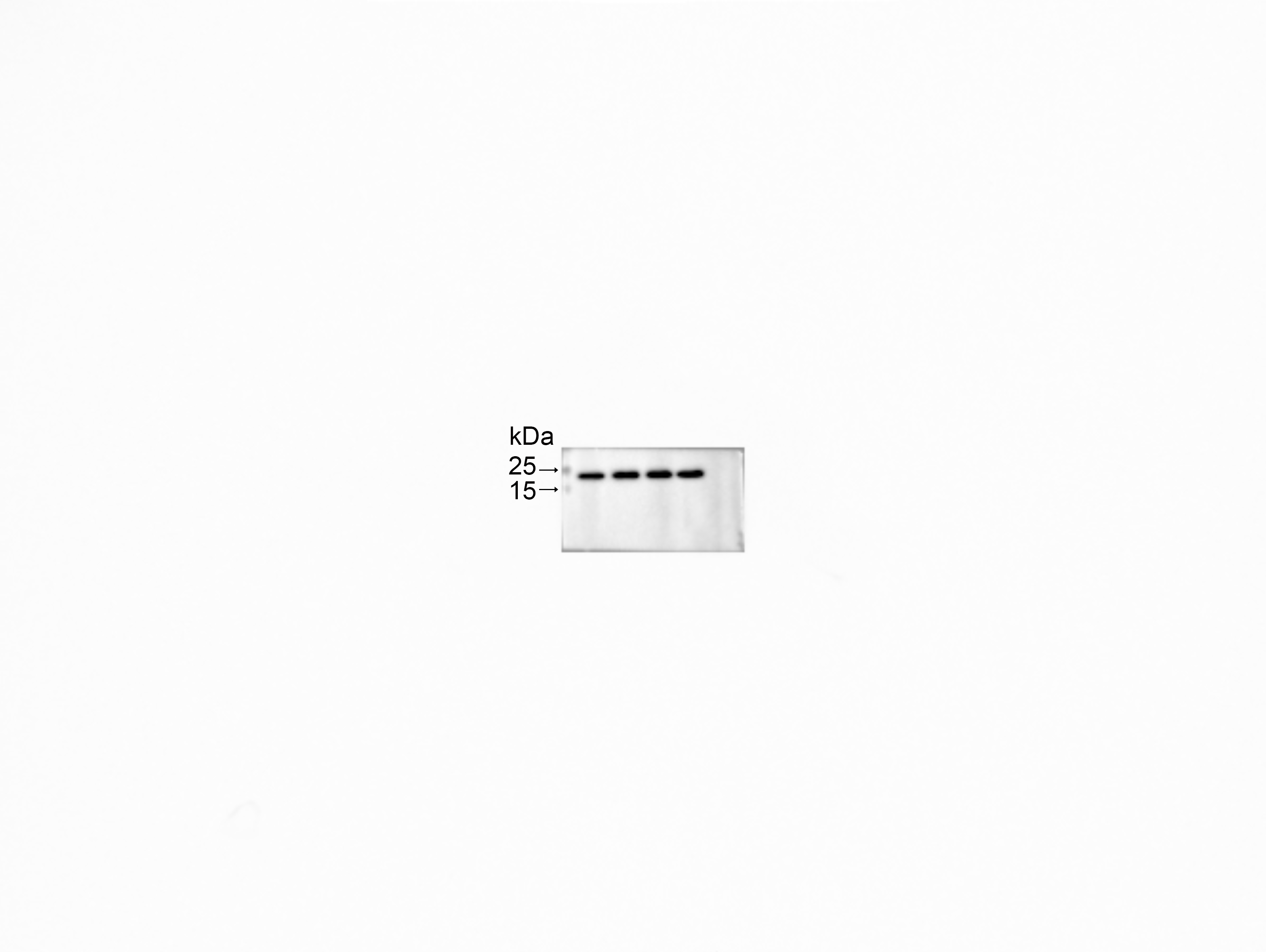

Supplement: Supplementary file 8 — Source data [file 41467_2024_47740_MOESM8_ESM.zip › Source Data/Uncropped blots for Fig.6b/Replicate 1 main text/anti-H3.tif]

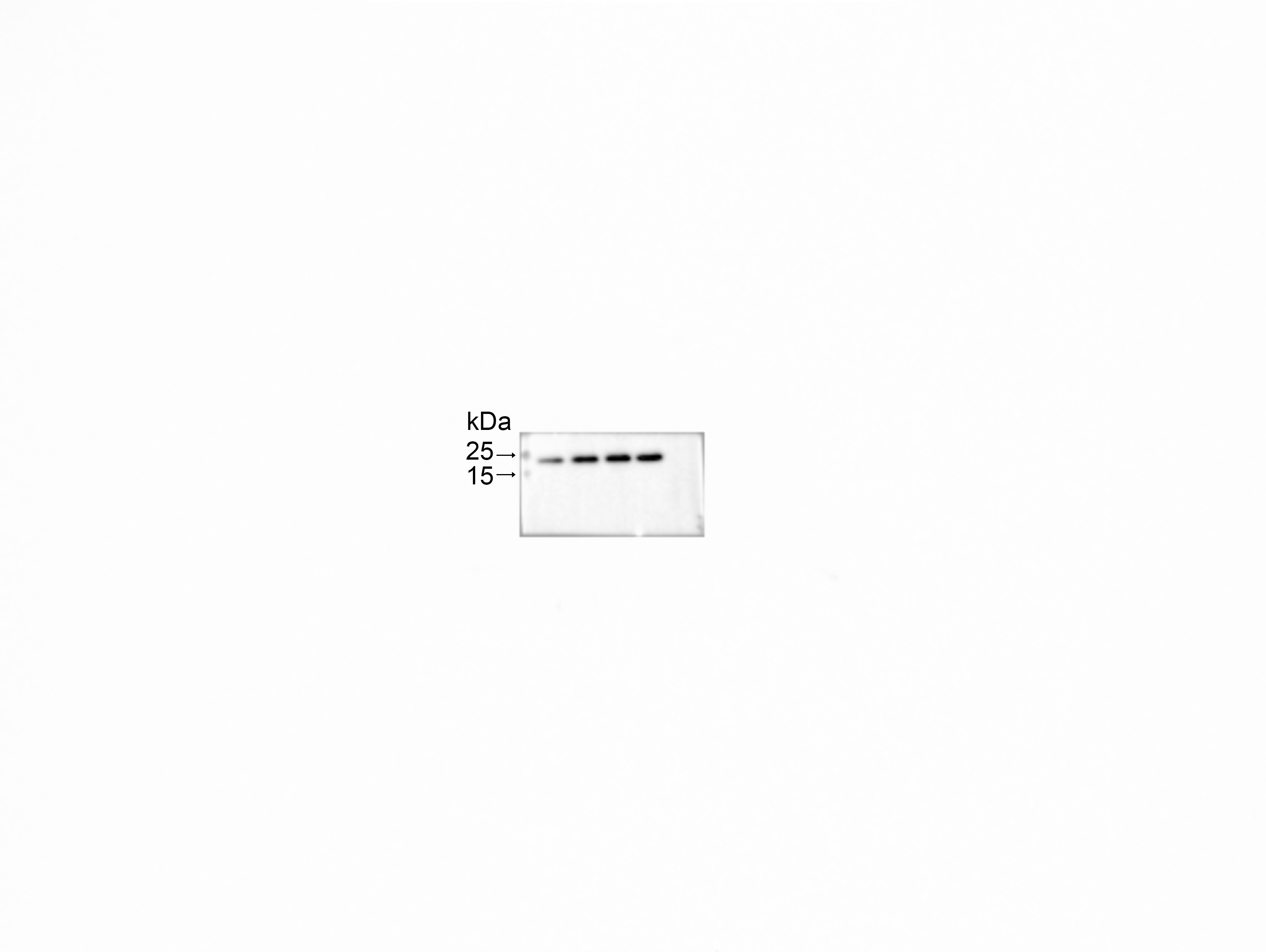

Supplement: Supplementary file 8 — Source data [file 41467_2024_47740_MOESM8_ESM.zip › Source Data/Uncropped blots for Fig.6b/Replicate 1 main text/anti-H3K27me3.tif]

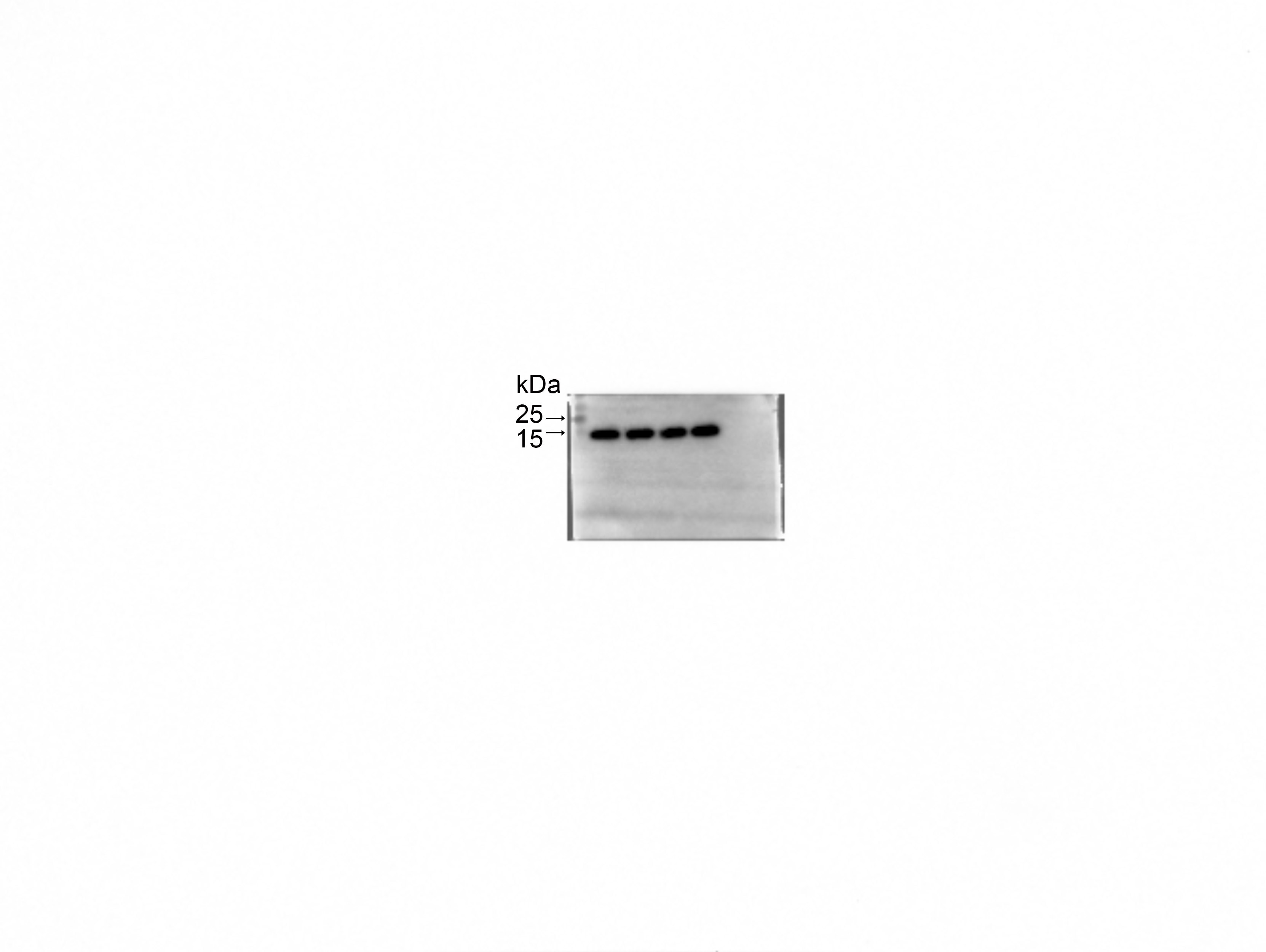

Supplement: Supplementary file 8 — Source data [file 41467_2024_47740_MOESM8_ESM.zip › Source Data/Uncropped blots for Fig.6b/Replicate 2/anti-H3.tif]

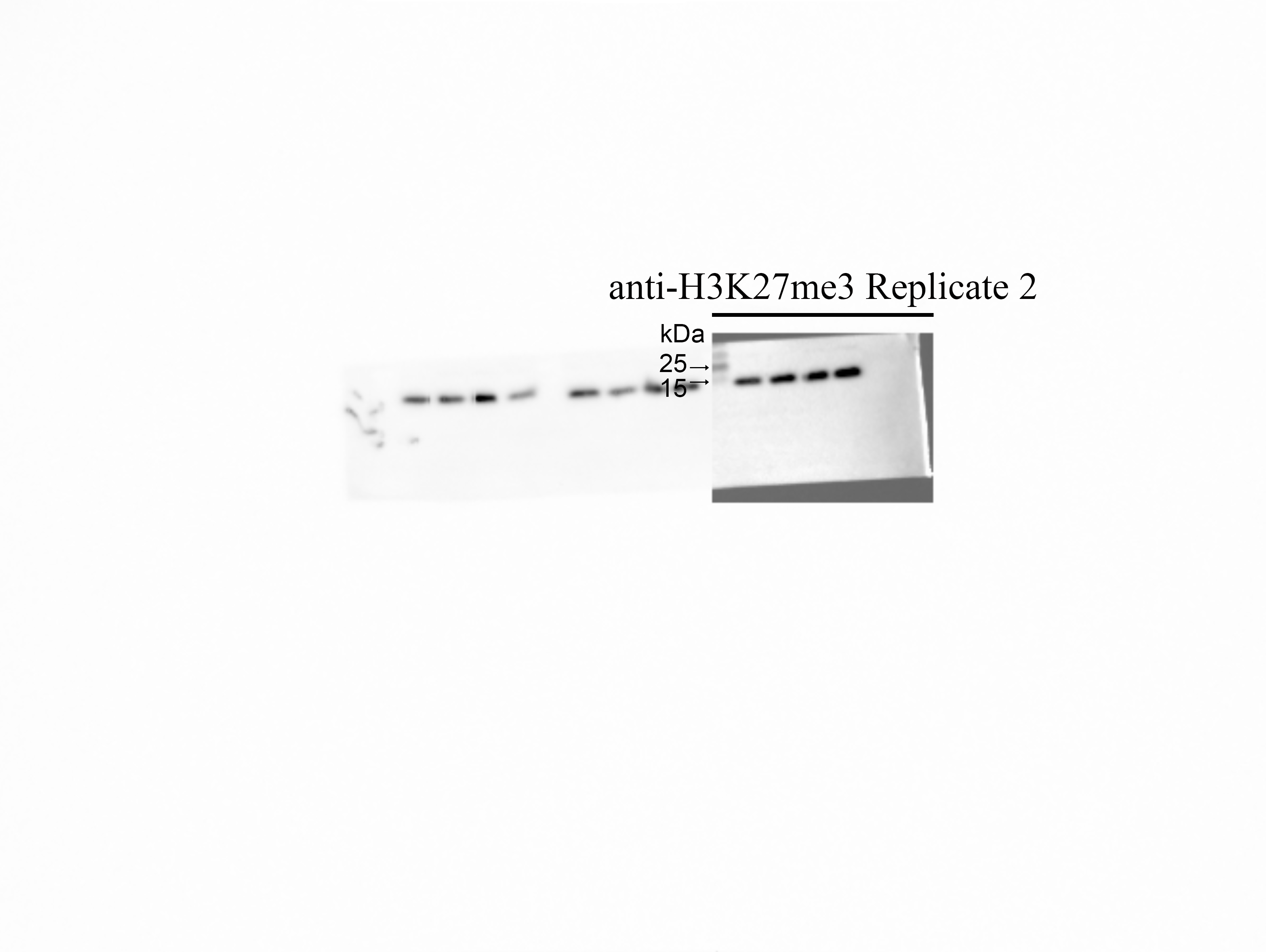

Supplement: Supplementary file 8 — Source data [file 41467_2024_47740_MOESM8_ESM.zip › Source Data/Uncropped blots for Fig.6b/Replicate 2/anti-H3K27me3.tif]

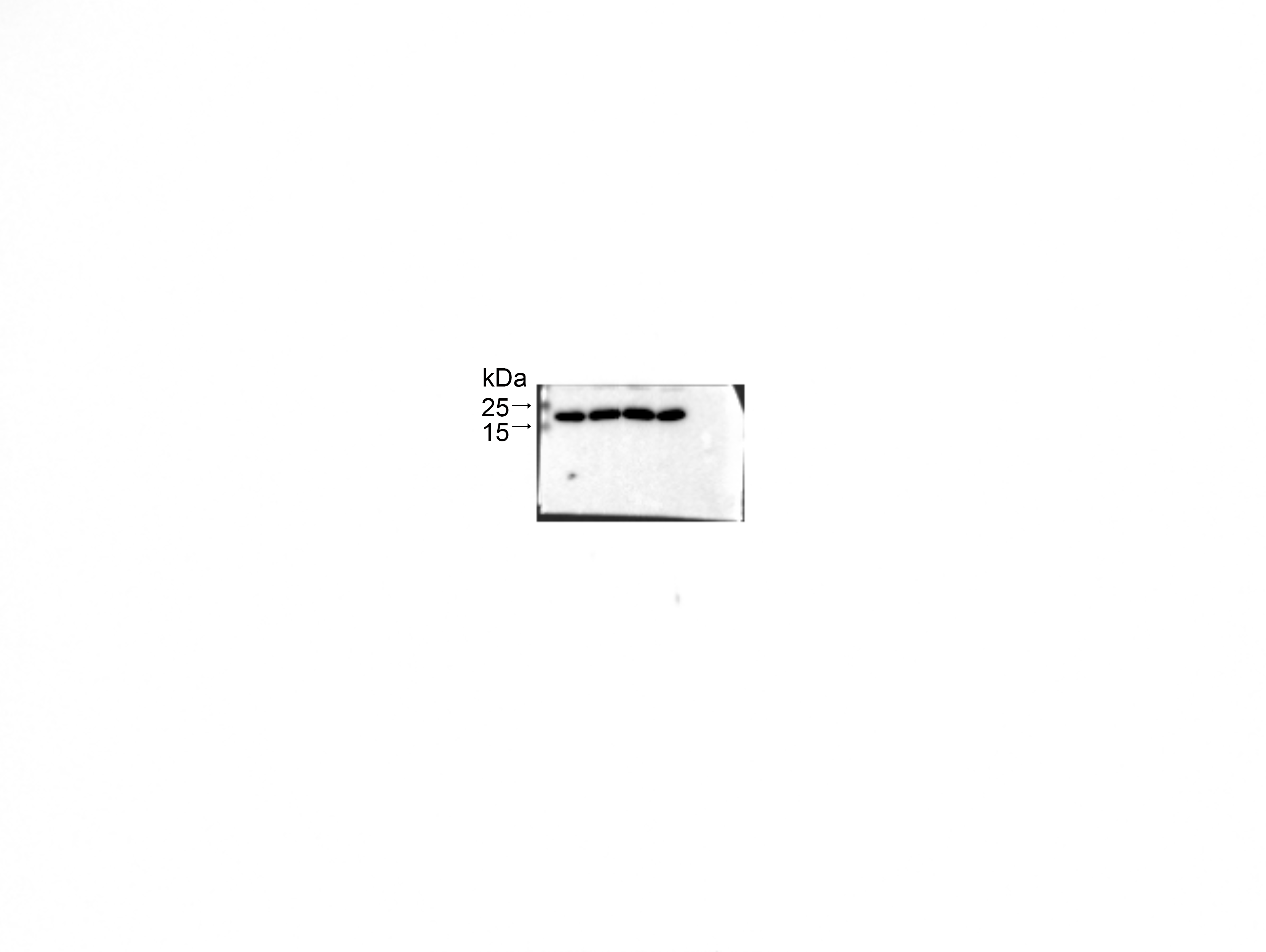

Supplement: Supplementary file 8 — Source data [file 41467_2024_47740_MOESM8_ESM.zip › Source Data/Uncropped blots for Fig.6b/Replicate 3/anti-H3.tif]

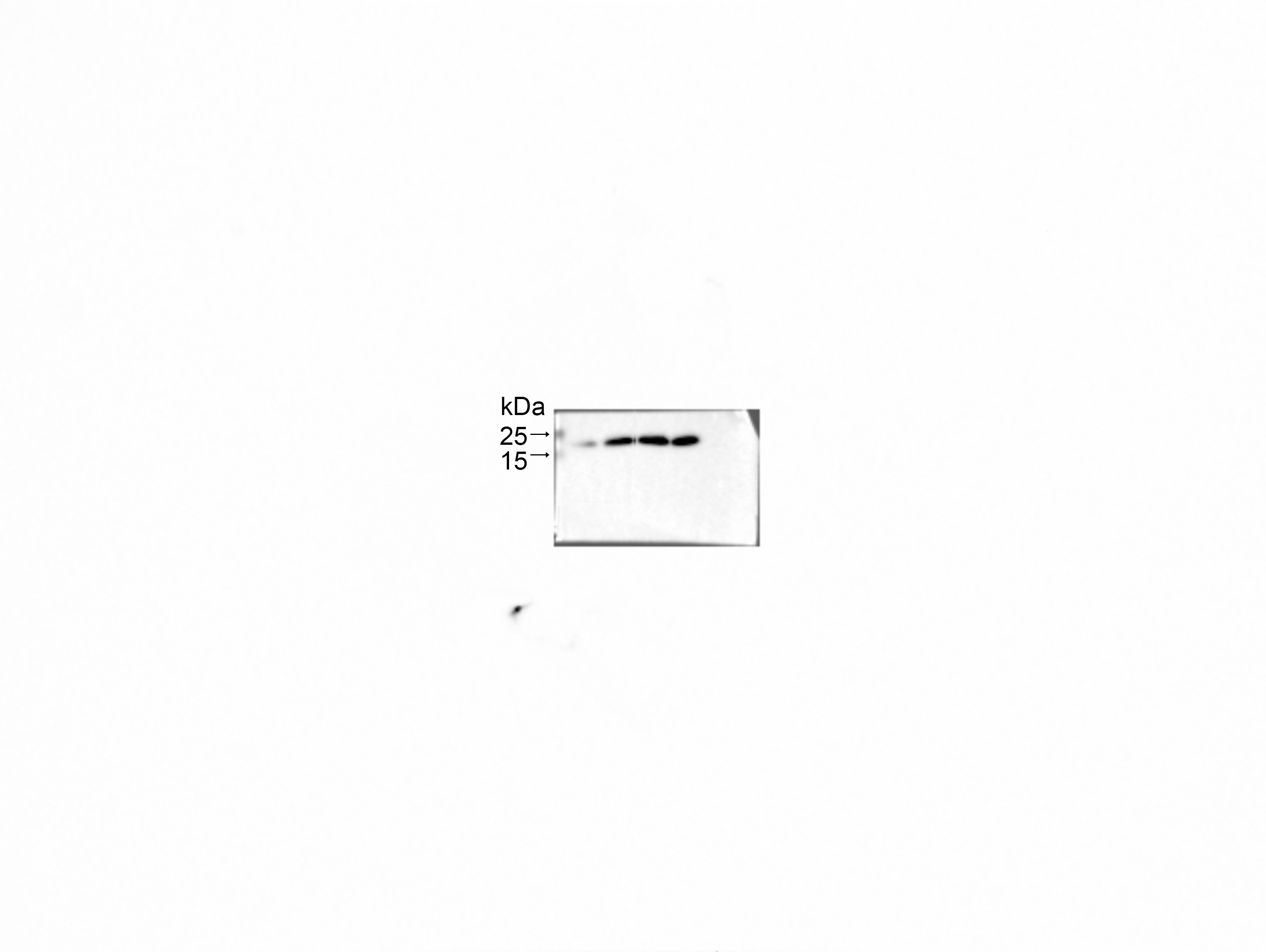

Supplement: Supplementary file 8 — Source data [file 41467_2024_47740_MOESM8_ESM.zip › Source Data/Uncropped blots for Fig.6b/Replicate 3/anti-H3K27me3.tif]

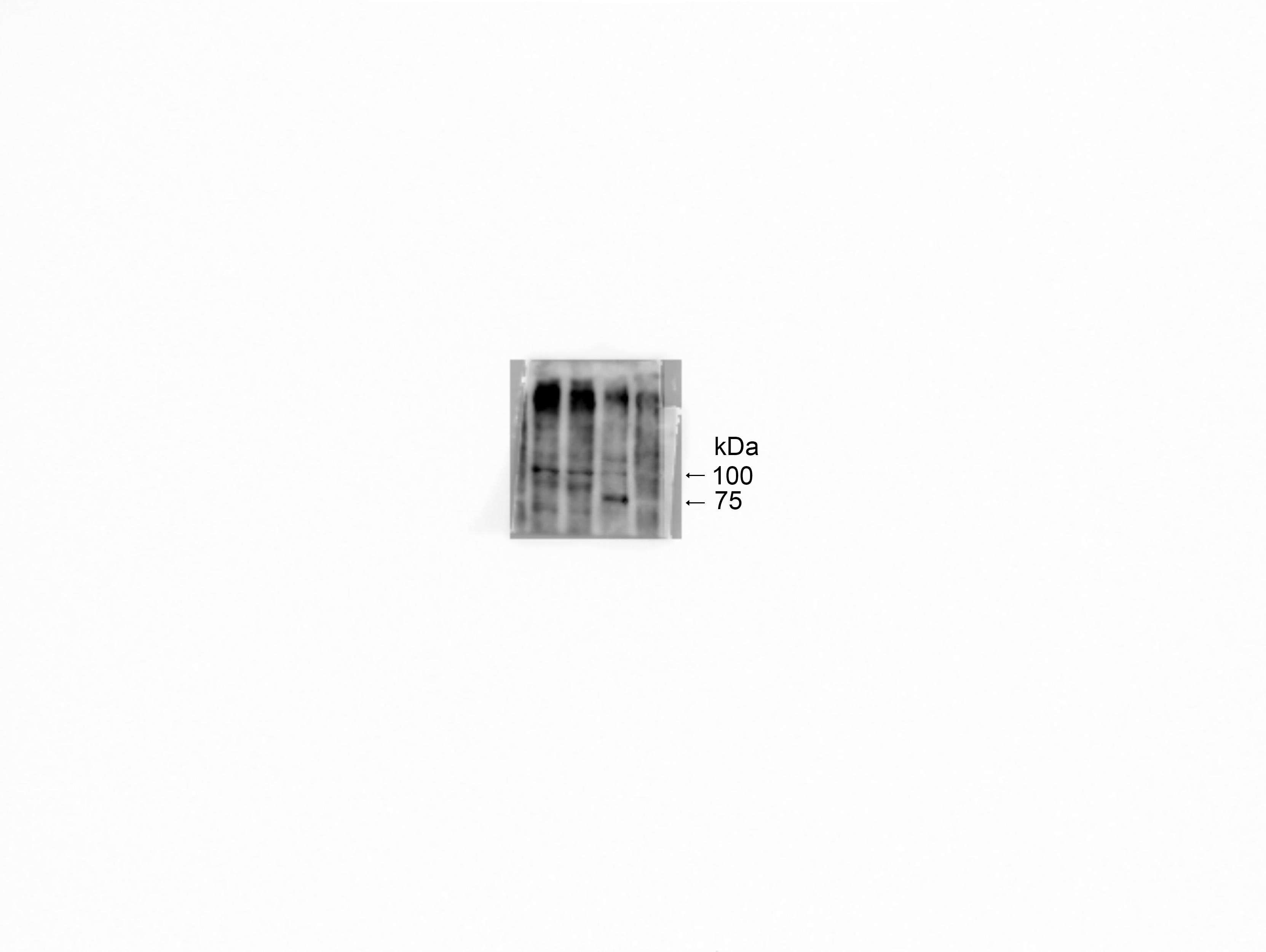

Supplement: Supplementary file 8 — Source data [file 41467_2024_47740_MOESM8_ESM.zip › Source Data/Uncropped blots for Supplementary Fig.1j/Replicate 1 main text/anti-LKRSDH.tif]

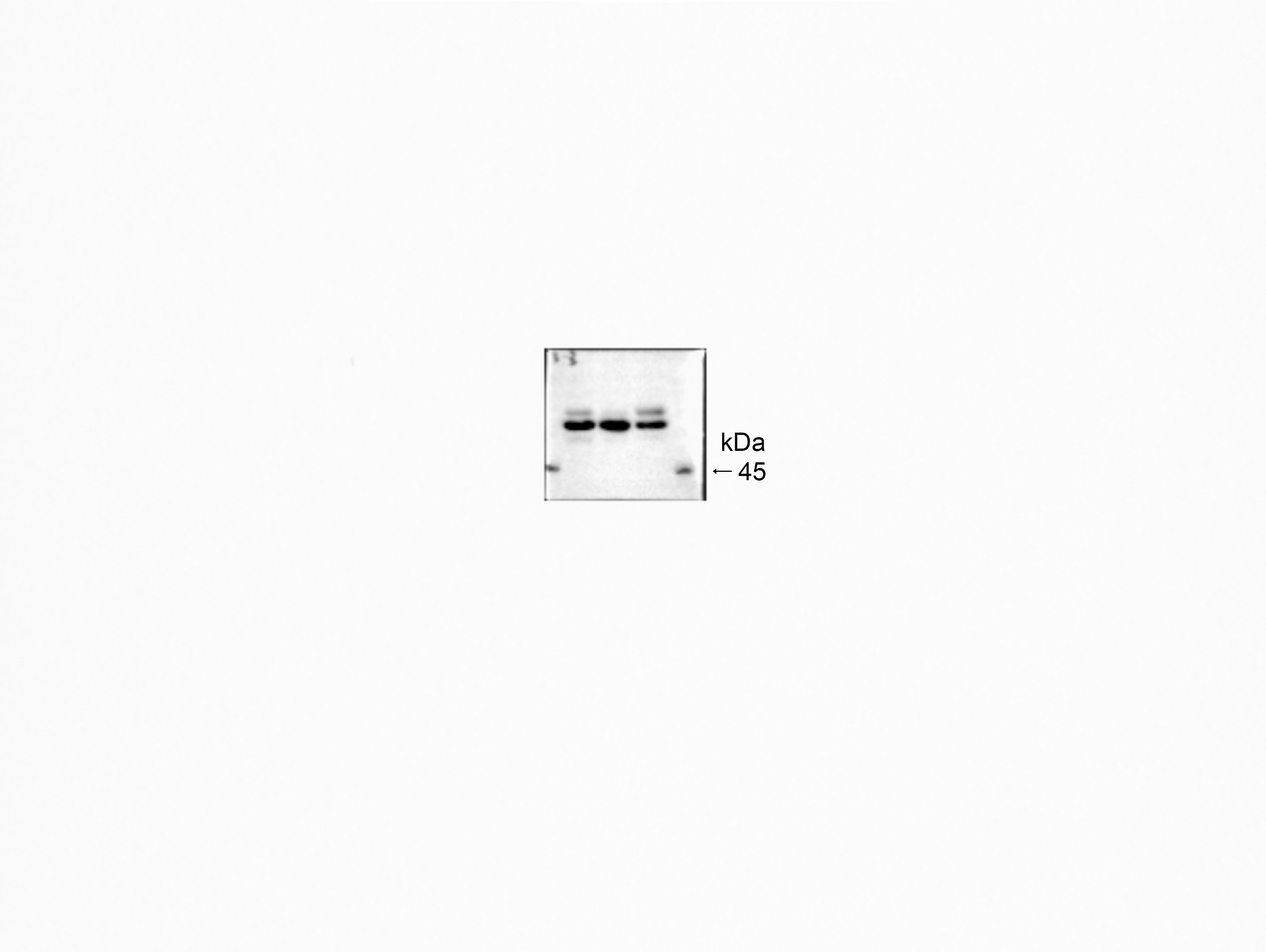

Supplement: Supplementary file 8 — Source data [file 41467_2024_47740_MOESM8_ESM.zip › Source Data/Uncropped blots for Supplementary Fig.1j/Replicate 1 main text/anti-β-Tubulin.tif]

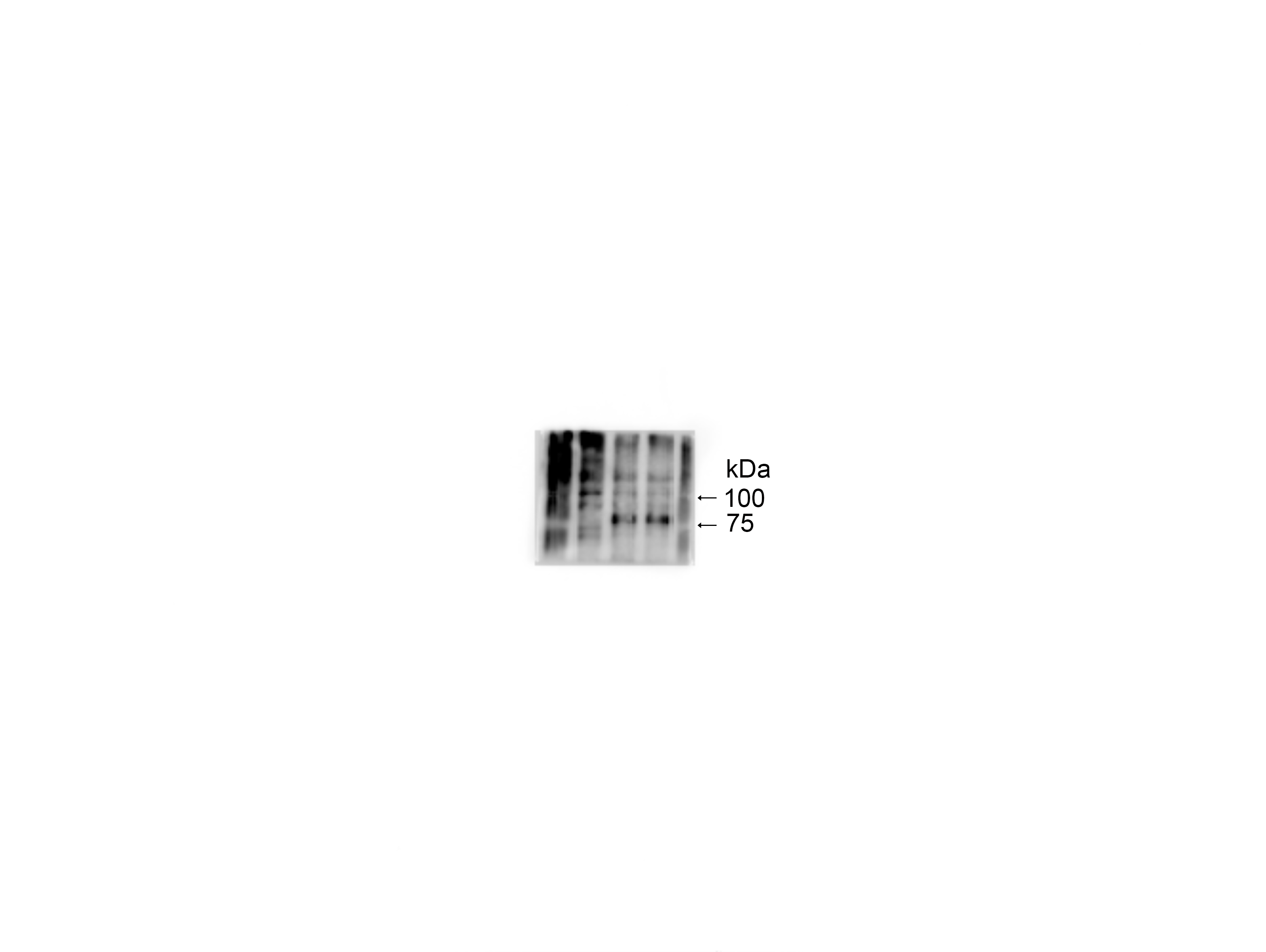

Supplement: Supplementary file 8 — Source data [file 41467_2024_47740_MOESM8_ESM.zip › Source Data/Uncropped blots for Supplementary Fig.1j/Replicate 2/anti-LKRSDH.tif]

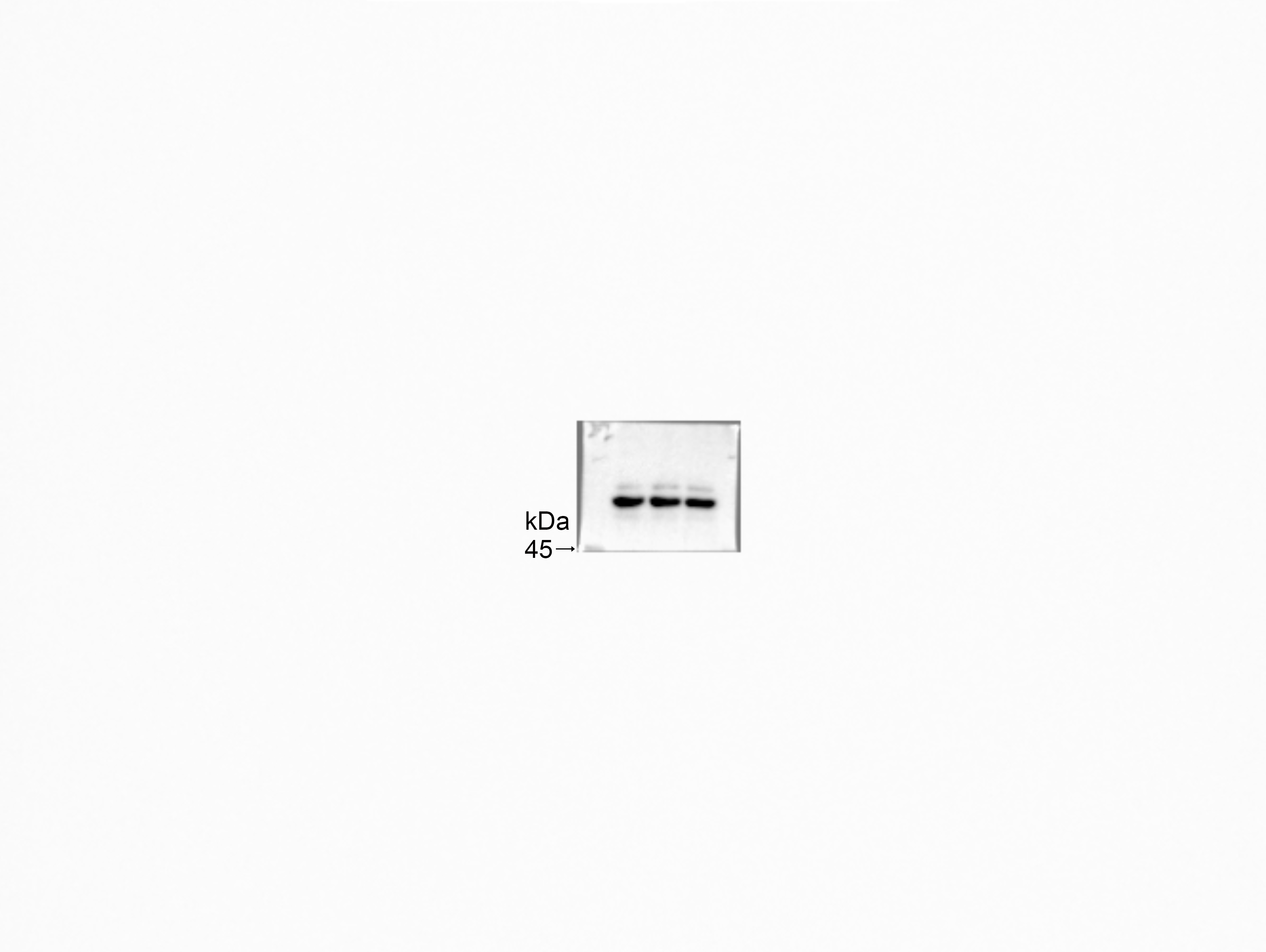

Supplement: Supplementary file 8 — Source data [file 41467_2024_47740_MOESM8_ESM.zip › Source Data/Uncropped blots for Supplementary Fig.1j/Replicate 2/anti-β-Tubulin.tif]

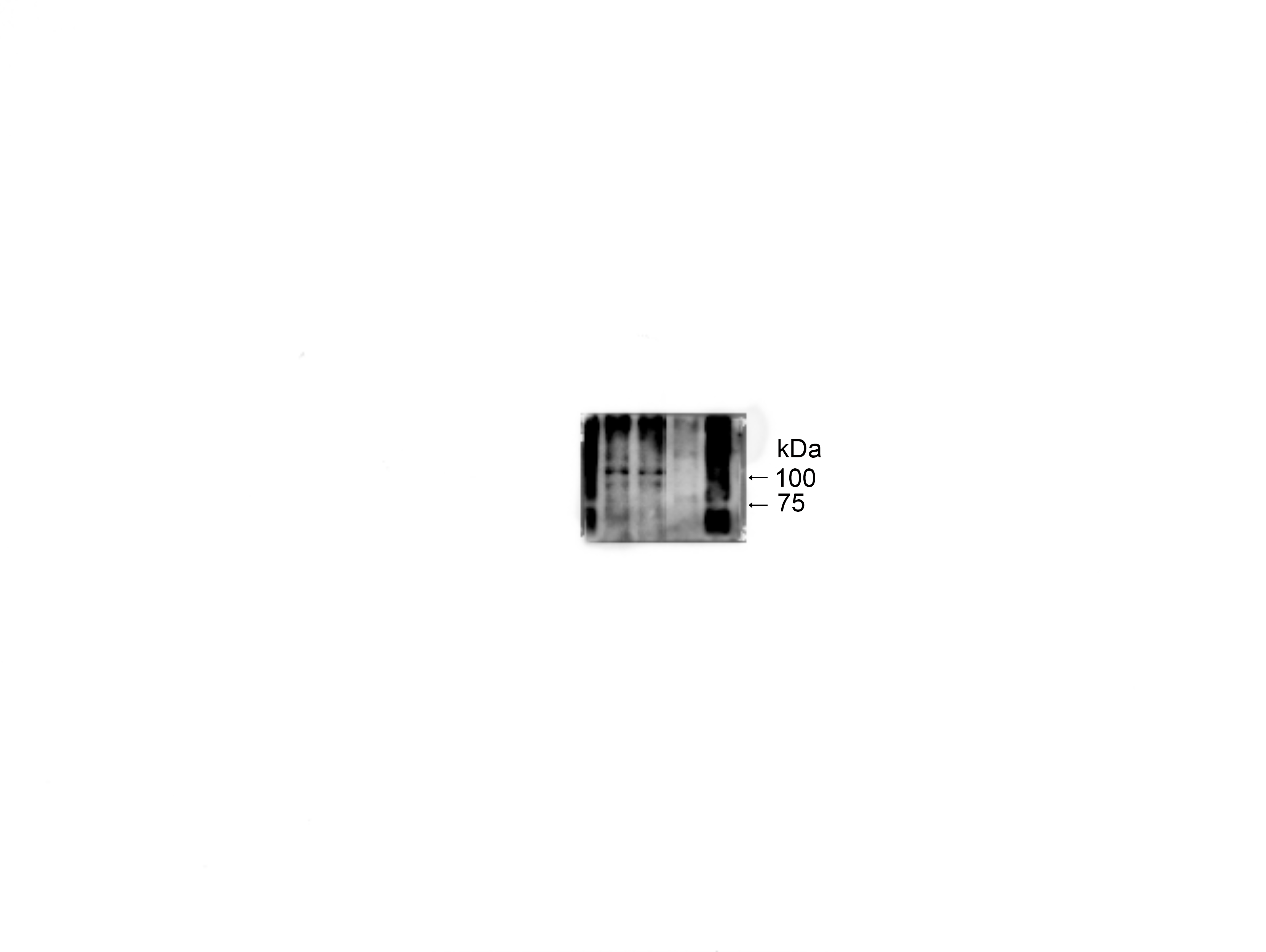

Supplement: Supplementary file 8 — Source data [file 41467_2024_47740_MOESM8_ESM.zip › Source Data/Uncropped blots for Supplementary Fig.1j/Replicate 3/anti-LKRSDH.tif]

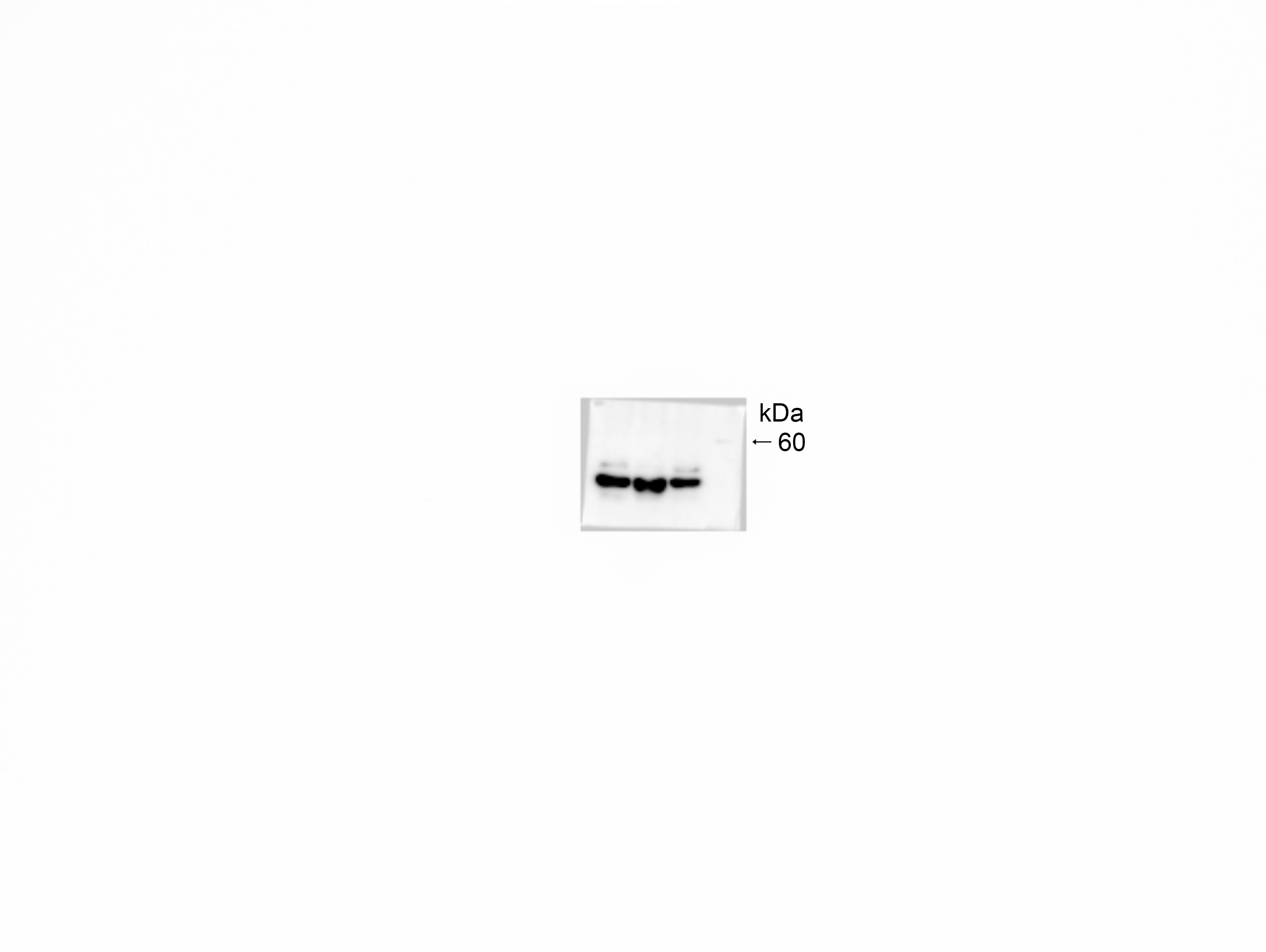

Supplement: Supplementary file 8 — Source data [file 41467_2024_47740_MOESM8_ESM.zip › Source Data/Uncropped blots for Supplementary Fig.1j/Replicate 3/anti-β-Tubulin.tif]

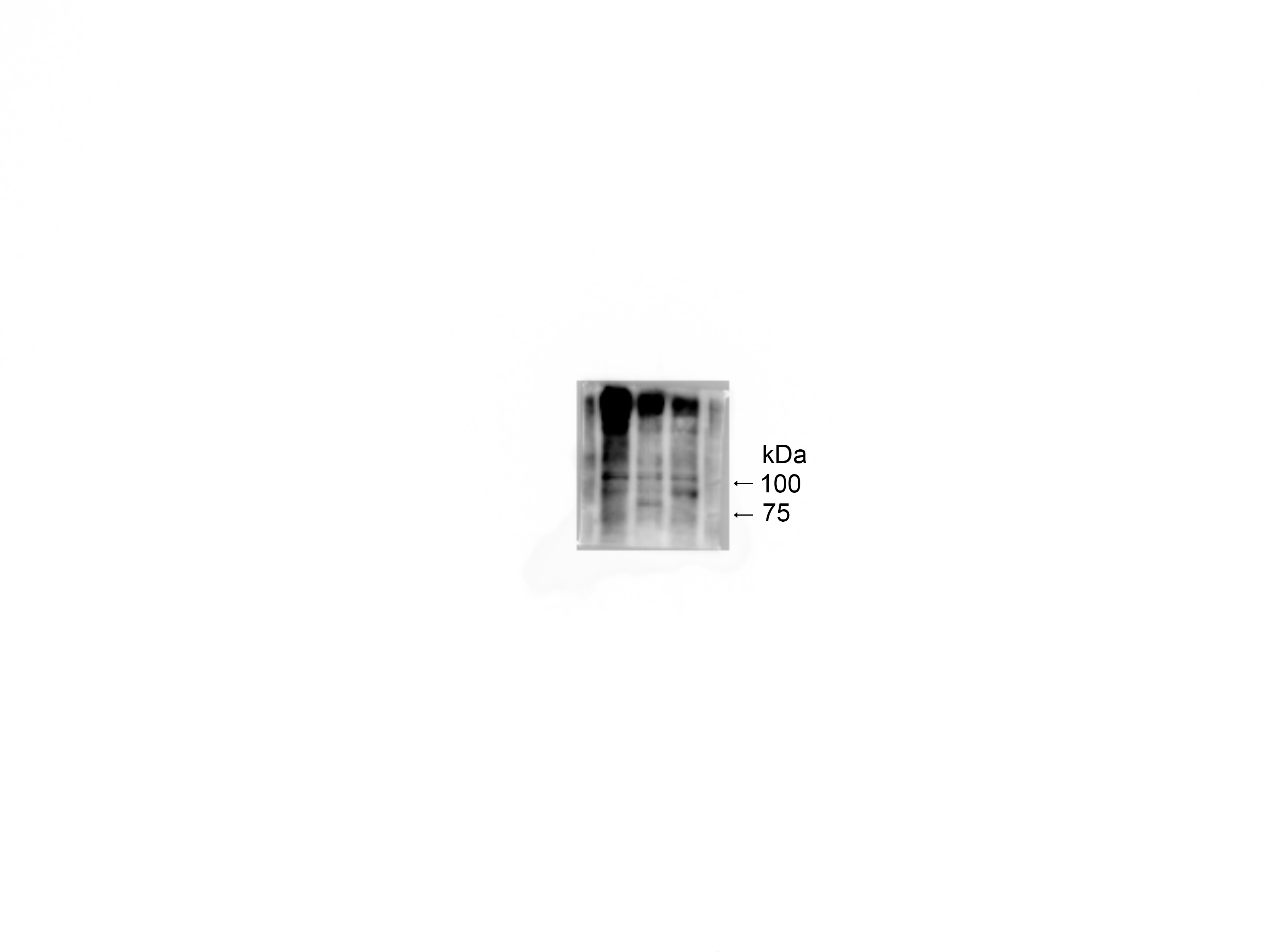

Supplement: Supplementary file 8 — Source data [file 41467_2024_47740_MOESM8_ESM.zip › Source Data/Uncropped blots for Supplementary Fig.1j/Replicate 4/anti-LKRSDH.tif]

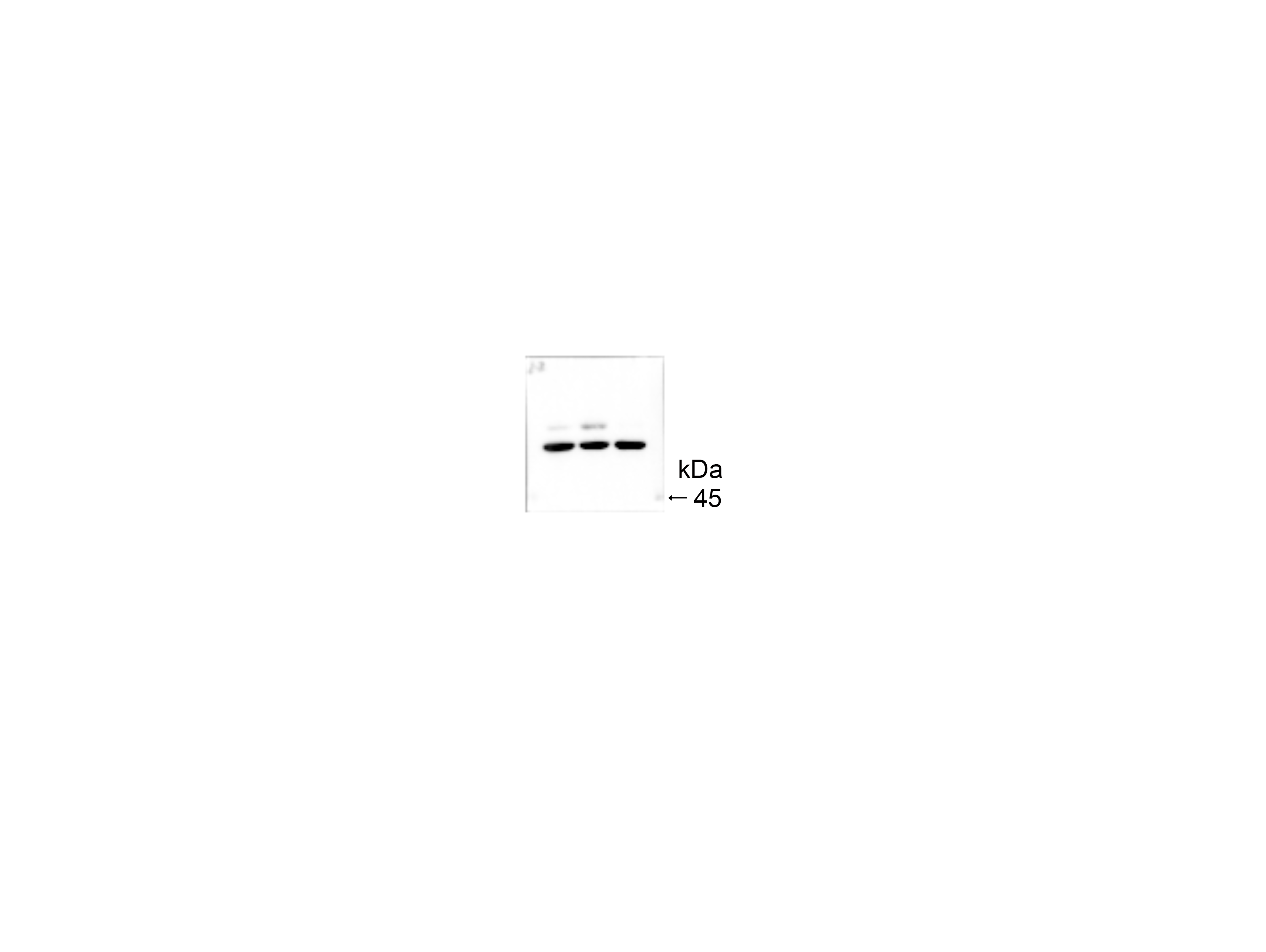

Supplement: Supplementary file 8 — Source data [file 41467_2024_47740_MOESM8_ESM.zip › Source Data/Uncropped blots for Supplementary Fig.1j/Replicate 4/anti-β-Tubulin.tif]

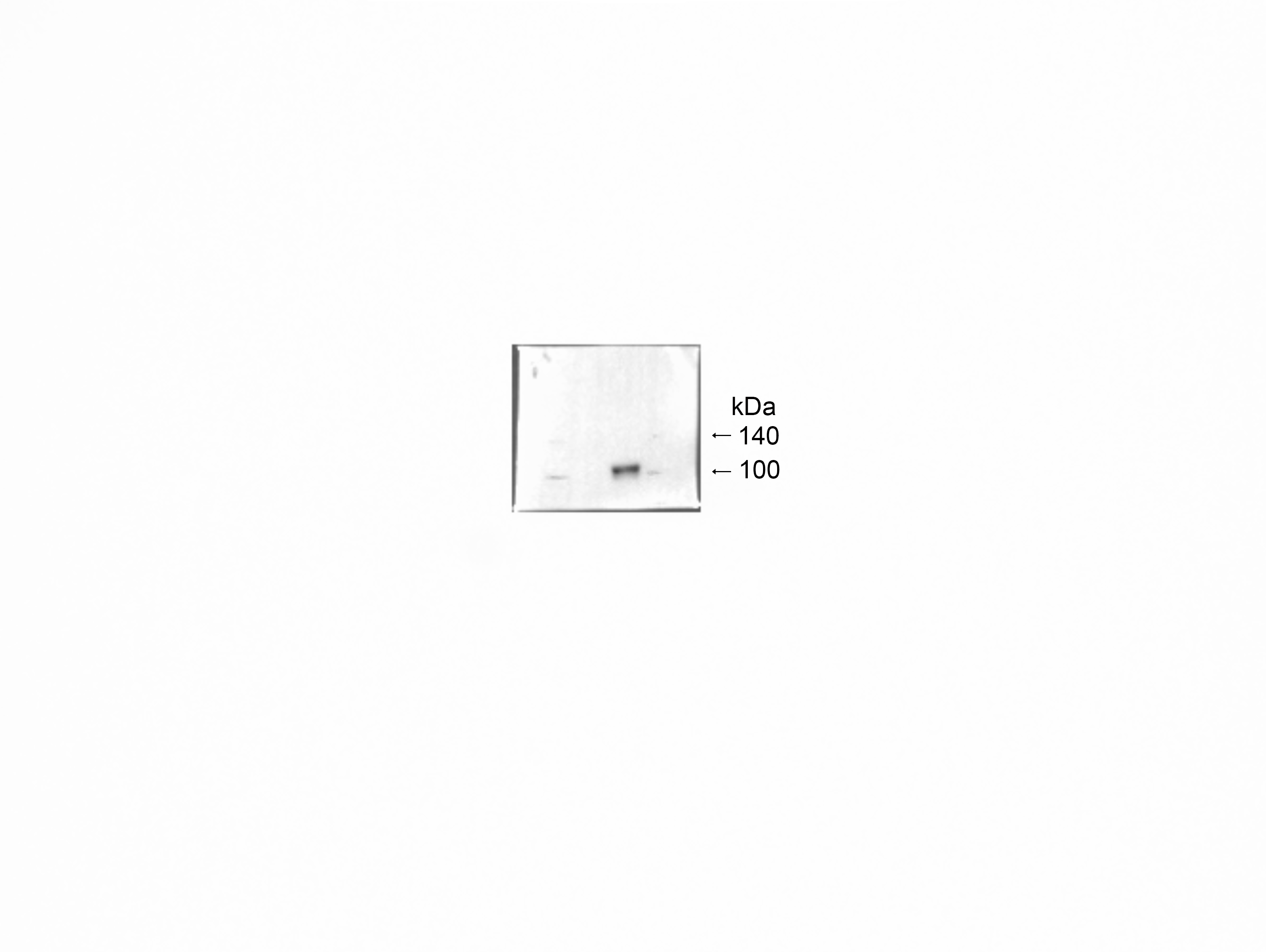

Supplement: Supplementary file 8 — Source data [file 41467_2024_47740_MOESM8_ESM.zip › Source Data/Uncropped blots for Supplementary Fig.1k/anti-LKRSDH.tif]

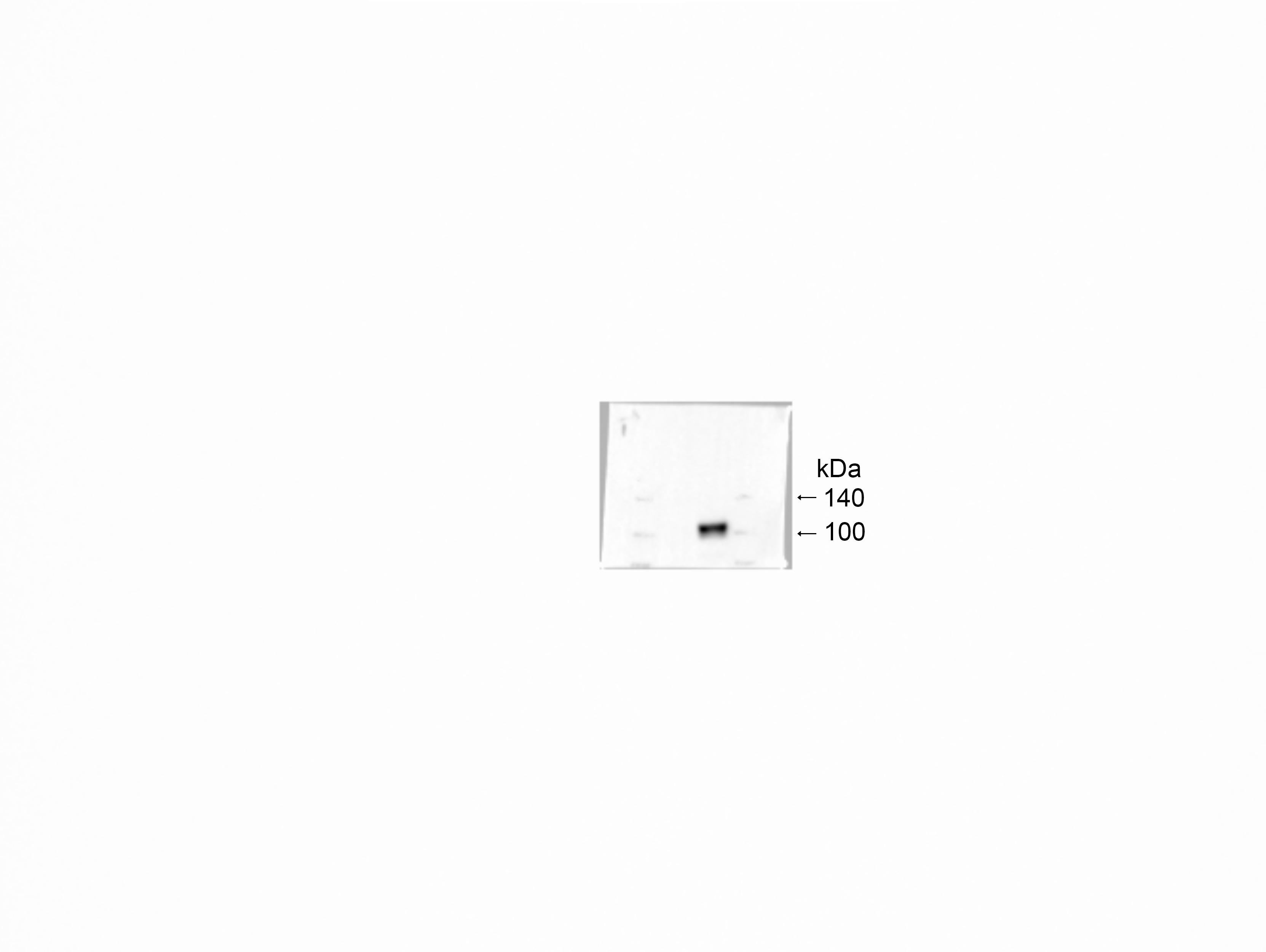

Supplement: Supplementary file 8 — Source data [file 41467_2024_47740_MOESM8_ESM.zip › Source Data/Uncropped blots for Supplementary Fig.1k/anti-V5.tif]

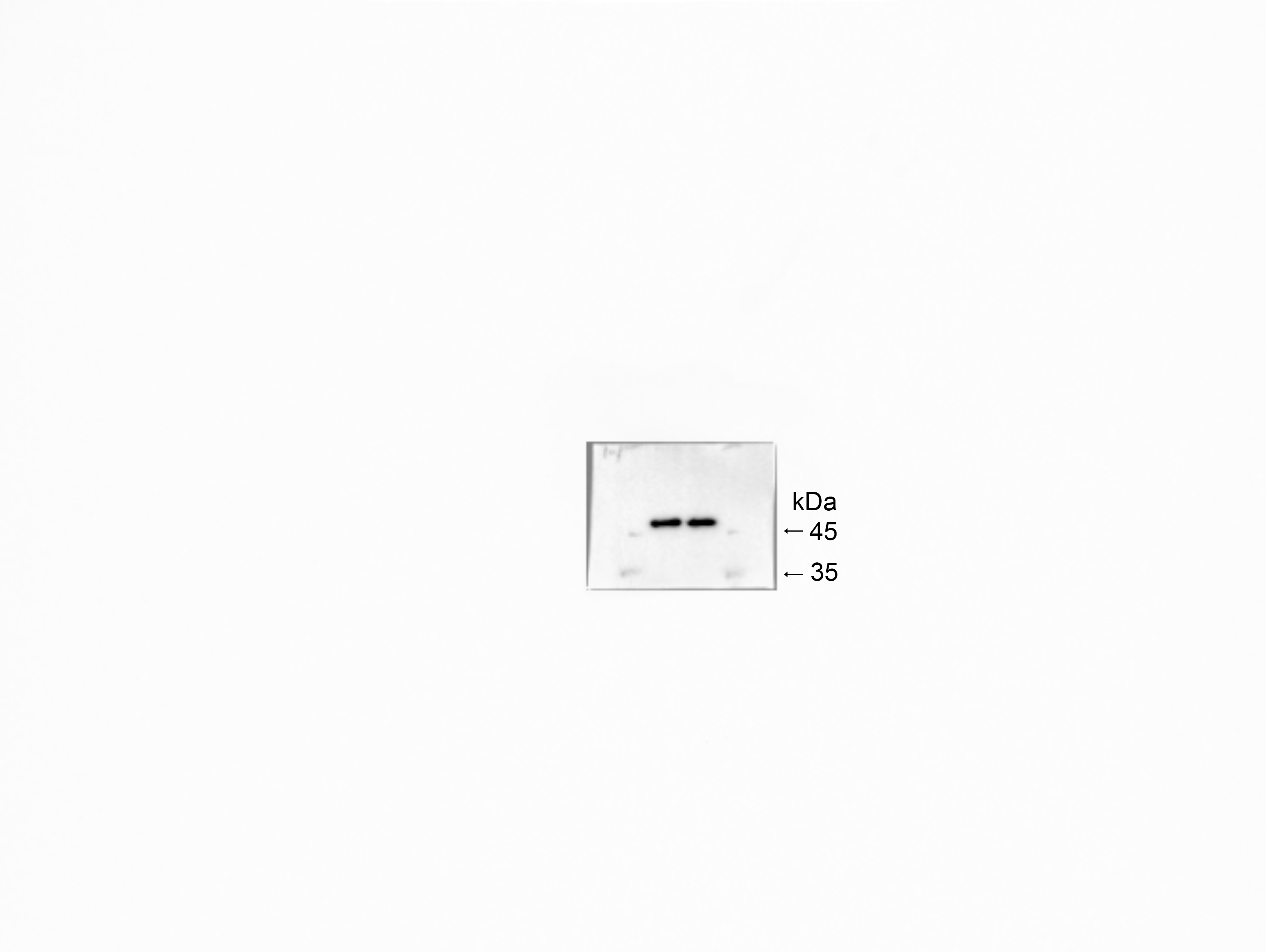

Supplement: Supplementary file 8 — Source data [file 41467_2024_47740_MOESM8_ESM.zip › Source Data/Uncropped blots for Supplementary Fig.1k/anti-β-Tubulin.tif]

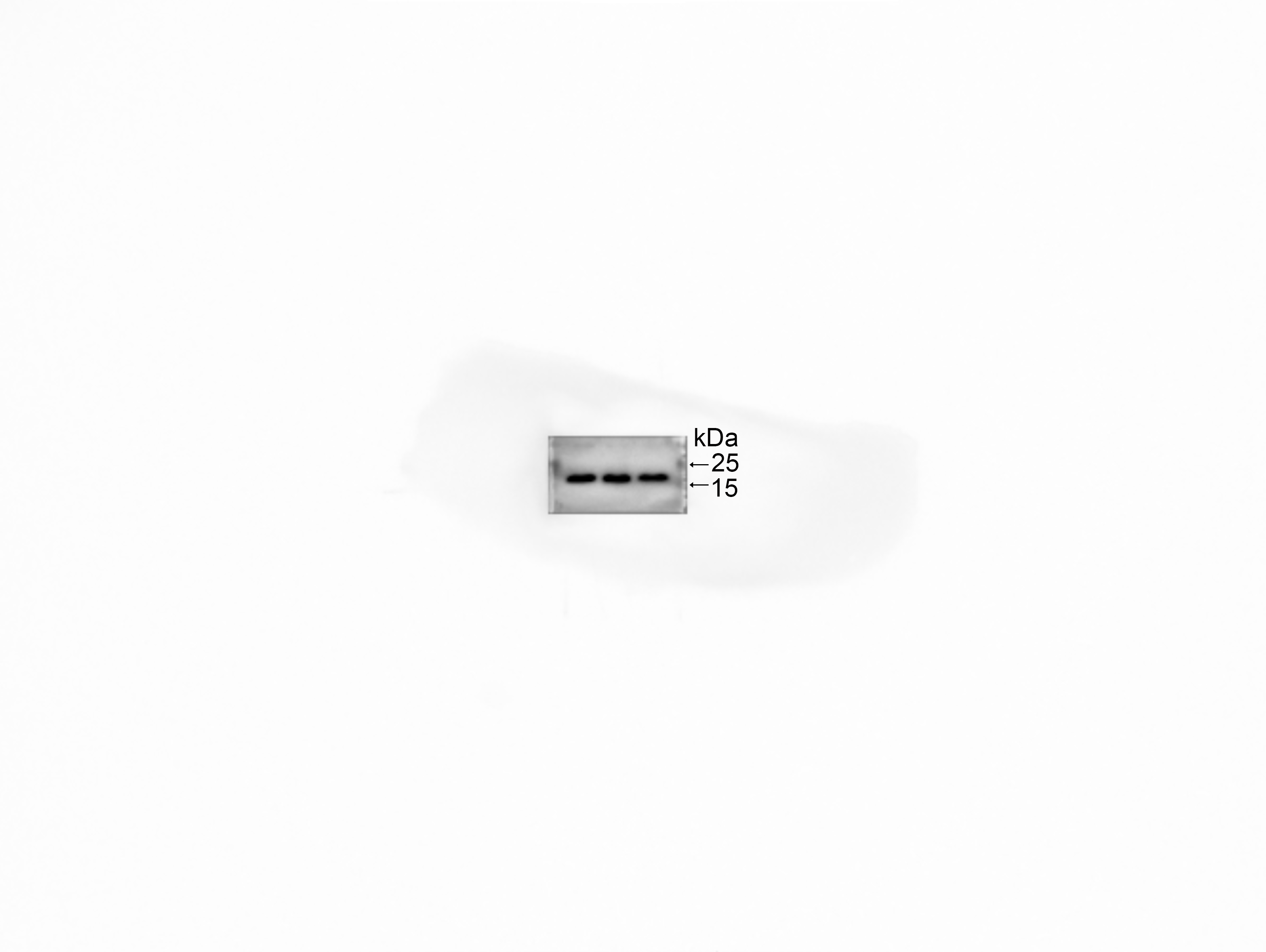

Supplement: Supplementary file 8 — Source data [file 41467_2024_47740_MOESM8_ESM.zip › Source Data/Uncropped blots for Supplementary Fig.6a/Replicate 1 main text/anti-H3.tif]

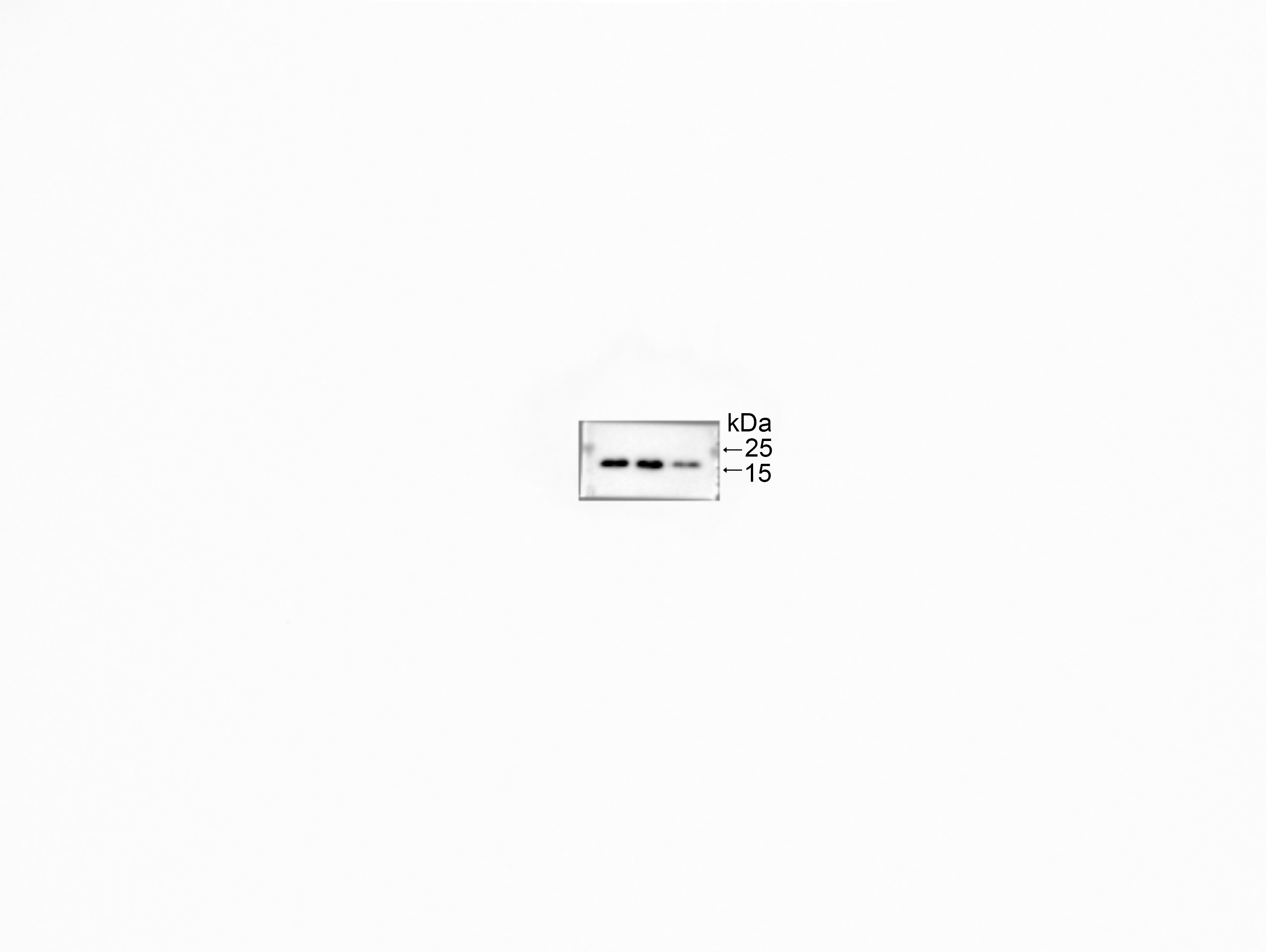

Supplement: Supplementary file 8 — Source data [file 41467_2024_47740_MOESM8_ESM.zip › Source Data/Uncropped blots for Supplementary Fig.6a/Replicate 1 main text/anti-H3R17me2.tif]

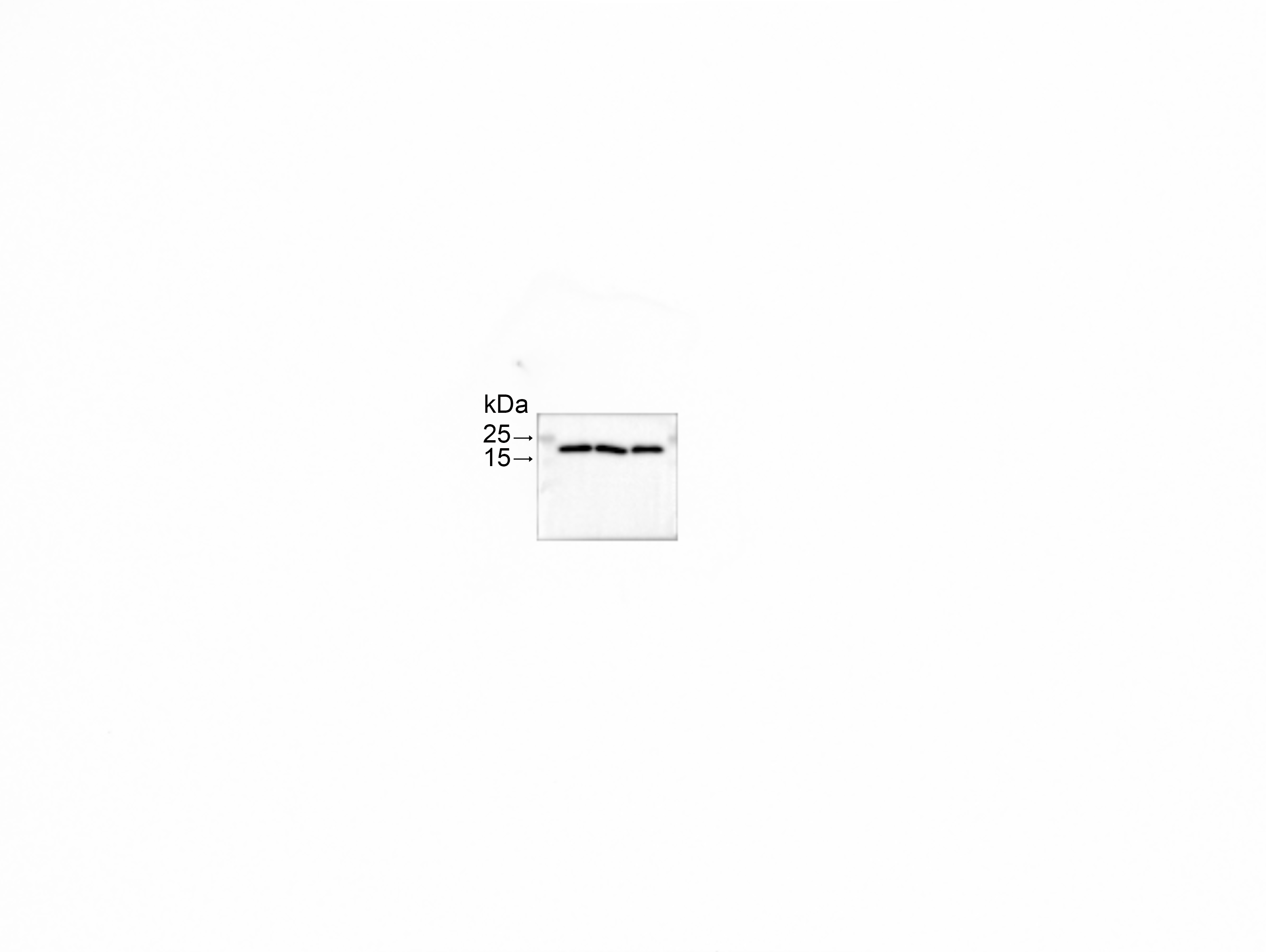

Supplement: Supplementary file 8 — Source data [file 41467_2024_47740_MOESM8_ESM.zip › Source Data/Uncropped blots for Supplementary Fig.6a/Replicate 2/anti-H3.tif]

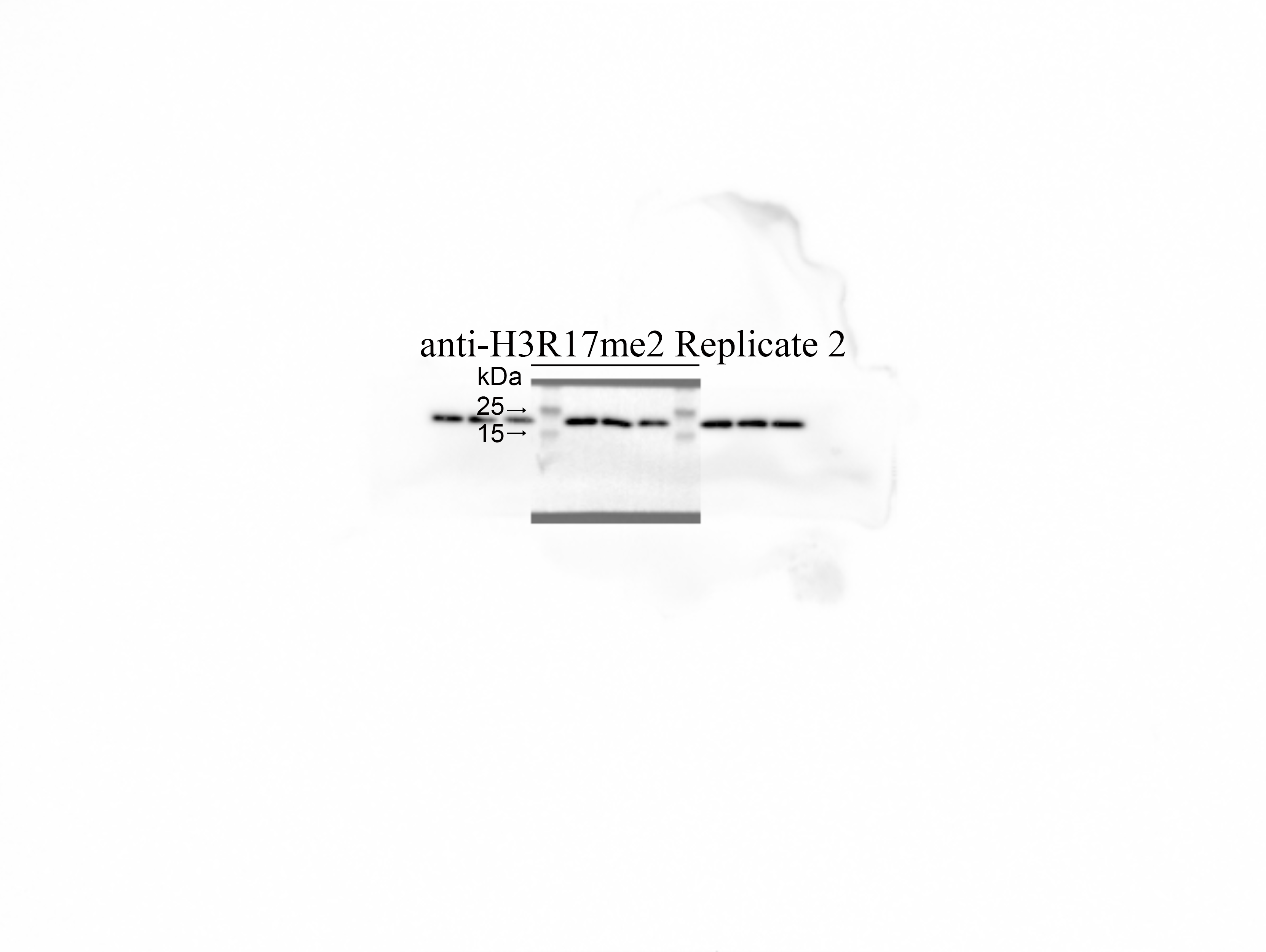

Supplement: Supplementary file 8 — Source data [file 41467_2024_47740_MOESM8_ESM.zip › Source Data/Uncropped blots for Supplementary Fig.6a/Replicate 2/anti-H3R17me2.tif]

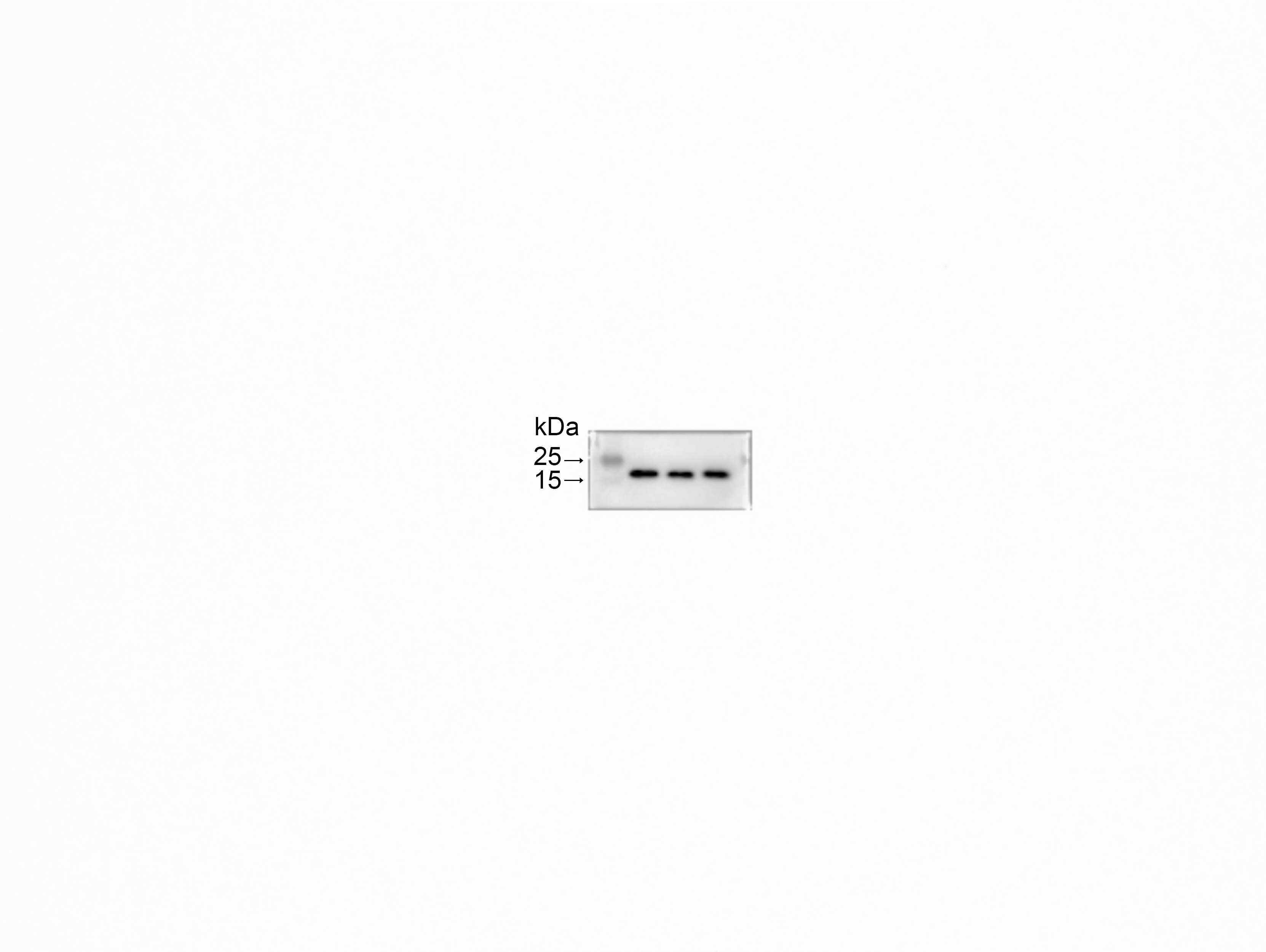

Supplement: Supplementary file 8 — Source data [file 41467_2024_47740_MOESM8_ESM.zip › Source Data/Uncropped blots for Supplementary Fig.6a/Replicate 3/anti-H3.tif]

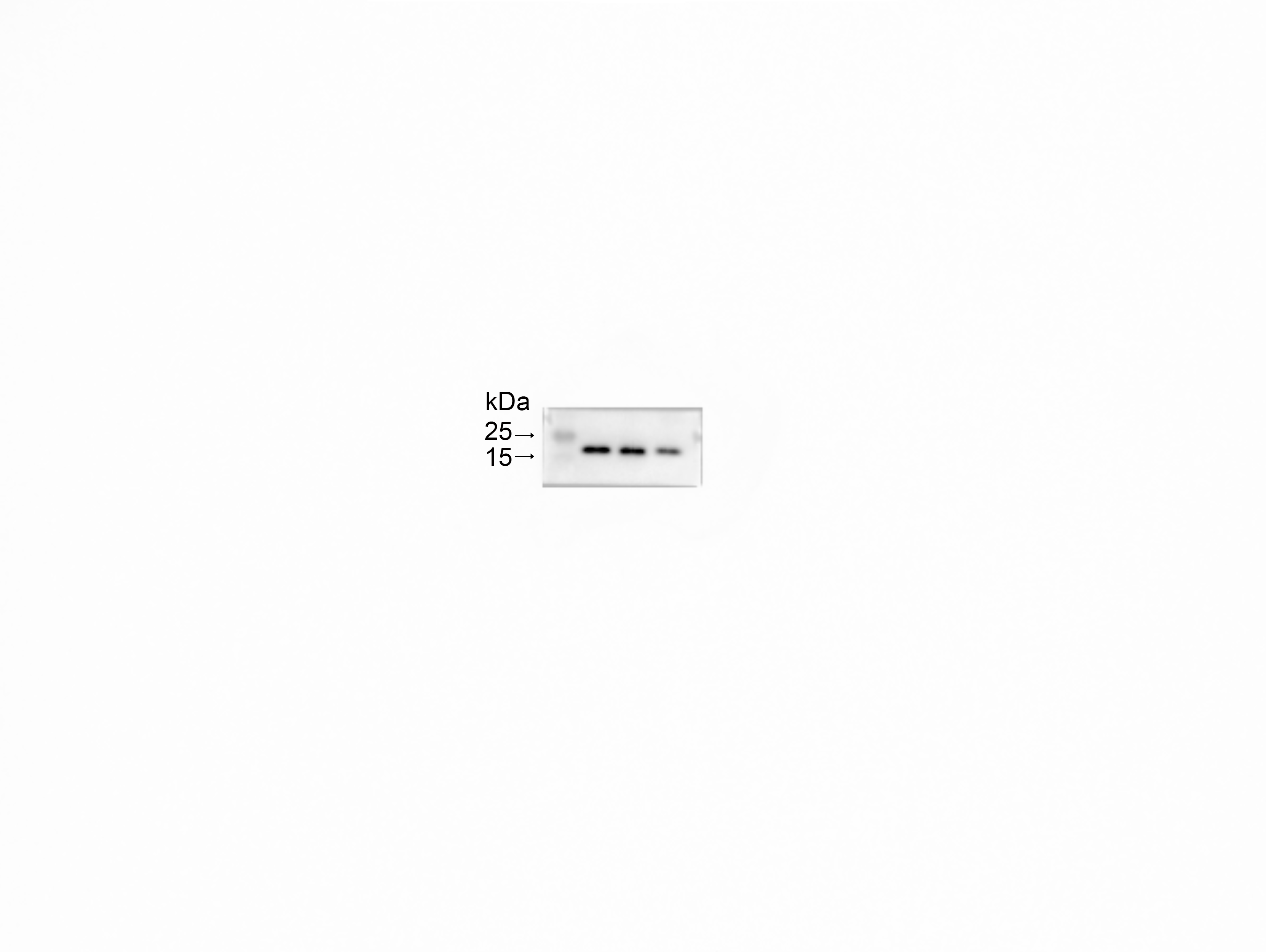

Supplement: Supplementary file 8 — Source data [file 41467_2024_47740_MOESM8_ESM.zip › Source Data/Uncropped blots for Supplementary Fig.6a/Replicate 3/anti-H3R17me2.tif]

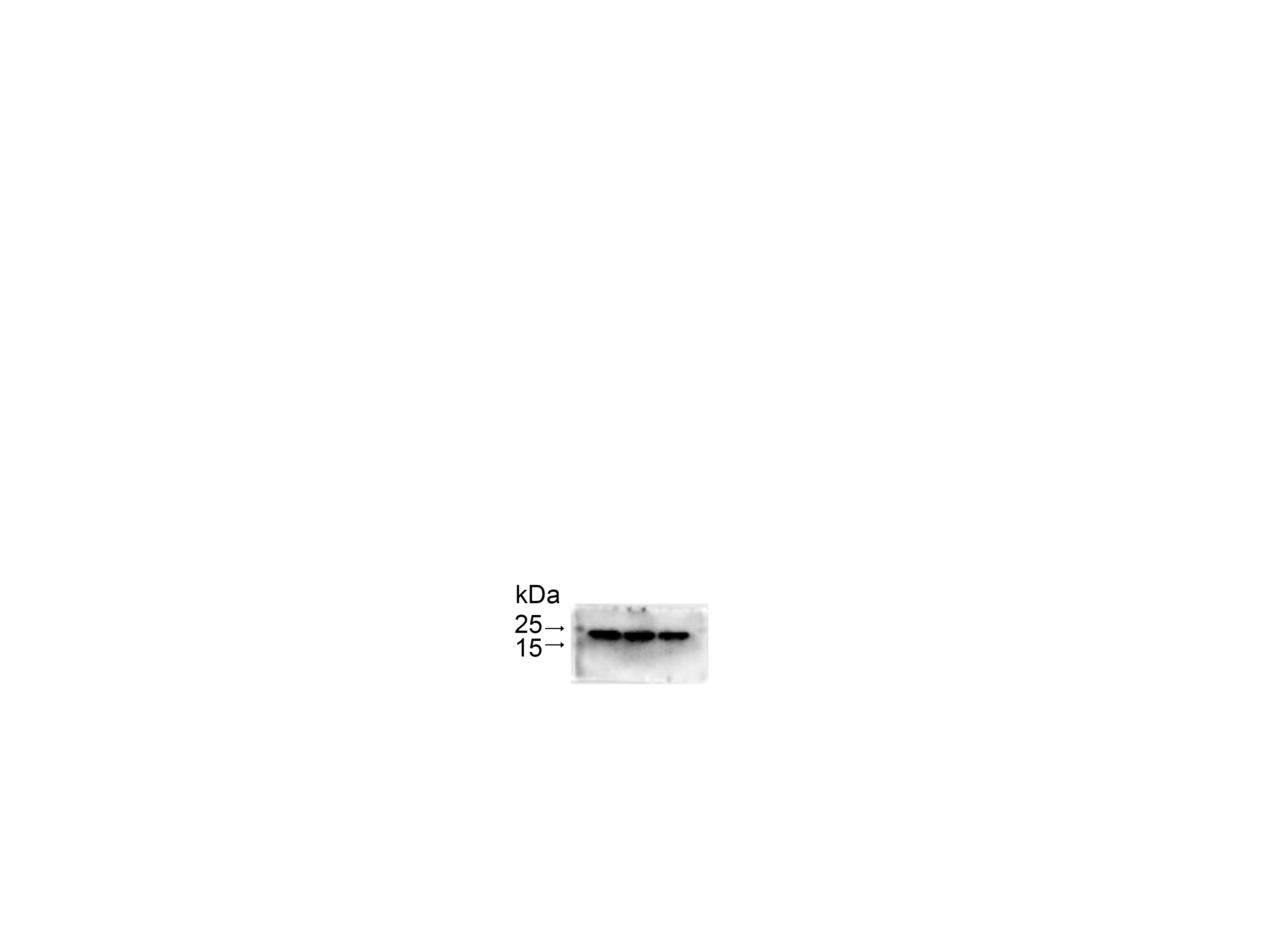

Supplement: Supplementary file 8 — Source data [file 41467_2024_47740_MOESM8_ESM.zip › Source Data/Uncropped blots for Supplementary Fig.6e/Replicate 1/anti-H3.tif]

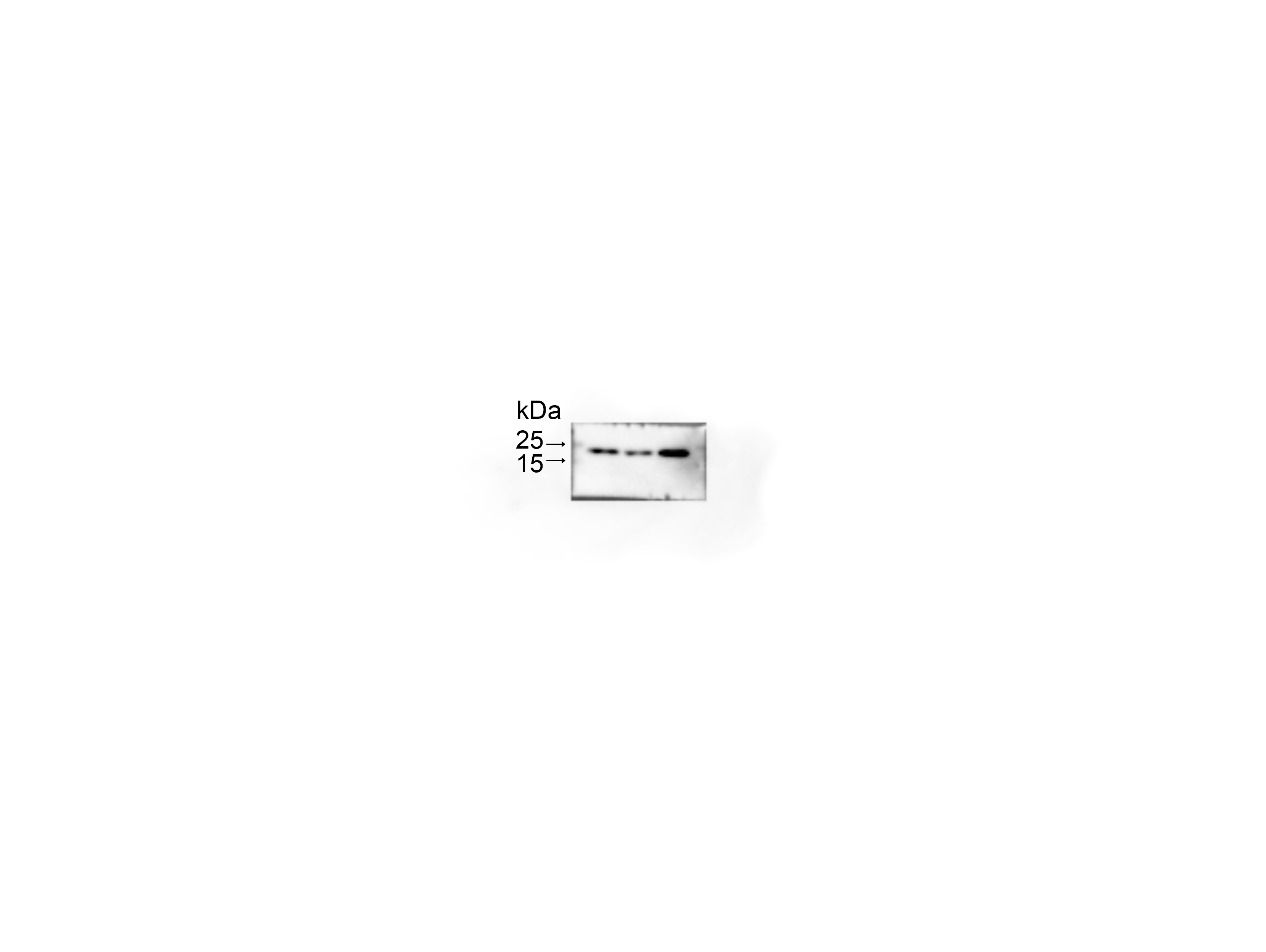

Supplement: Supplementary file 8 — Source data [file 41467_2024_47740_MOESM8_ESM.zip › Source Data/Uncropped blots for Supplementary Fig.6e/Replicate 1/anti-H3R17me2.tif]

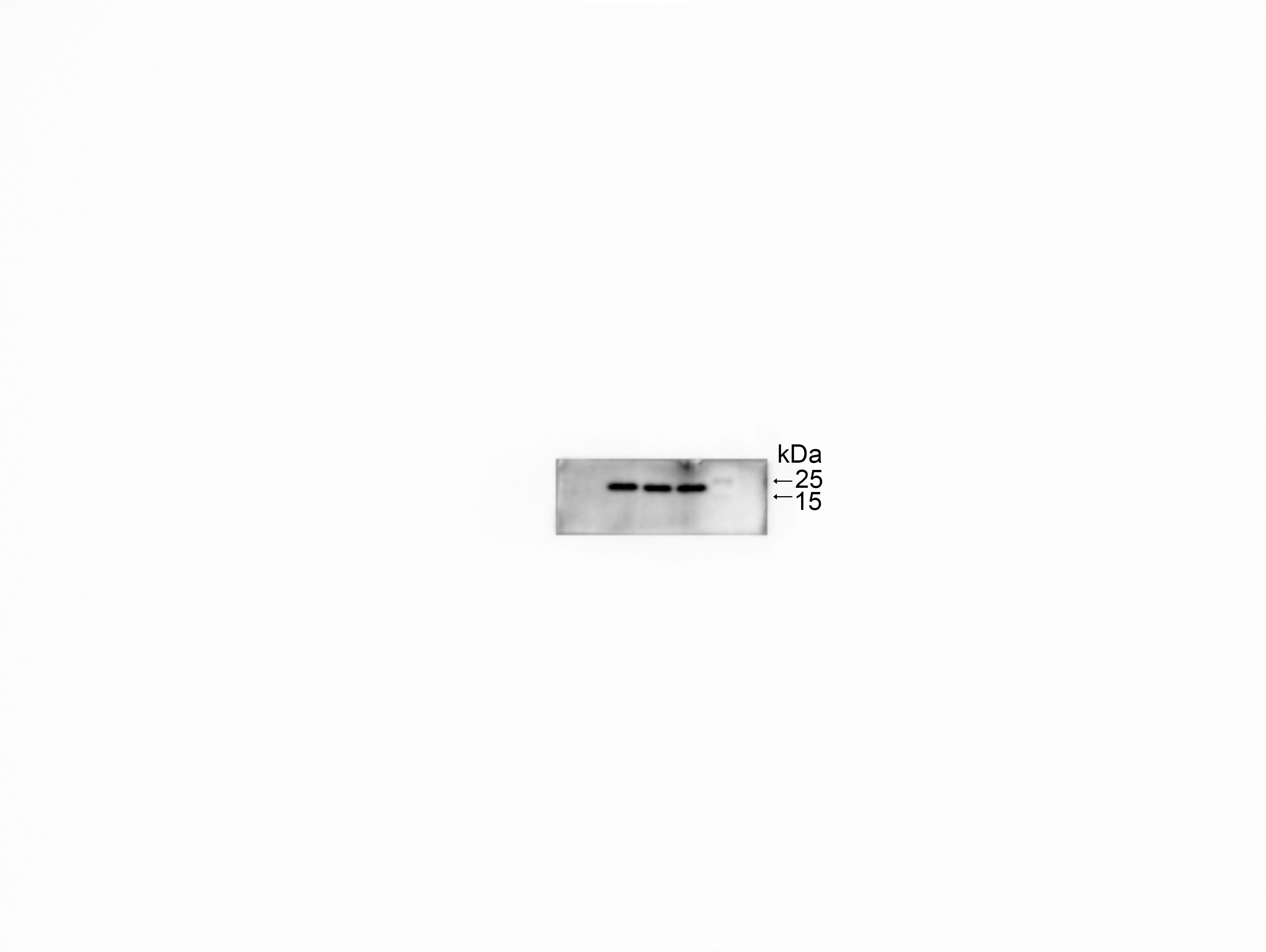

Supplement: Supplementary file 8 — Source data [file 41467_2024_47740_MOESM8_ESM.zip › Source Data/Uncropped blots for Supplementary Fig.6e/Replicate 2 main text/anti-H3.tif]

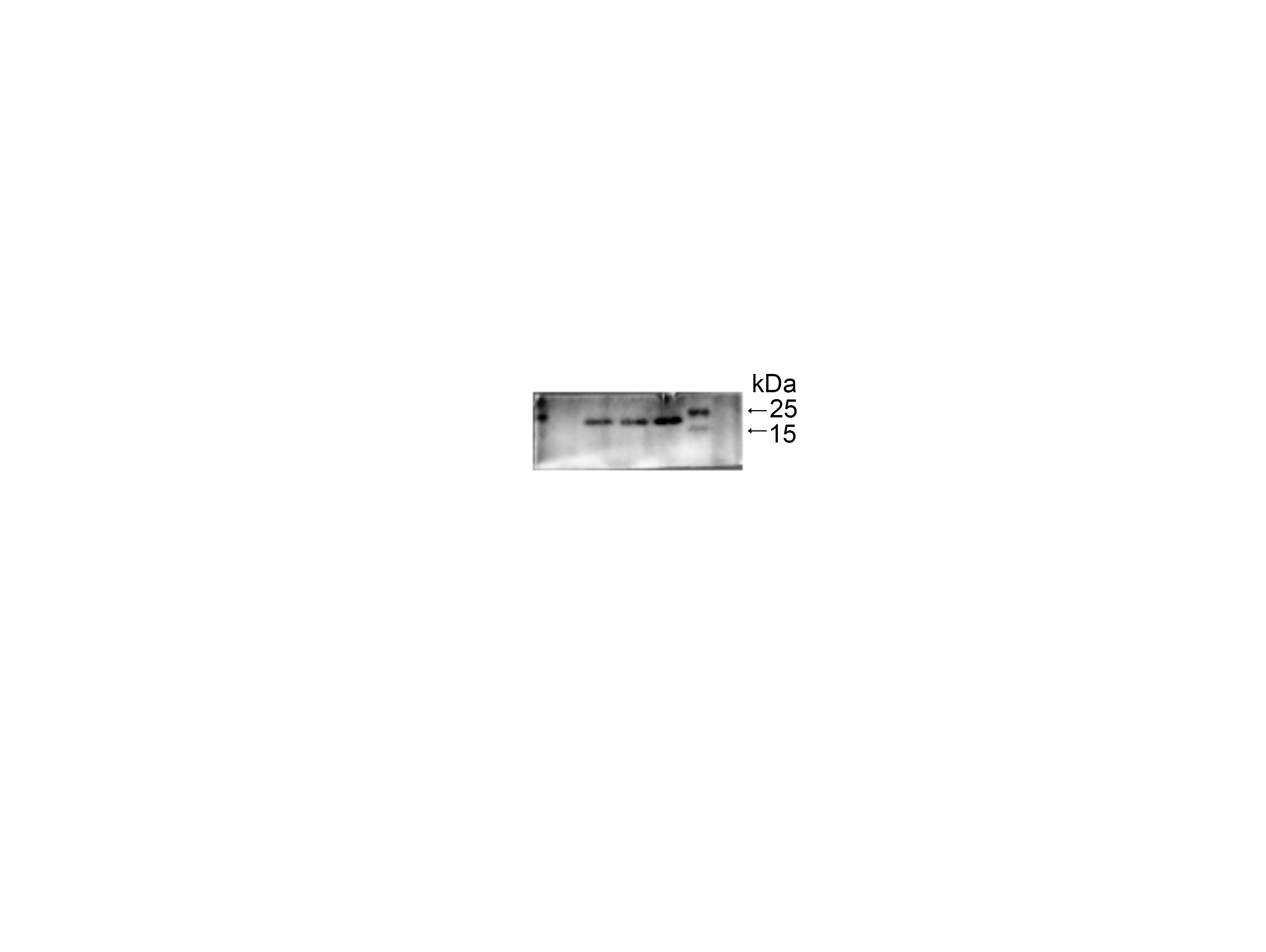

Supplement: Supplementary file 8 — Source data [file 41467_2024_47740_MOESM8_ESM.zip › Source Data/Uncropped blots for Supplementary Fig.6e/Replicate 2 main text/anti-H3R17me2.tif]

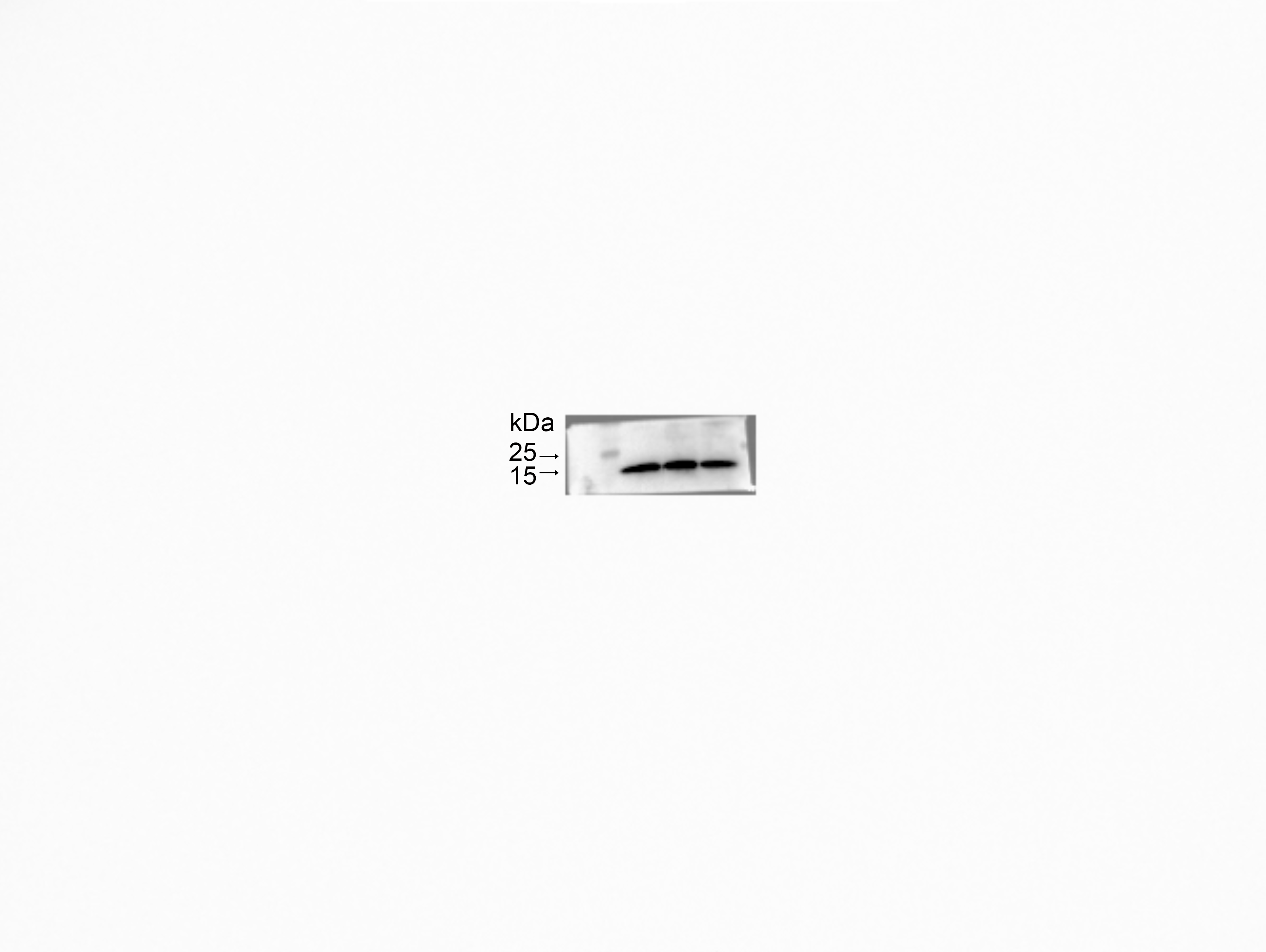

Supplement: Supplementary file 8 — Source data [file 41467_2024_47740_MOESM8_ESM.zip › Source Data/Uncropped blots for Supplementary Fig.6e/Replicate 3/anti-H3.tif]

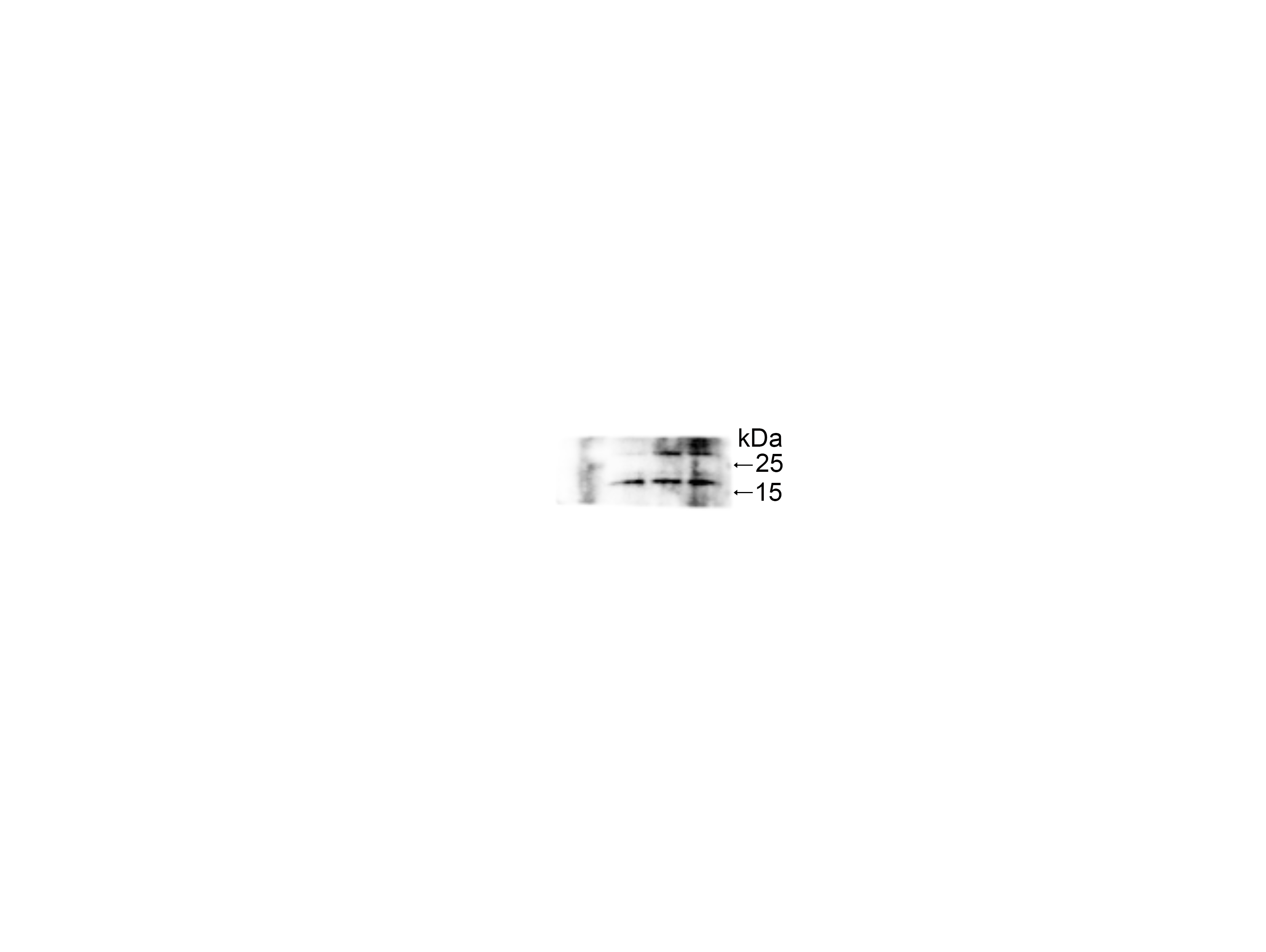

Supplement: Supplementary file 8 — Source data [file 41467_2024_47740_MOESM8_ESM.zip › Source Data/Uncropped blots for Supplementary Fig.6e/Replicate 3/anti-H3R17me2.tif]

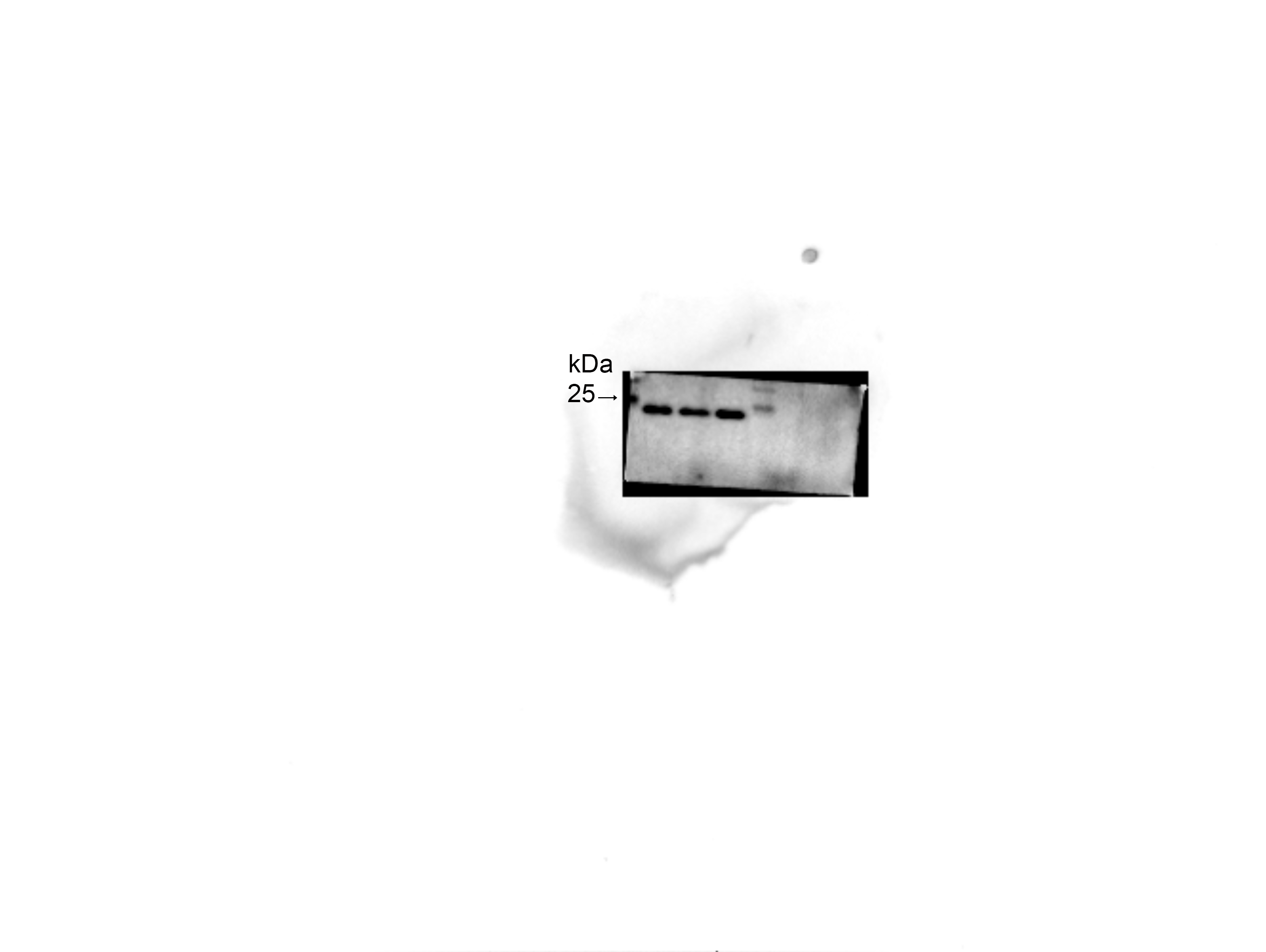

Supplement: Supplementary file 8 — Source data [file 41467_2024_47740_MOESM8_ESM.zip › Source Data/Uncropped blots for Supplementary Fig.7a/Replicate 1/anti-H3.tif]

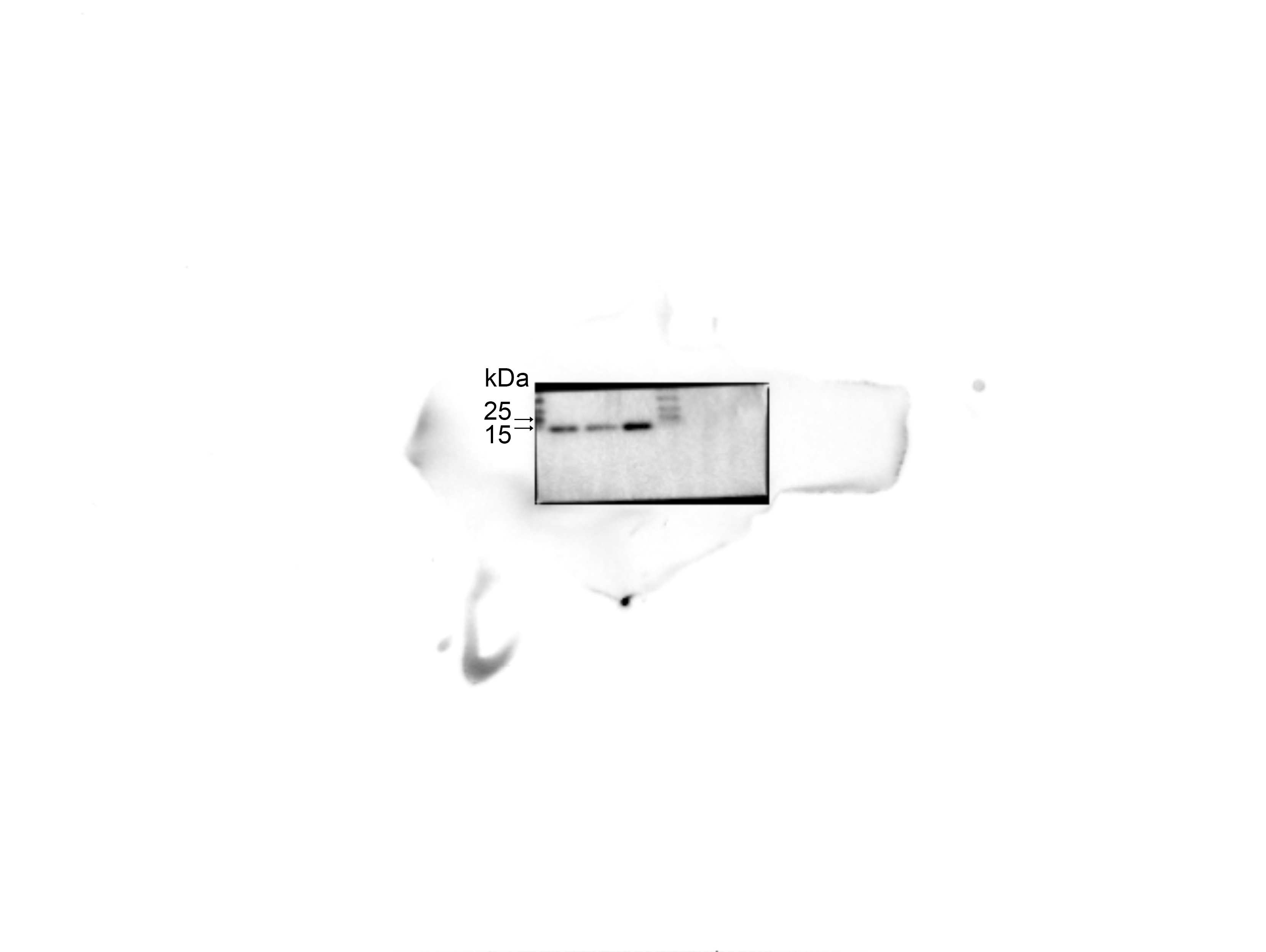

Supplement: Supplementary file 8 — Source data [file 41467_2024_47740_MOESM8_ESM.zip › Source Data/Uncropped blots for Supplementary Fig.7a/Replicate 1/anti-H3K27me3.tif]

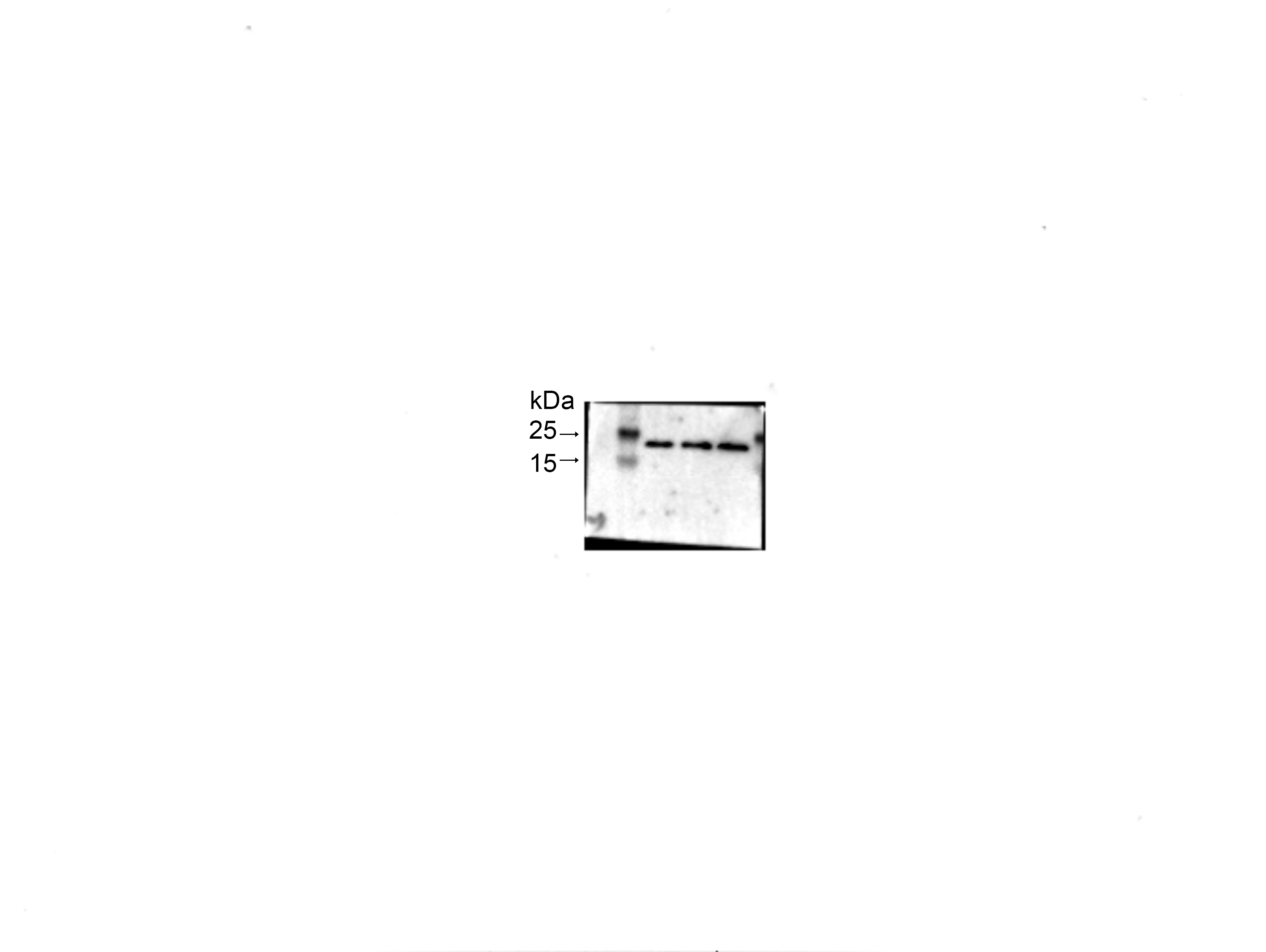

Supplement: Supplementary file 8 — Source data [file 41467_2024_47740_MOESM8_ESM.zip › Source Data/Uncropped blots for Supplementary Fig.7a/Replicate 2 main text/anti-H3.tif]

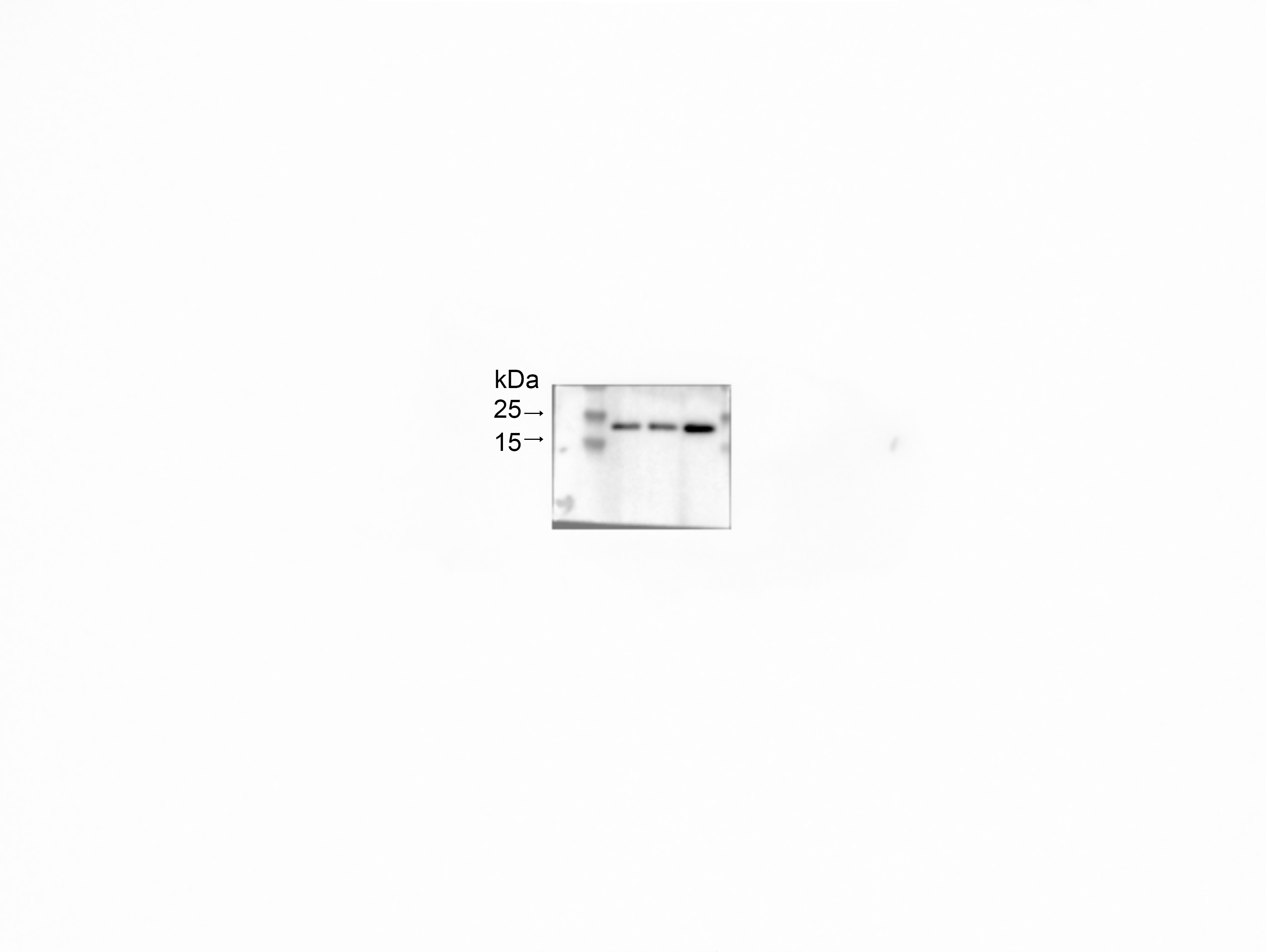

Supplement: Supplementary file 8 — Source data [file 41467_2024_47740_MOESM8_ESM.zip › Source Data/Uncropped blots for Supplementary Fig.7a/Replicate 2 main text/anti-H3K27me3.tif]

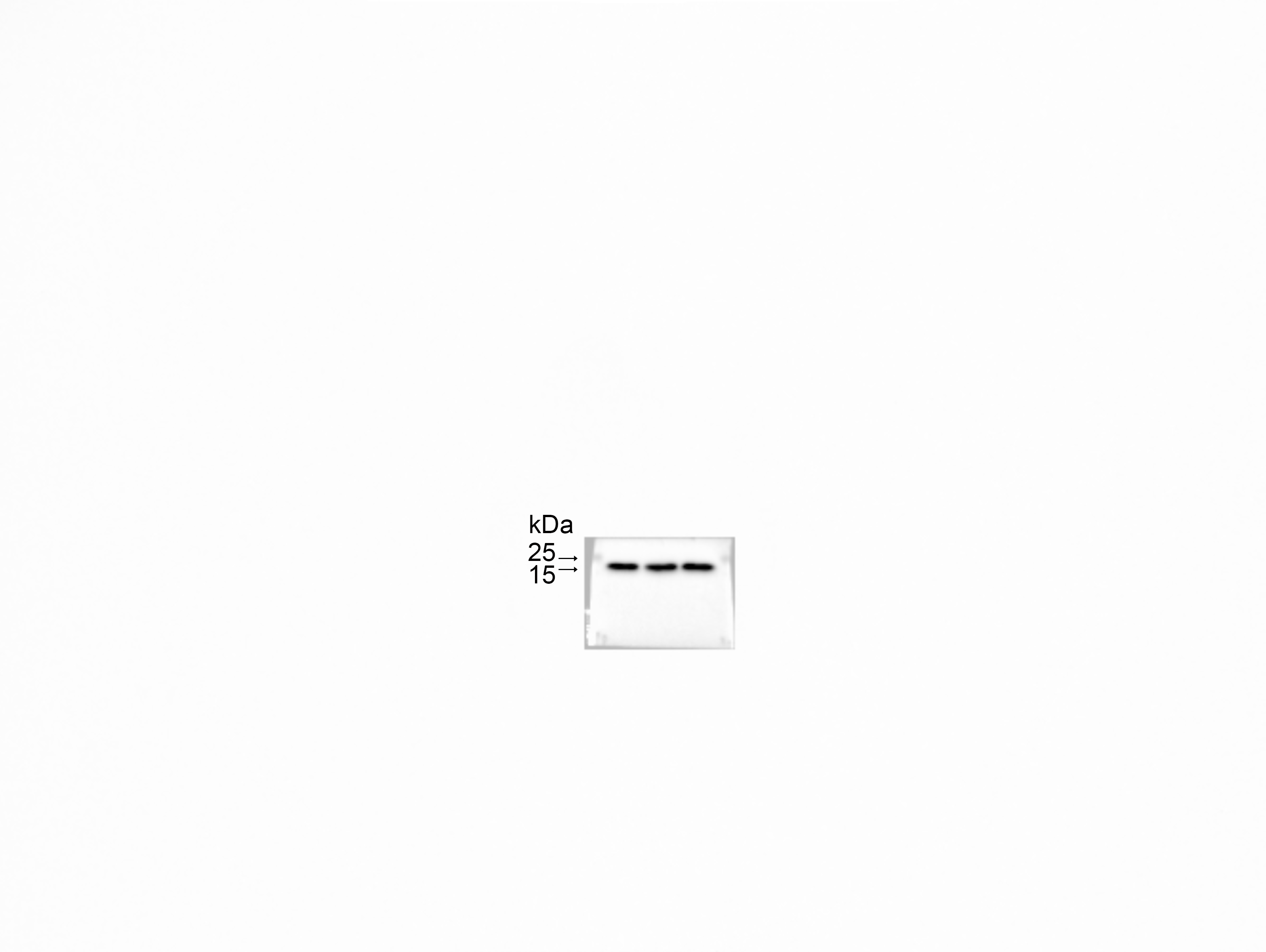

Supplement: Supplementary file 8 — Source data [file 41467_2024_47740_MOESM8_ESM.zip › Source Data/Uncropped blots for Supplementary Fig.7a/Replicate 3/anti-H3.tif]

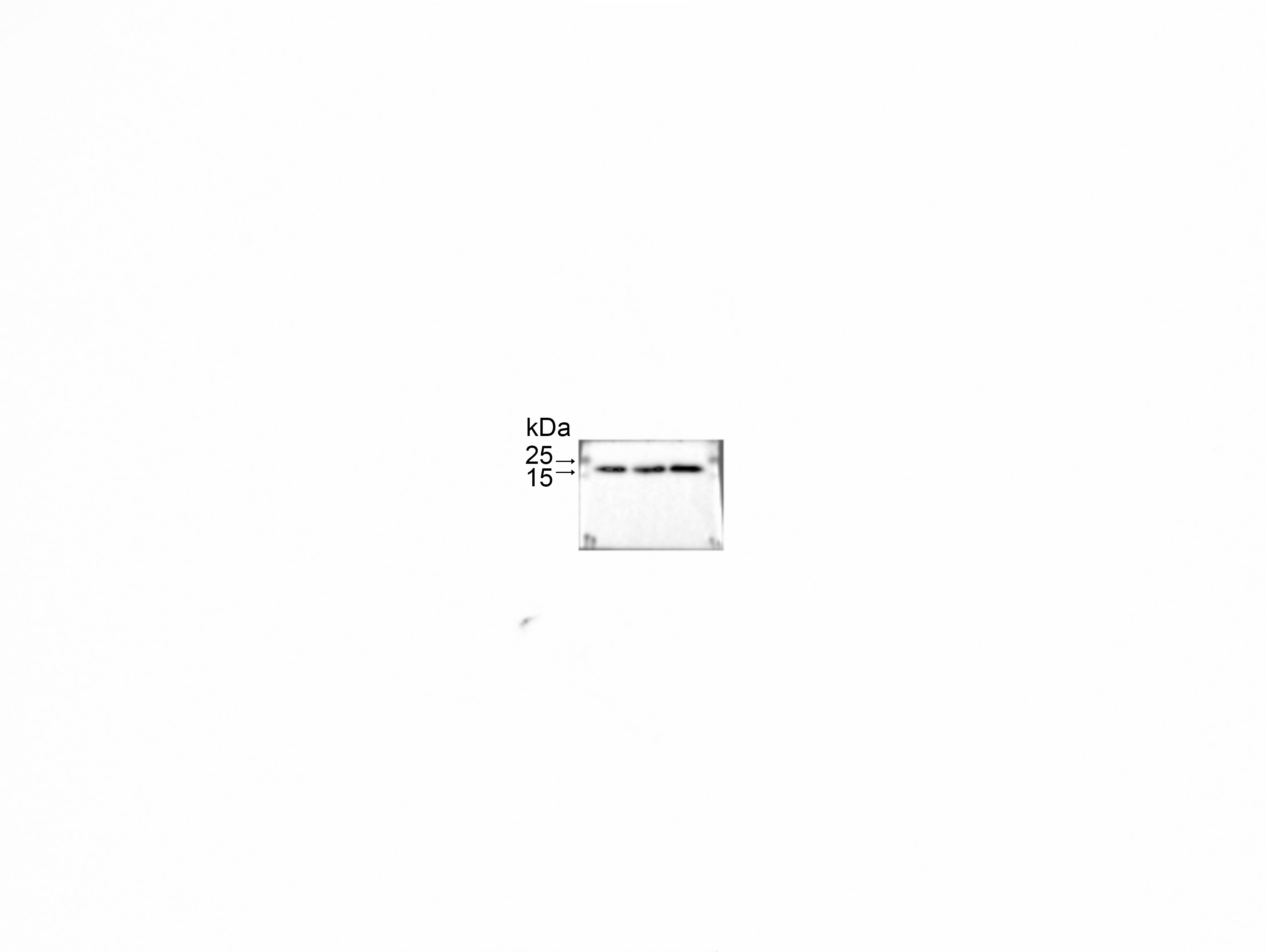

Supplement: Supplementary file 8 — Source data [file 41467_2024_47740_MOESM8_ESM.zip › Source Data/Uncropped blots for Supplementary Fig.7a/Replicate 3/anti-H3K27me3.tif]

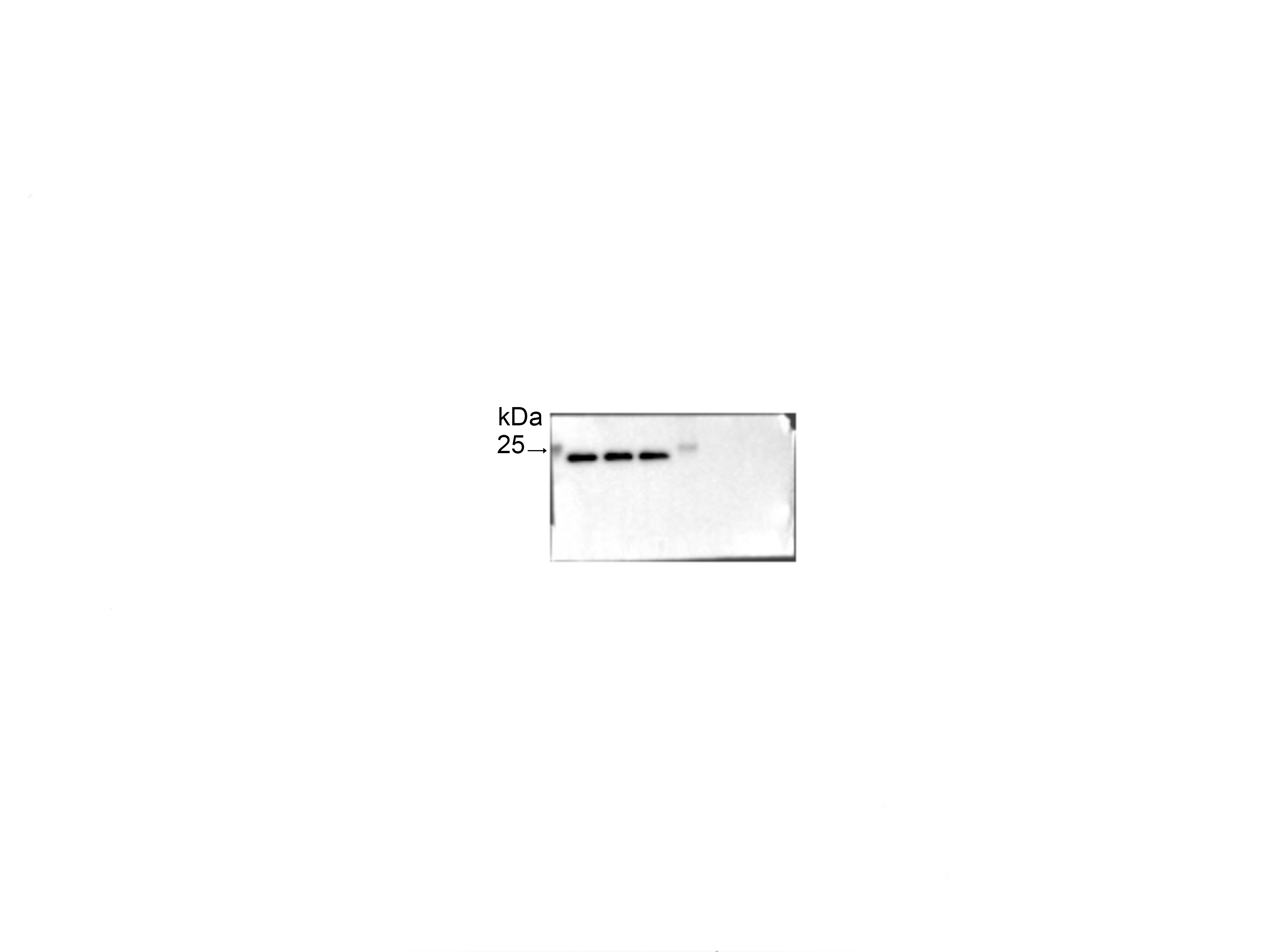

Supplement: Supplementary file 8 — Source data [file 41467_2024_47740_MOESM8_ESM.zip › Source Data/Uncropped blots for Supplementary Fig.7h/Replicate 1/anti-H3.tif]

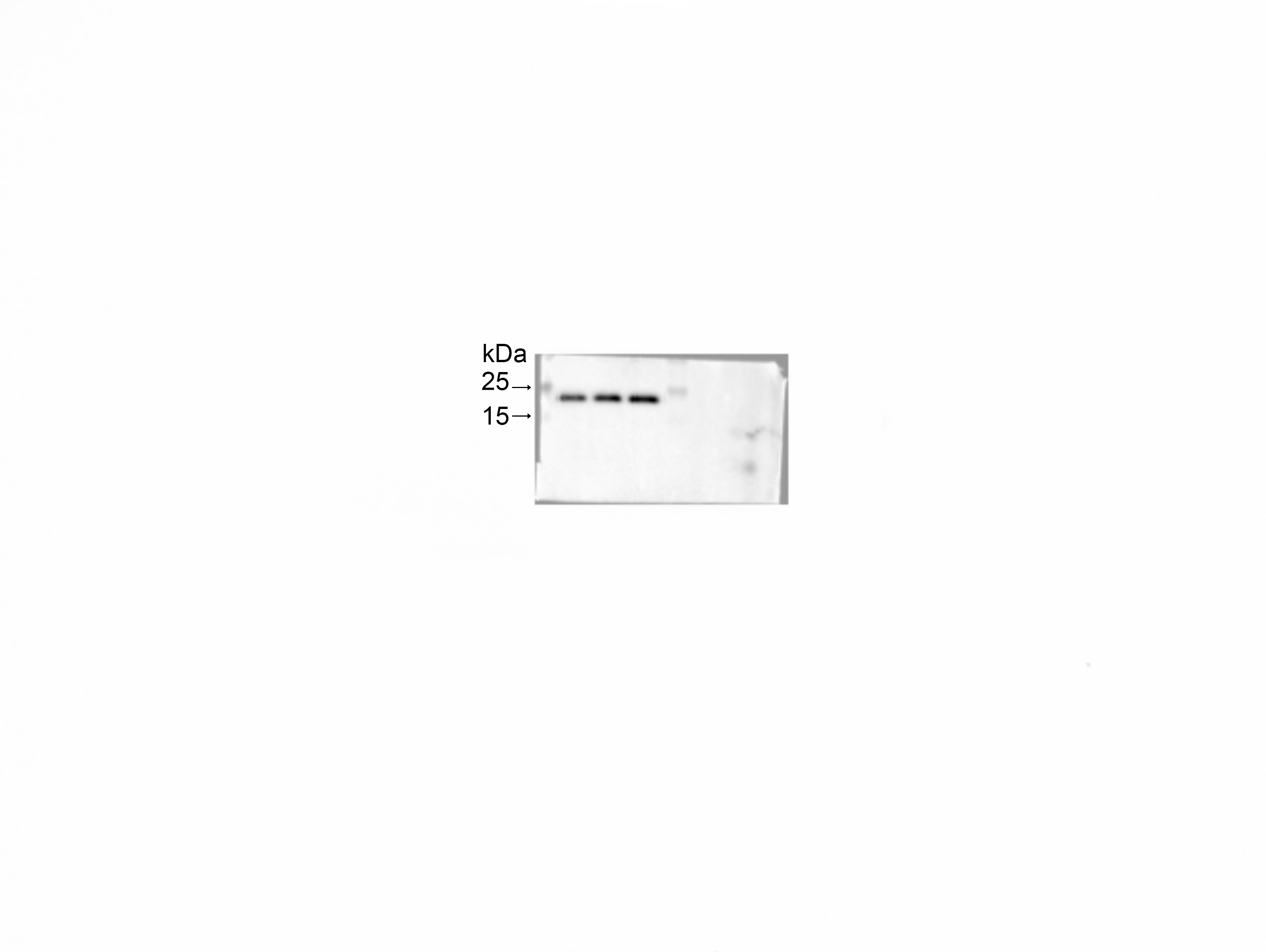

Supplement: Supplementary file 8 — Source data [file 41467_2024_47740_MOESM8_ESM.zip › Source Data/Uncropped blots for Supplementary Fig.7h/Replicate 1/anti-H3K27me3.tif]

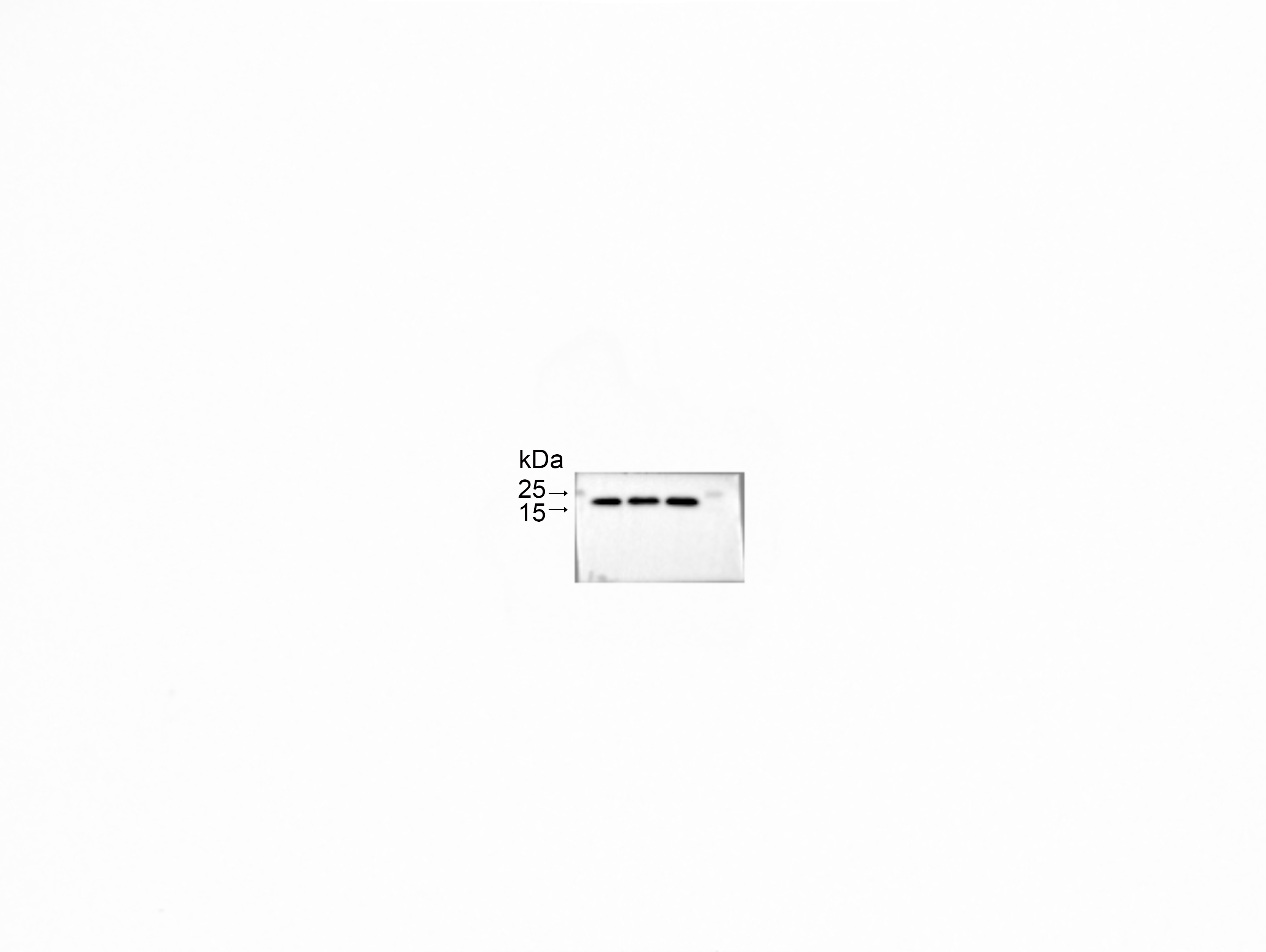

Supplement: Supplementary file 8 — Source data [file 41467_2024_47740_MOESM8_ESM.zip › Source Data/Uncropped blots for Supplementary Fig.7h/Replicate 2 main text/anti-H3.tif]

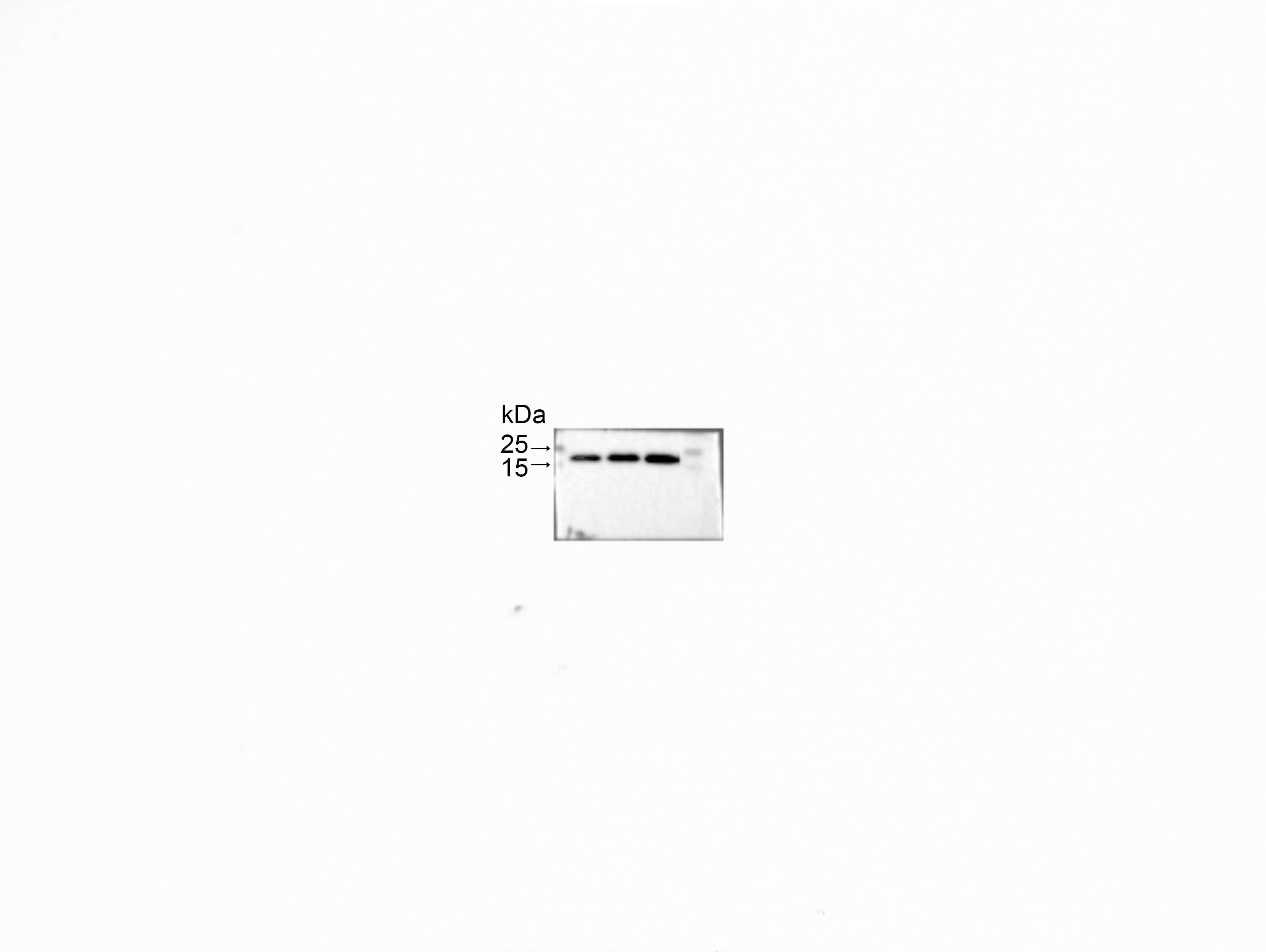

Supplement: Supplementary file 8 — Source data [file 41467_2024_47740_MOESM8_ESM.zip › Source Data/Uncropped blots for Supplementary Fig.7h/Replicate 2 main text/anti-H3K27me3.tif]

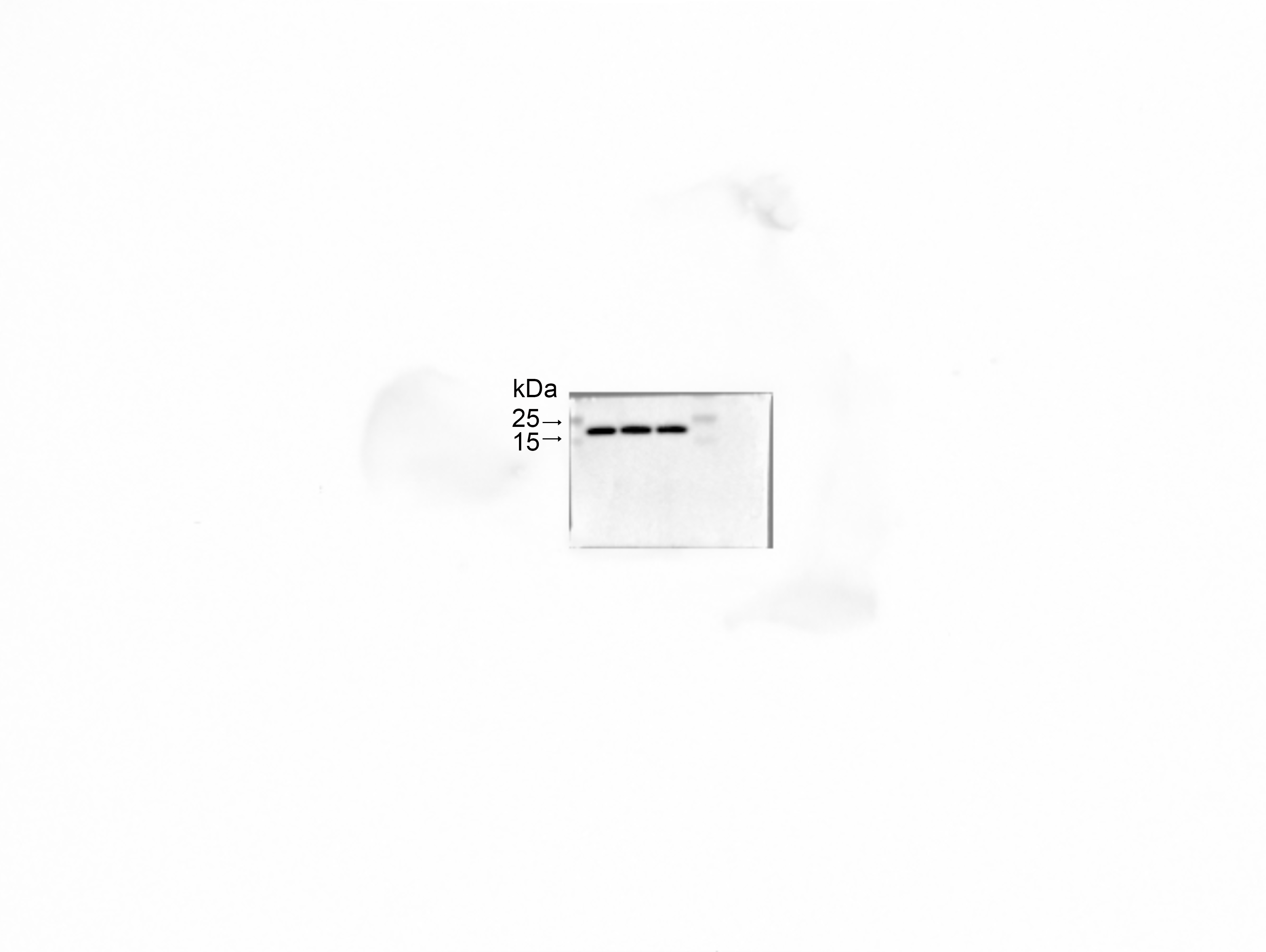

Supplement: Supplementary file 8 — Source data [file 41467_2024_47740_MOESM8_ESM.zip › Source Data/Uncropped blots for Supplementary Fig.7h/Replicate 3/anti-H3.tif]

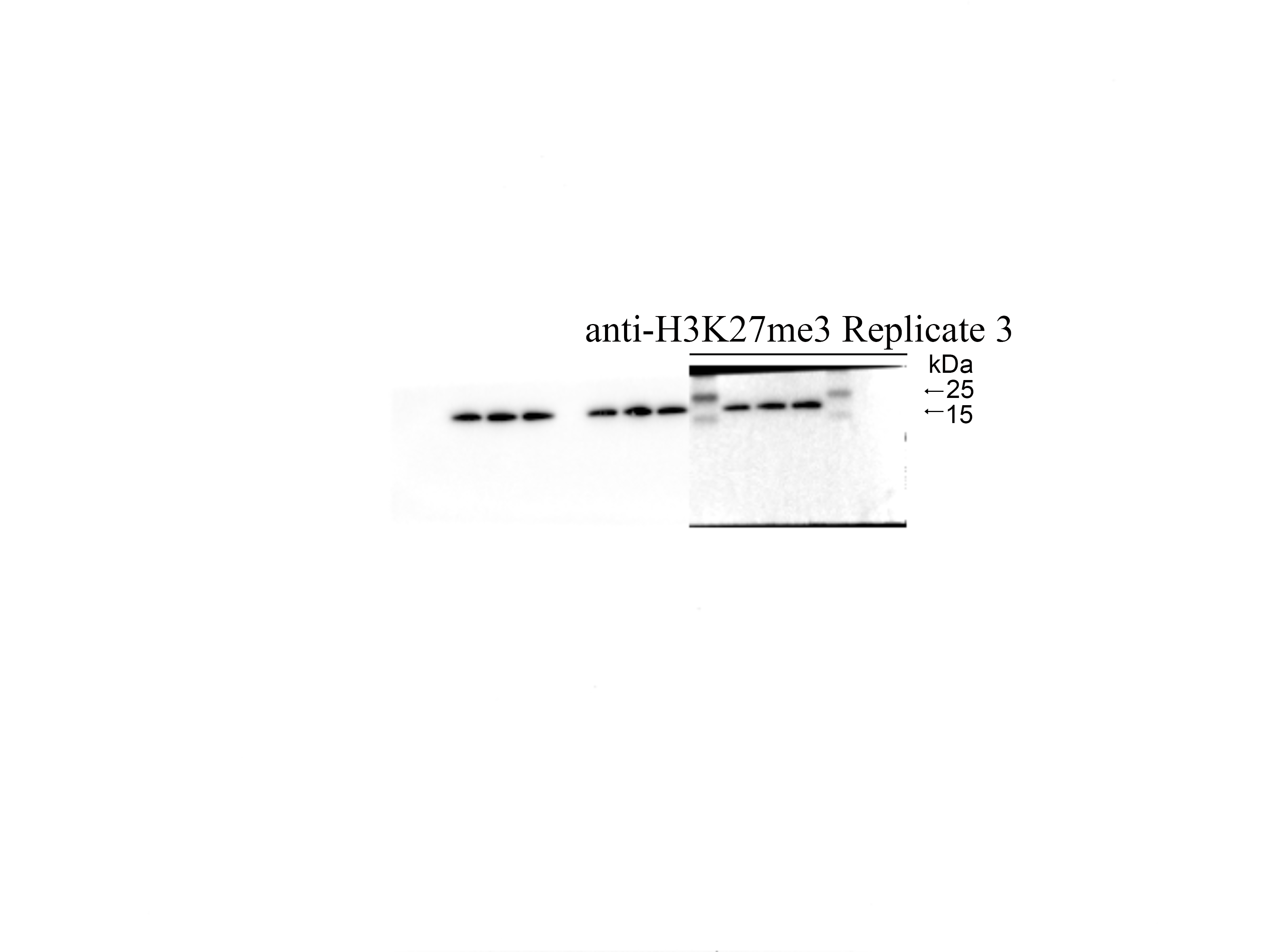

Supplement: Supplementary file 8 — Source data [file 41467_2024_47740_MOESM8_ESM.zip › Source Data/Uncropped blots for Supplementary Fig.7h/Replicate 3/anti-H3K27me3.tif]
